# Butyric acid and valeric acid attenuate stress-induced ferroptosis and depressive-like behaviors by suppressing hippocampal neuroinflammation

**DOI:** 10.1186/s12967-025-06950-0

**Published:** 2025-09-02

**Authors:** Xiaoying Ma, Weibo Shi, Zhen Wang, Shujin Li, Rufei Ma, Weihao Zhu, Lin Wu, Xiaowei Feng, Bin Cong, Yingmin Li

**Affiliations:** Collaborative Innovation Center of Forensic Medical Molecular Identification, Hebei Key Laboratory of Forensic Medicine, Department of Forensic Medicine, Hebei Medical University, Shijiazhuang, 050017 China

**Keywords:** Stress, Hippocampal neuronal ferroptosis, Depression, Short-chain fatty acid, Neuroinflammation

## Abstract

**Background:**

Depression is closely associated with stress-induced hippocampal damage and dysfunction. Emerging evidence demonstrates that the gut microbiota and its metabolites, acting as probiotics or prebiotics, can modulate brain structure and function via the gut-brain axis, thereby offering therapeutic potential for ameliorating related neurological and psychiatric disorders. This study delves into the contribution of the gut microbiota and its metabolites to stress-induced ferroptosis of hippocampal neurons and the associated molecular pathways.

**Methods:**

This study used time-course stress paradigms combined with ferroptosis inhibitors to identify hippocampal neuronal ferroptosis. Fecal microbiota transplantation were conducted to analyze the role of gut microbiota in this process. Subsequently, 16 S rDNA sequencing and metabolomics techniques were applied to identify key gut microbiota and metabolites. Metabolites intervention were performed to examine their causal relationship with neuronal ferroptosis. Finally, we used histochemical and molecular assays to assess both intestinal and blood-brain barrier integrity as well as inflammation in peripheral blood and hippocampal tissue, along with GPR41/RhoA/Rock1 pathway changes, to preliminarily investigate the molecular mechanisms underlying stress-induced hippocampal neuronal ferroptosis.

**Results:**

We demonstrated that stress triggered hippocampal neuronal ferroptosis and subsequent depressive-like behaviors in mice. Fecal microbiota transplantation successfully replicated the ferroptosis phenotype. Butyric acid and valeric acid were identified as key metabolites significantly reduced in the serum of acutely and chronically stressed mice, respectively. Intervention with these metabolites markedly alleviated ferroptosis. Furthermore, valerate intervention increased hippocampal GPR41 expression and significantly suppressed the pro-inflammatory RhoA/Rock1 pathway in chronically stressed mice, thereby reducing neuroinflammation and ameliorating neuronal ferroptosis. However, butyrate intervention showed no significant effect on the GPR41/RhoA/Rock1 pathway.

**Conclusion:**

Stress induces ferroptosis in hippocampal neurons, where reduced abundance of short-chain fatty acid-producing bacteria plays a key role. Key metabolites butyric acid and valeric acid alleviate neuroinflammation to improve ferroptosis via the gut-brain axis in acute and chronic stress, respectively. Specifically, valeric acid exerts neuroprotective effect through the GPR41/RhoA/Rock1 pathway, whereas butyric acid-mediated protection likely operates through alternative mechanisms.

**Graphical abstract:**

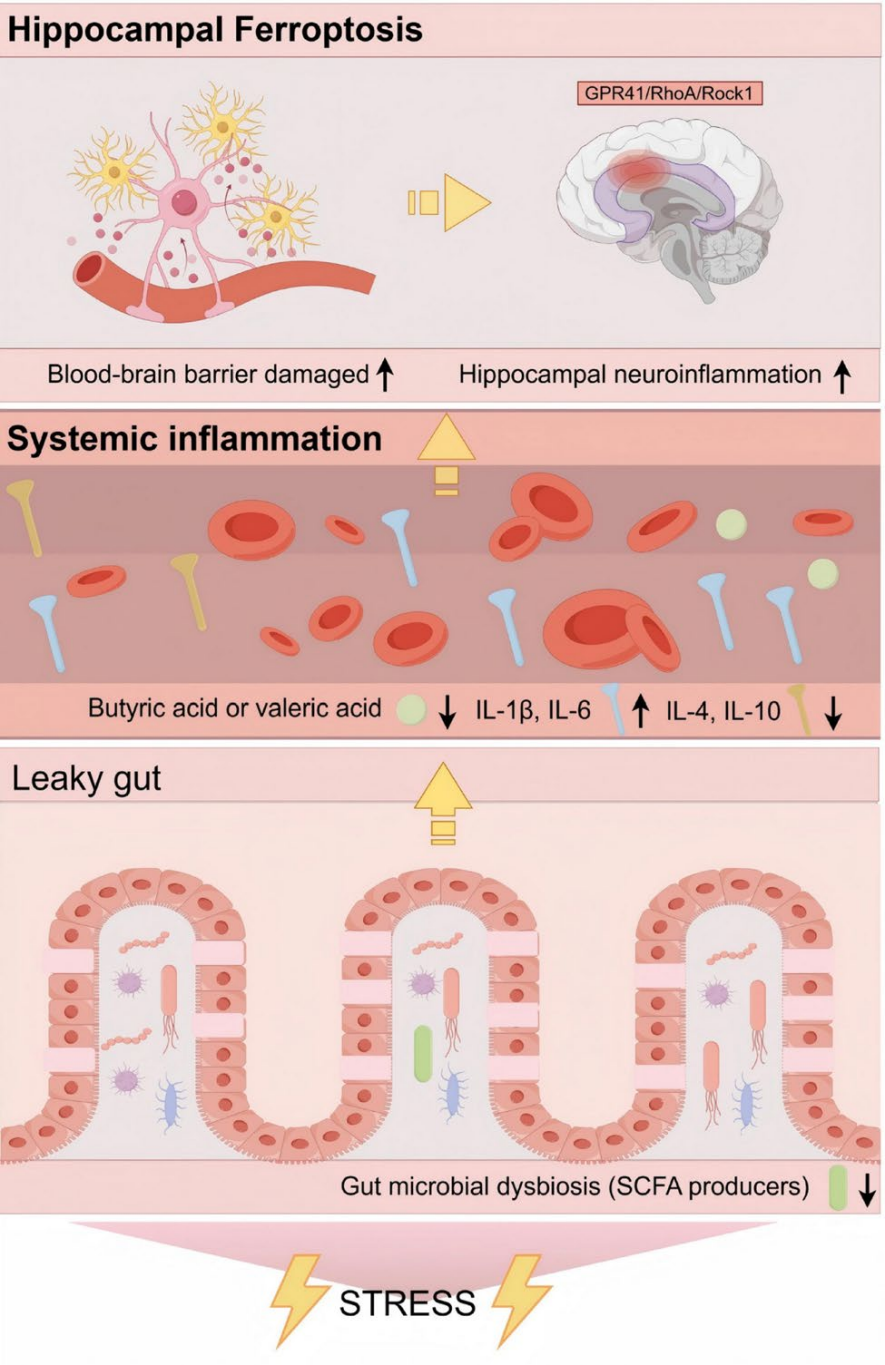

**Supplementary Information:**

The online version contains supplementary material available at 10.1186/s12967-025-06950-0.

## Background

Depression, a leading mental health disorder worldwide, affects approximately 5% of adults annually worldwide, as reported by the Lancet-World Psychiatric Association Commission on Depression [1]. This condition imposes substantial socioeconomic burdens due to its chronic nature and functional impairments when inadequately managed [2]. Stress, a key precipitating factor, triggers maladaptive neurophysiological responses that may progress to depression if unresolved [3]. Nevertheless, the molecular mechanisms linking stress to depressive pathogenesis remain incompletely understood.

The hippocampus, a limbic structure critical for memory consolidation and emotional regulation [4], is particularly vulnerable to the stress-induced structural and functional impairments implicated in depression [5]. Emerging evidence suggests that stress disrupts hippocampal iron homeostasis [6], with dysregulated iron metabolism exacerbating oxidative damage [7]. Ferroptosis, an iron-dependent form of programmed cell death driven by lipid peroxidation, has recently emerged as a pivotal mechanism in neuropsychiatric disorders [8]. While stress alters hippocampal iron metabolism and oxidative stress markers [6, 9], its causal role in hippocampal neuronal ferroptosis and subsequent depression requires definitive validation.

Emerging evidence indicates that the gut microbiota modulates host cellular iron metabolism regulation [10]. Therapeutic modulation of the gut microbial composition has been shown to ameliorate iron dyshomeostasis in Parkinson’s disease mouse models and cerebral ischaemia–reperfusion injury models [11, 12], with the bidirectional gut-brain axis playing a pivotal regulatory role. Stress induces gut microbiota alterations, whereas restoring microbial balance ameliorates stress-induced neural damage [13]. However, whether stress drives hippocampal neuronal ferroptosis and depression through microbiota dysbiosis remains unknown.

To address these gaps, we employed time-course restraint stress models combined with the ferroptosis inhibitors ferrostatin-1 (Fer-1) and deferoxamine (DFO) to validate the occurrence of ferroptosis in hippocampal neurons and its contribution to depressive-like behavioral alterations. Integrated approaches, including fecal microbiota transplantation (FMT), 16S rDNA sequencing, and short-chain fatty acid (SCFA) metabolomics, were utilized to identify pivotal gut microbial taxa and metabolites while delineating their molecular mechanisms. Our findings elucidate the stress-gut microbiota-metabolism interplay and propose novel therapeutic strategies against depression through targeted modulation of the gut microbiota or ferroptosis pathways.

## Materials and methods

### Experimental animals

Male SPF-grade C57BL/6 J mice (6–7 weeks old, 20–23 g) were purchased from Beijing Vital River Laboratory Animal Technology Co., Ltd. The mice were housed under controlled conditions: a temperature of 20–22 °C, humidity of 45 ± 5%, and a 12-h light/dark cycle. Sterilized food and water were provided ad libitum. Following a 7-day acclimatization period in the new environment, the experiments were initiated. All procedures strictly adhered to the ethical guidelines of the Laboratory Animal Ethics Committee of Hebei Medical University. The experimental protocol was approved by the Animal Management and Ethics Committee (IACUC-Hebmu-2023011).

### Experimental design

#### Ferroptosis inhibitor model

A restraint stress model was used to evaluate different durations of stress exposure. On the basis of our research team’s previous experience and relevant literature, we selected 3 days for acute stress [14, 15] and 7 days for chronic stress [16, 17]. The mice in the restraint stress groups (RS-3 d and RS-7 d, n = 6) were confined to cylindrical polypropylene tubes (12 cm in length, 2.4 cm in diameter) equipped with ventilation holes on both sides and adjustable stoppers at each end. The stoppers were secured to prevent the mice from moving freely or turning around. Restraint stress was applied for 8 h daily over 3 or 7 consecutive days. The control group mice (C-3 d and C-7 d, n = 6) were deprived of food and water during the same period each day but otherwise maintained under normal conditions.

**Fig. 1:**
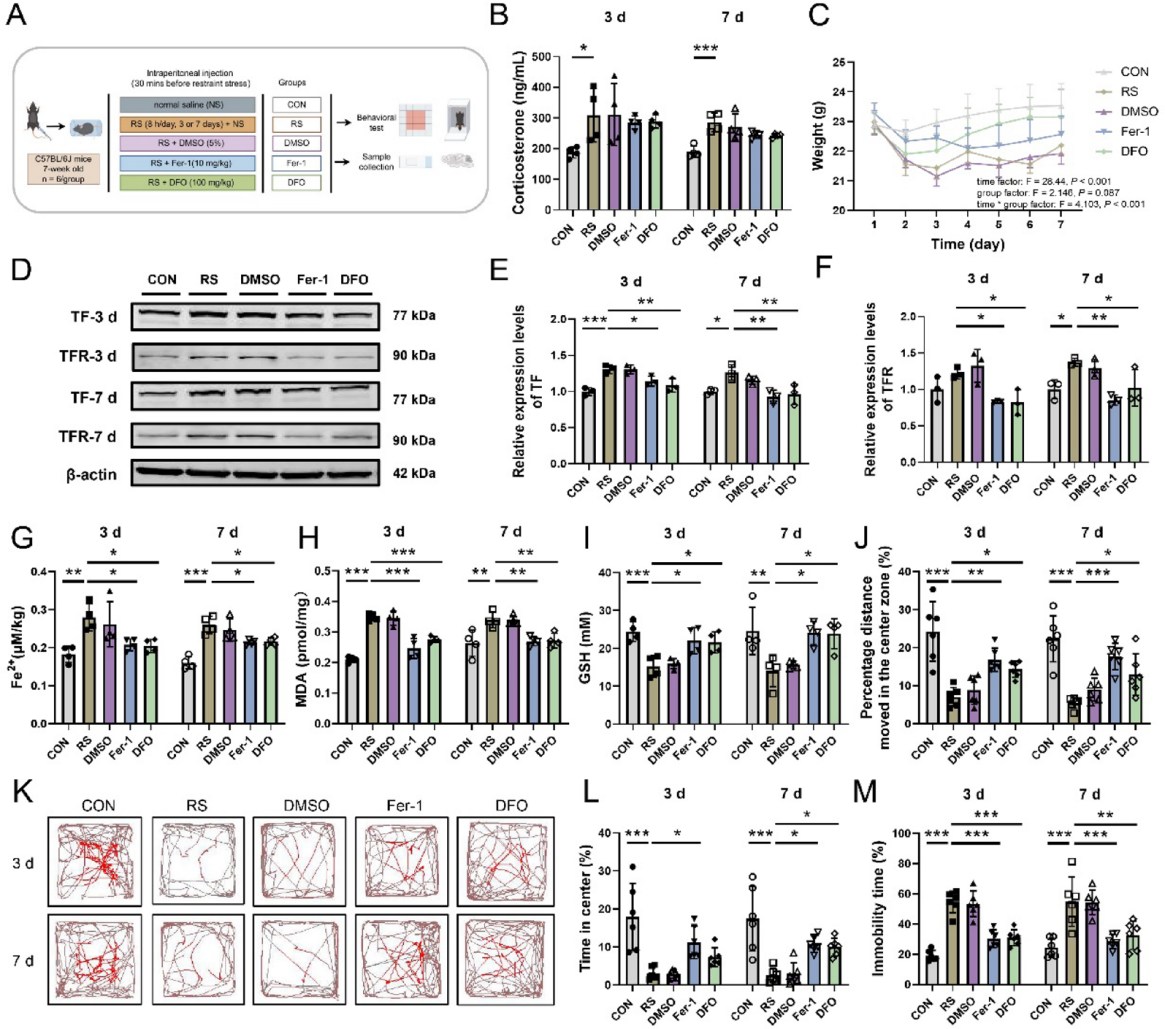
Stress-induced ferroptosis in hippocampal neurons contributes to depressive-like behavior in mice. **A** Experimental flowchart for the administration of ferroptosis inhibitors. This diagram was created via Figdraw, ID: IURTP62664. **B** Serum corticosterone levels in each group of mice, n = 4. **C** Body weight data for each group of mice. The data are presented as the means ± standard errors of the means, with statistical significance determined by two-way repeated-measures ANOVA. Days 1–3, n = 12; Days 4–7, n = 6. **D**–**F** Relative protein expression levels of TF (**E**) and TFR (**F**) in hippocampal tissue from each group, n = 3. **G**–**I** Levels of Fe.^2+^ (**G**), MDA (**H**), and GSH (**I**) in hippocampal tissue from each group, n = 4. **J**–**L** Representative movement trajectory plots (**K**), along with the percentage of distance travelled in the center zone (**J**) and the percentage of time spent in the center zone (**L**) in the open field test, n = 6. **M** Percentage of immobility time in the tail suspension test, n = 6. The data are presented as the means ± SDs, with statistical significance assessed by one-way ANOVA. *P < 0.05, **P < 0.01, ***P < 0.001

To assess the role of ferroptosis, the mice were treated with the ferroptosis inhibitors Fer-1 (17,729–10; Cayman Chemical, MI, USA) or DFO (14595-1; Cayman Chemical, MI, USA). Stock solutions of Fer-1 (10 mg/mL) and DFO (100 mg/mL) were prepared using DMSO (5%) as the solvent. These solutions were further diluted with sterile saline to final concentrations of 0.5 mg/mL (Fer-1) and 5 mg/mL (DFO) for intraperitoneal injection 30 min prior to each restraint session. For each stress duration, the mice were divided into five groups: the CON group (deprived of food and water during the same period each day, saline, n = 6), the RS group (restrained stress for 3 or 7 days, saline, n = 6), the DMSO group (restrained stress for 3 or 7 days, 5% DMSO, 0.2 mL, n = 6), the Fer-1 group (restrained stress for 3 or 7 days, Fer-1, 10 mg/kg, n = 6) [18], and the DFO group (restrained stress for 3 or 7 days, DFO, 100 mg/kg, n = 6) [19]. The experimental workflow is illustrated in Fig. 1A.

#### Fecal microbiota transplantation experiment

**Fig. 2:**
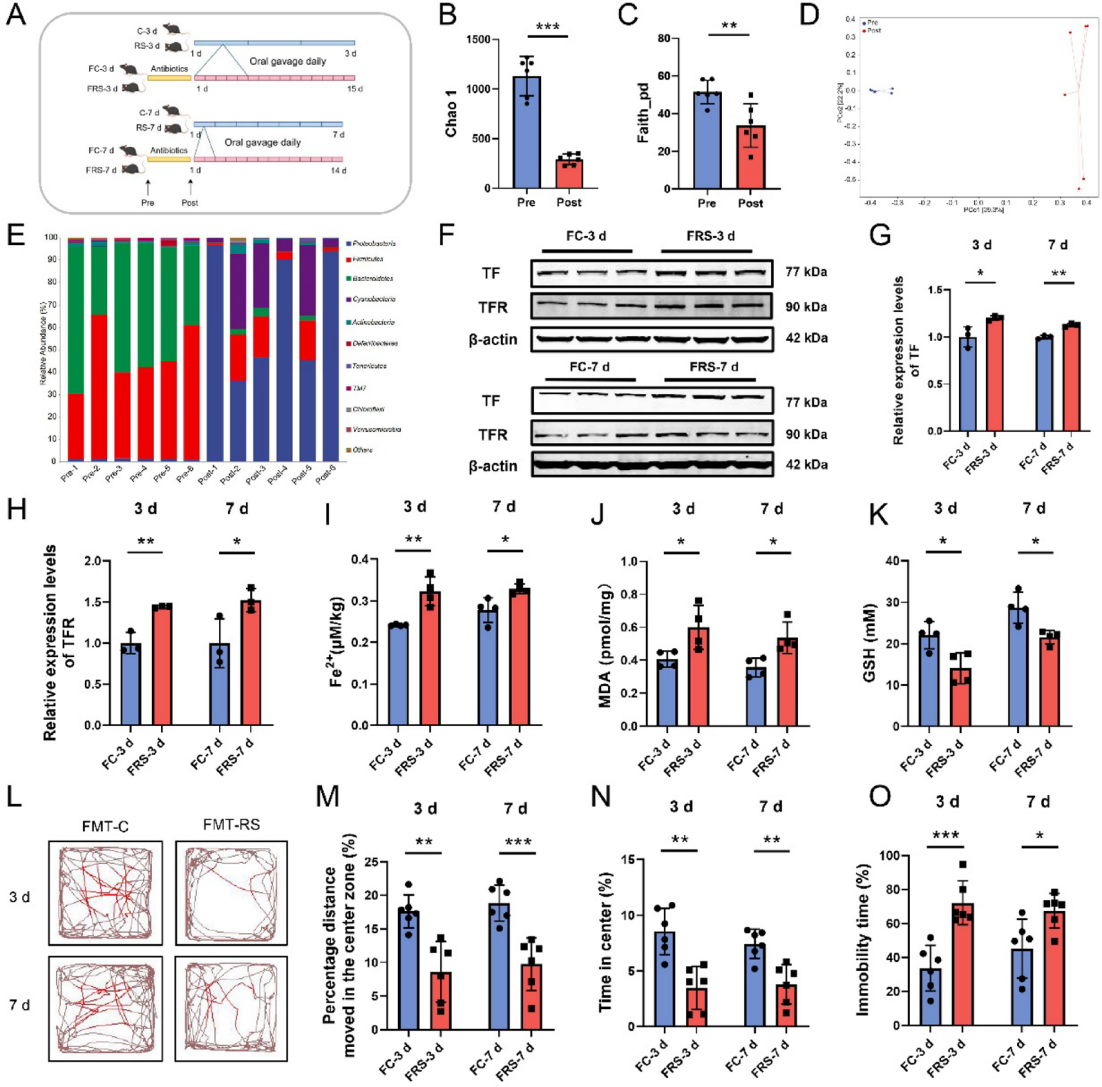
The gut microbiota is involved in the ferroptosis of hippocampal neurons in stressed mice. **A** Experimental flowchart for FMT, created via FigDraw, ID: SSUWY8887b. **B**–**C** Chao1 index (**B**) and Faith-pd index (**C**) of the gut microbiota of the mice before and after antibiotic treatment. **D** β-diversity of the gut microbiota in mice before and after antibiotic treatment (PCoA based on Bray–Curtis distances). **E** Relative abundance of the gut microbiota at the phylum level before and after antibiotic treatment, n = 6. **F**–**H** Relative protein expression levels of TF (**G**) and TFR (**H**) in hippocampal tissue from each group in the FMT experiment, n = 3. **I**–**K** Levels of Fe^2+^ (**I**), MDA (**J**), and GSH (**K**) in hippocampal tissue from each group in the FMT experiment, n = 4. **L**–**N** Representative movement trajectory plots (**L**), percentage of distance travelled in the center zone (**M**), and percentage of time spent in the center zone (**N**) during the open field test for each group in the FMT experiment, n = 6. **O** Percentage of immobility time in the tail suspension test for each group in the FMT experiment, n = 6. The data are presented as the means ± SDs. For normally distributed data, Student’s t test was used; for nonnormally distributed data, the Mann–Whitney U test was used. *P < 0.05, **P < 0.01, ***P < 0.001. Pre: before antibiotic treatment; Post: after antibiotic treatment; FC-3 d and FC-7 d: fecal transplantation from C-3 d and C-7 d mice; FRS-3 d and FRS-7 d: fecal transplantation from RS-3 d and RS-7 d mice

Twenty-four male C57BL/6 J mice (5–6 weeks old) were obtained from the Beijing Vital River Laboratory Animal Center for the FMT experiment. The mice were randomly divided into four groups as follows (n = 6): the FC-3 d group (gavaged with fecal microbiota suspension from mice in the 2.2.1 C-3 d group), the FRS-3 d group (gavaged with fecal microbiota suspension from mice in the 2.2.1 RS-3 d group), the FC-7 d group (gavaged with fecal microbiota suspension from mice in the 2.2.1 C-7 d group), and the FRS-7 d group (gavaged with fecal microbiota suspension from mice in the 2.2.1 RS-7 d group). Before FMT, all recipient mice were subjected to antibiotic treatment to deplete the gut microbiota. The antibiotic cocktail consisted of ampicillin (180 mg/kg/day), vancomycin (72 mg/kg/day), metronidazole (90 mg/kg/day), and imipenem (90 mg/kg/day). These antibiotics were administered twice daily (every 12 h) for three consecutive days, with fresh solutions prepared daily to maintain activity [20]. All antibiotics were purchased from Solarbio (Beijing, China). FMT was initiated on the fourth day, at least 24 h after the final antibiotic dose. Fresh fecal samples were collected daily from the donor groups (C-3 d, RS-3 d, C-7 d, and RS-7 d) during the 2.2.1 experiments and stored at − 80 °C in sterile 1.5 mL EP tubes. For transplantation, the fecal samples were thawed, diluted in sterile saline, and homogenized manually via sterile disposable pestles. The fecal suspensions were centrifuged at 1200 rpm for 5 min, and the supernatant was collected and administered to the recipient mice via oral gavage. FMT was performed daily for 14 or 15 days [21]. The experimental workflow is shown in Fig. 2A.

#### Butyric acid and valeric acid intervention experiments

To investigate the effects of butyric acid on ferroptosis in the hippocampal neurons of mice subjected to 3 days of restraint stress, the mice were divided into four groups (n = 6): the C3 + NS group (saline gavage), the C3 + BA group (sodium butyric acid gavage), the RS3 + NS group (3 days of restraint stress + saline gavage), and the RS3 + BA group (3 days of restraint stress + sodium butyric acid gavage). Sodium butyric acid (303,410-5G, Sigma-Aldrich, MO, USA) was administered via oral gavage at a dose of 1200 mg/kg/day for 30 min prior to restraint for three consecutive days [22].

To examine the effects of valeric acid on ferroptosis in the hippocampal neurons of the mice subjected to 7 days of restraint stress, the mice were divided into four groups (n = 6): the C7 + NS group (saline gavage), the C7 + VA group (sodium valeric acid gavage), the RS7 + NS group (7 days of restraint stress + saline gavage), and the RS7 + VA group (7 days of restraint stress + sodium valeric acid gavage). Sodium valeric acid (S194180, Aladdin, Shanghai, China) was administered via oral gavage at a dosage of 15 mg/kg/day for 30 min prior to restraint for seven consecutive days [23].

### Fecal collection and 16S rDNA (V3-V4 region) sequencing

Fecal samples were collected in sterile EP tubes from each group of mice on the final day of restraint stress modelling, before and after antibiotic treatment, and at the conclusion of FMT. All the samples were immediately frozen in liquid nitrogen and stored at − 80 °C until further analysis.

The V3-V4 hypervariable region of the 16S rDNA gene was selected for amplification via the forward primer 5′-ACTCCTACGGGAGGCAGCA-3′ and reverse primer 5′-GGACTACHVGGGTWTCTAAT-3′. The PCR products were purified, quantified, pooled in equimolar concentrations, and sequenced via the paired-end 2 × 250 bp configuration on the Illumina NovaSeq platform (Shanghai Personal Biotechnology Co., Ltd., Shanghai, China). Microbiome bioinformatics analyses were conducted via QIIME 2 (version 2019.4) following official guidelines (https://docs.qiime2.org/2019.4/tutorials/) [24]. Amplicon sequence variants (ASVs) were generated via the DADA2 plugin, and the taxonomic classification of the ASVs was performed via the Greengenes database.

### Short-chain fatty acid metabolomics

Gas chromatography-mass spectrometry (GC–MS) was utilized to measure the concentrations of seven common SCFAs: acetic acid, propionic acid, isobutyric acid, butyric acid, isovaleric acid, valeric acid, and caproic acid. Standard solutions were prepared as follows: acetic acid (64-19-7, Sigma, MO, USA), propionic acid (79-09-4, Sigma, MO, USA), butyric acid (107-92-6, Sigma, MO, USA), isobutyric acid (79-31-2, Sigma, MO, USA), valeric acid (109-52-4, Sigma, MO, USA), and isovaleric acid (503-74-2, Sigma, MO, USA) were mixed with water to create stock solutions at a concentration of 100 mg/mL. These stock solutions were then diluted with water to prepare a series of working solutions. Caproic acid (142-62-1, Sigma, MO, USA) was prepared in diethyl ether (60-29-7, Greagent, Shanghai, China) to produce a 100 mg/mL stock solution, which was further diluted with diethyl ether to generate a series of working solutions. Stock solutions were stored at − 20 °C, and all working solutions were freshly prepared prior to use.

For sample analysis, approximately 50 μL of 15% phosphoric acid, 10 μL of the internal standard solution (isohexanoic acid, 646–07-1, Sigma, MO, USA), and 140 μL of diethyl ether were added to a measured amount of sample in a 2 mL centrifuge tube. The mixture was homogenized for 1 min and centrifuged at 12,000 rpm for 10 min at 4 °C. The supernatant was collected and analysed via GC–MS at Shanghai Personal Biotechnology Co., Ltd. (Shanghai, China).

### Behavioral assessments

#### Open field test

Prior to testing, the mice were acclimated to a quiet laboratory environment for 1 h. Each mouse was then placed at the center of the open field, and its movement was recorded for 5 min via the overhead camera. The key parameters analysed included the percentage of distance travelled and the percentage of time spent in the central area of the open field. After each test, the apparatus was cleaned to remove excrement and wiped with 75% ethanol. Testing resumed only after the ethanol had fully evaporated.

#### Tail suspension test

Prior to testing, the mice were acclimated to the laboratory environment for 1–2 h. Each mouse was gently removed from its cage, and its tail was secured via medical adhesive tape applied 1 cm from the tip of the tail. The mouse was suspended head-down, with the tail tip positioned approximately 30 cm above the ground. Each session lasted 6 min, and the immobility time during the last 5 min was recorded and analysed. The test chamber was cleaned after each session to remove feces and urine.

### HE staining and special staining

The mice were anaesthetized via intraperitoneal injection of 2% sodium pentobarbital (0.1 mL/20 g) and immediately euthanized. Brain and colon tissues were collected and fixed in 10% neutral formalin. The tissues were then dehydrated in graded ethanol, cleared in xylene, and embedded in paraffin. The tissue sections were baked in a 60 °C oven and stored at 4 °C until further use. For hematoxylin and eosin (HE) staining, the tissue sections were deparaffinized and rehydrated, followed by staining with hematoxylin solution, differentiation with hydrochloric acid alcohol, eosin staining, rinsing with tap water, dehydration, clearing, and mounting. The sections were then rinsed with running water, dehydrated, cleared, and mounted. Alcian blue staining was performed via an Alcian blue staining kit (G1560, Solarbio, Beijing, China) following the manufacturer’s instructions. The stained sections were visualized and imaged using an Olympus DP80 microscope (Olympus, Tokyo, Japan). The microscopy images were analysed and quantified via ImageJ software (version 1.50b; National Institutes of Health, Maryland, USA).

### Western blotting

Hippocampal and colon tissues were homogenized on ice in RIPA lysis buffer supplemented with PMSF (R0010, Solarbio, Beijing, China). The homogenates were subsequently centrifuged at 12,000 rpm for 10 min at 4 °C, after which the supernatants were collected. Equal amounts of protein were separated by SDS-PAGE and transferred onto PVDF membranes. The membranes were blocked with Y-Tec ready-to-use blocking buffer (YWB0501S, Yoche, Shanghai, China) for 5 min and incubated with the following primary antibodies: transferrin (TF, 1:1000, A1448, ABclonal, Wuhan, China), transferrin receptor (TFR, 1:1000, 13–6800, Invitrogen, CA, USA), Claudin-5 (1:2000, AF5216, Affinity Biosciences, Jiangsu, China), Occludin (1:2000, ET1701-76, Huabio, Hangzhou, China), G protein-coupled receptor 41 (GPR41, 1:1000, Huabio, Hangzhou, China), Ras homologue family member A (RhoA, 1:1000, 2117S, CST, MA, US), Rho-associated coiled-coil containing protein kinase 1 (Rock1, 1:1000, ET1609-53, Huabio, Hangzhou, China) and β-actin (1:2000, 66,009–1-Ig, Proteintech, Wuhan, China). The secondary antibodies used included goat anti-rabbit IgG (1:2000, A23920, Abbkine, Beijing, China) and goat anti-mouse IgG (1:2000, A23710, Abbkine, Beijing, China). The membranes were visualized via an Odyssey imaging system (Odyssey V3.0, LI-COR Biosciences, Nebraska, USA), and the data were analysed and quantified via ImageJ software.

### Real-time quantitative PCR (RT-qPCR)

Total RNA was extracted from hippocampal tissue via TRIzol reagent (15596018CN; Thermo Fisher Scientific, MA, USA) according to the manufacturer’s protocol. Complementary DNA (cDNA) was synthesized via a cDNA synthesis kit (A2791; Promega, Madison, WI, USA). RT-qPCR was performed via a qPCR kit (A6001, Promega, Madison, WI, USA). Each sample was analysed in triplicate, with GAPDH used as the internal reference gene for normalization. The sequences of primers used for RT-qPCR are provided in Table 1.

**Table 1:** Primers for RT-qPCR

Gene	Sequence
GAPDH(F)	GGTGAAGGTCGGTGTGAACG
GAPDH(R)	CTCGCTCCTGGAAGATGGTG
IL-1β(F)	ATCTCGCAGCACATCA
IL-1β(R)	CCAGCAGGTTATCATCATCATCC
IL-6(F)	CTTGGGACTGATGCTGGTGAC
IL-6(R)	TTCTCATTTCCACGATTTCCCA
IL-4(F)	ACAGGAGAAGGGACGCCAT
IL-4(R)	GAAGCCCTACAGACGAGCTCA
IL-10(F)	GCCAGAGCCACATGCTCCTA
IL-10(R)	GATAAGGCTTGGCAACCCAAGTAA

### Enzyme-linked immunosorbent assay (ELISA)

ELISA kits were used to measure the levels of corticosterone (E-OSEL-M0001, Elabscience, Wuhan, China), IL-1β (SEA563Mu, CLOUD-CLONE, Wuhan, China), IL-6 (SEA079Mu, CLOUD-CLONE, Wuhan, China), IL-4 (SEA077Mu, CLOUD-CLONE, Wuhan, China), and IL-10 (SEA056Mu, CLOUD-CLONE, Wuhan, China) in mouse serum, as well as butyric acid (CEO777Ge, CLOUD-CLONE, Wuhan, China) in hippocampal tissue. All the assays were conducted in accordance with the manufacturer’s instructions.

### Evaluation of glutathione (GSH), malondialdehyde (MDA), and Fe^2^⁺ levels

The levels of GSH and oxidized glutathione (GSSG) were measured via a GSH and GSSG detection kit (S0053, Beyotime, Shanghai, China). The MDA content was quantified via an MDA detection kit (S0131S; Beyotime, Shanghai, China), and the Fe^2^⁺ level was assessed via a tissue iron content detection kit (AKIC001M; Boxbio, Beijing, China). All procedures followed the manufacturer’s instructions.

### Statistical analysis

Statistical analyses were performed via GraphPad Prism 8 software (version 8.0.2; GraphPad Software, San Diego, USA). The data are presented as the means ± standard deviations (SDs). For normally distributed data, Student’s t test was used for comparisons between two groups, whereas one-way analysis of variance (ANOVA) was employed for multiple group comparisons. Nonnormally distributed data were assessed via the Mann–Whitney U test for two-group comparisons and the Kruskal–Wallis test for multiple-group comparisons. Two-way repeated-measures ANOVA was used to assess the statistical significance of the differences in body weight across the groups. *P* < 0.05 was considered statistically significant. For the omics data in the article, particularly the metabolomics data identifying core metabolites, we used PASS 15 software to calculate the statistical power for the expression differences of butyric acid and valeric acid between the control group and the acute/chronic stress groups. The effect size was determined based on the mean and standard deviation of each group. With the parameters set at α = 0.05 and a sample size of 6, the calculated statistical power was 1-β > 0.90, indicating that the selected sample size was statistically meaningful. Furthermore, for SCFA metabolomics analysis, *P* values were calculated via either Student’s t test or the Mann–Whitney U test. Metabolites with *P* < 0.05 and VIP > 1 were considered statistically significant. Spearman correlation analysis was performed to explore the relationships between the gut microbiota and SCFAs. Correlations with |rho|> 0.5 and* P* < 0.05 were considered significant and are presented accordingly.

## Results

### Stress-induced ferroptosis in hippocampal neurons contributes to depression-like behavioural changes in mice

To ascertain whether stress induces ferroptosis in hippocampal neurons and fosters depression-like behaviors, we used acute (3-day) and chronic (7-day) restraint stress models in mice [14–17] and administered the ferroptosis inhibitors Fer-1 and DFO (Fig. 1A). Successful stress induction was confirmed by elevated serum corticosterone (Fig. 1B) and reduced body weight (Fig. 1C) in both stress paradigms. Stress exposure significantly upregulated the expression of hippocampal ferroptosis markers, including TF and TFR (Fig. 1D-F), Fe^2^⁺ accumulation (Fig. 1G), and MDA levels (Fig. 1H), while depleting GSH reserves (Fig. 1I). Histopathological analysis revealed increased red-stained neurons with disrupted nuclear structures in the stressed hippocampus (Fig. S1A-B), paralleled by depressive-like behaviors: reduced central locomotion and central dwelling time in the open field test (Fig. 1J–L), along with prolonged immobility in the tail suspension test (Fig. 1M). Crucially, the administration of Fer-1 and DFO significantly reversed these ferroptotic alterations (Fig. 1D–I), attenuated neuronal damage (Fig. S1A-B), and normalized behavioral deficits (Fig. 1J–M). Collectively, these findings demonstrate that restraint stress triggers hippocampal neuronal ferroptosis, which mechanistically drives the emergence of depressive-like phenotypes in mice.

### The gut microbiota critically mediates stress-induced hippocampal neuronal ferroptosis and depressive-like behaviors in mice

To determine whether alterations in the gut microbiota contribute to stress-induced ferroptosis in hippocampal neurons, we conducted FMT experiments (Fig. 2A). Antibiotic cocktail pretreatment effectively depleted gut microbial diversity (Fig. 2B–C) and restructured bacterial communities (Fig. 2D–E), establishing an antibiotic-treated mouse model. Subsequent FMT from 3-day and 7-day stress-exposed donors to antibiotic-treated recipients recapitulated hippocampal ferroptotic signatures in recipient mice (termed the FRS-3 d and FRS-7 d groups), as evidenced by upregulated TF and TFR expression (Fig. 2F–H), elevated Fe^2^⁺ and MDA levels (Fig. 2I–J) alongside depleted GSH (Fig. 2K), accompanied by increased red-stained neurons (Fig. S2A–B) and depressive-like behavioral manifestations (Fig. 2L–O). These findings collectively demonstrate that gut microbial remodelling drives stress-induced ferroptosis and associated neurobehavioral pathologies.

### SCFA-producing bacteria are key gut microbes involved in the ferroptosis of hippocampal neurons in stressed mice

**Fig. 3:**
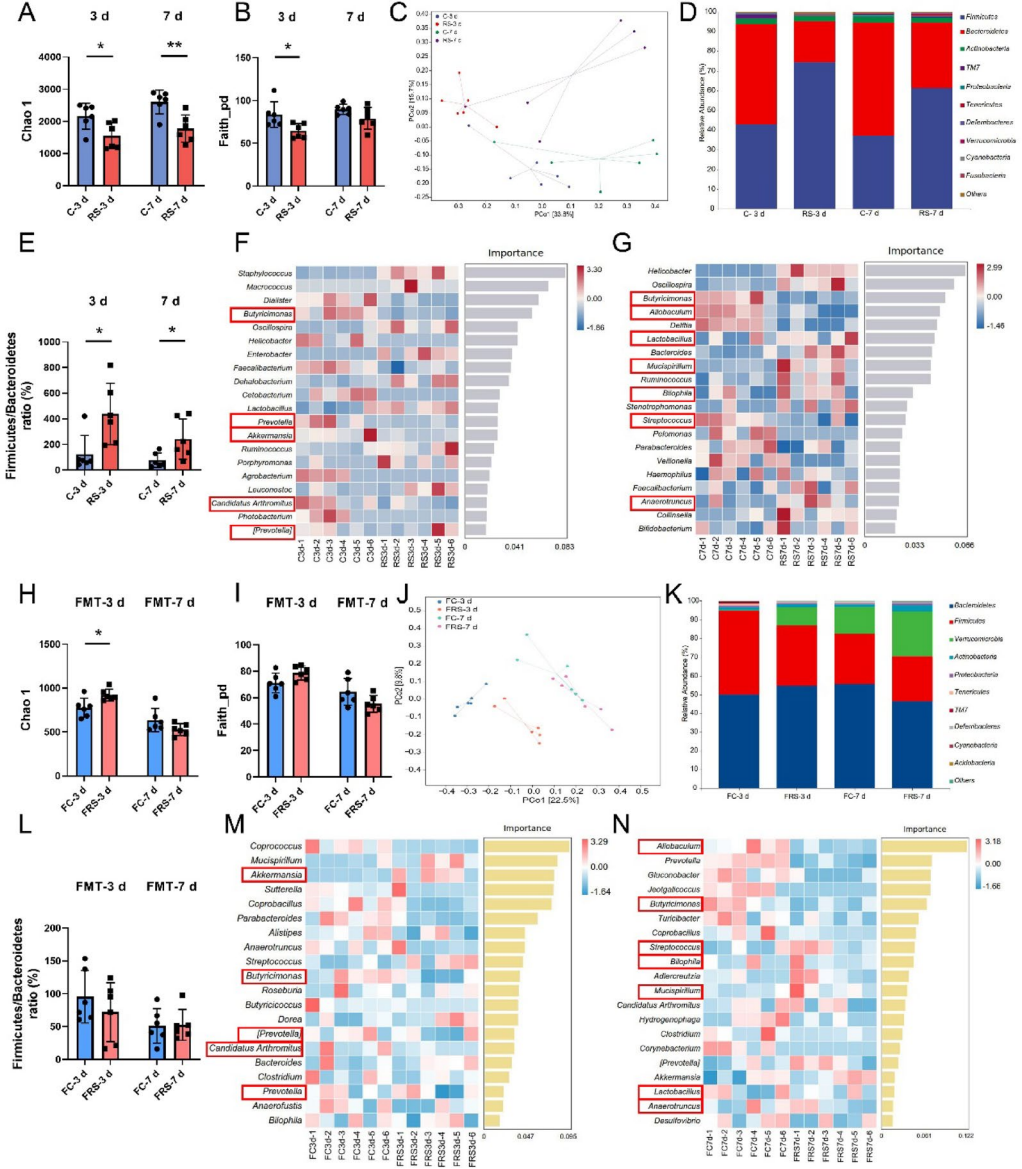
SCFA-producing bacteria are key contributors to ferroptosis in the hippocampal neurons of stressed mice. **A**–**B** Chao1 index (**A**) and Faith-pd index (**B**) of the gut microbiota in each group of mice. **C** β diversity of the gut microbiota in each group of mice, as determined via PCoA of Bray–Curtis distances. **D** Relative abundance of the gut microbiota at the phylum level in each group of mice. **E** Ratio of the relative abundances of *p_Firmicutes* and *p_Bacteroidetes* in each group of mice. **F** Random forest analysis of the gut microbiota in the C-3 d and RS-3 d groups. **G** Random forest analysis of the gut microbiota in the C-7 d and RS-7 d groups. n = 6. (H-I) Chao1 index (**H**) and Faith-pd index (**I**) of the gut microbiota in each group of mice after FMT. **J** β diversity of the gut microbiota in each group of mice after FMT, as determined via PCoA of Bray–Curtis distances. **K** Relative abundance of the gut microbiota at the phylum level in each group of mice after FMT. **L** Ratio of the relative abundances of *p_Firmicutes* and *p_Bacteroidetes* in each group of mice after FMT. **M** Random forest analysis of the gut microbiota in the FC-3 d and FRS-3 d groups. **N** Random forest analysis of the gut microbiota in the FC-7 d and FRS-7 d groups. n = 6. The red boxes highlight the gut microbiota that are altered in both stressed and fecal microbiota-transplanted mice. The data are expressed as the means ± SDs. Student’s t test was used for normally distributed data, whereas the Mann–Whitney U test was used for nonnormally distributed data. *P < 0.05, **P < 0.01

To identify the gut microbial taxa critically involved in stress-induced hippocampal ferroptosis, we performed 16S rDNA sequencing on fecal samples from time-course stress models and FMT recipients. Both 3-day and 7-day stress exposures significantly reduced the gut microbial α diversity (Fig. 3A–B) and restructured the community composition (Fig. 3C). At the phylum level, stressed mice exhibited dominance of *p_Firmicutes* and *p_Bacteroidetes,* with elevated *Firmicutes/Bacteroidetes* (F/B) ratios (Fig. 3D–E). FMT recipients displayed distinct microbial profiles (Fig. 3J), showing a restored predominance of *p_Firmicutes* and *p_Bacteroidetes* (Fig. 3K). However, the α diversity indices (Fig. 3H–I) and F/B ratios (Fig. 3L) in the FMT groups showed nonsignificant intergroup variations.

**Fig. 4:**
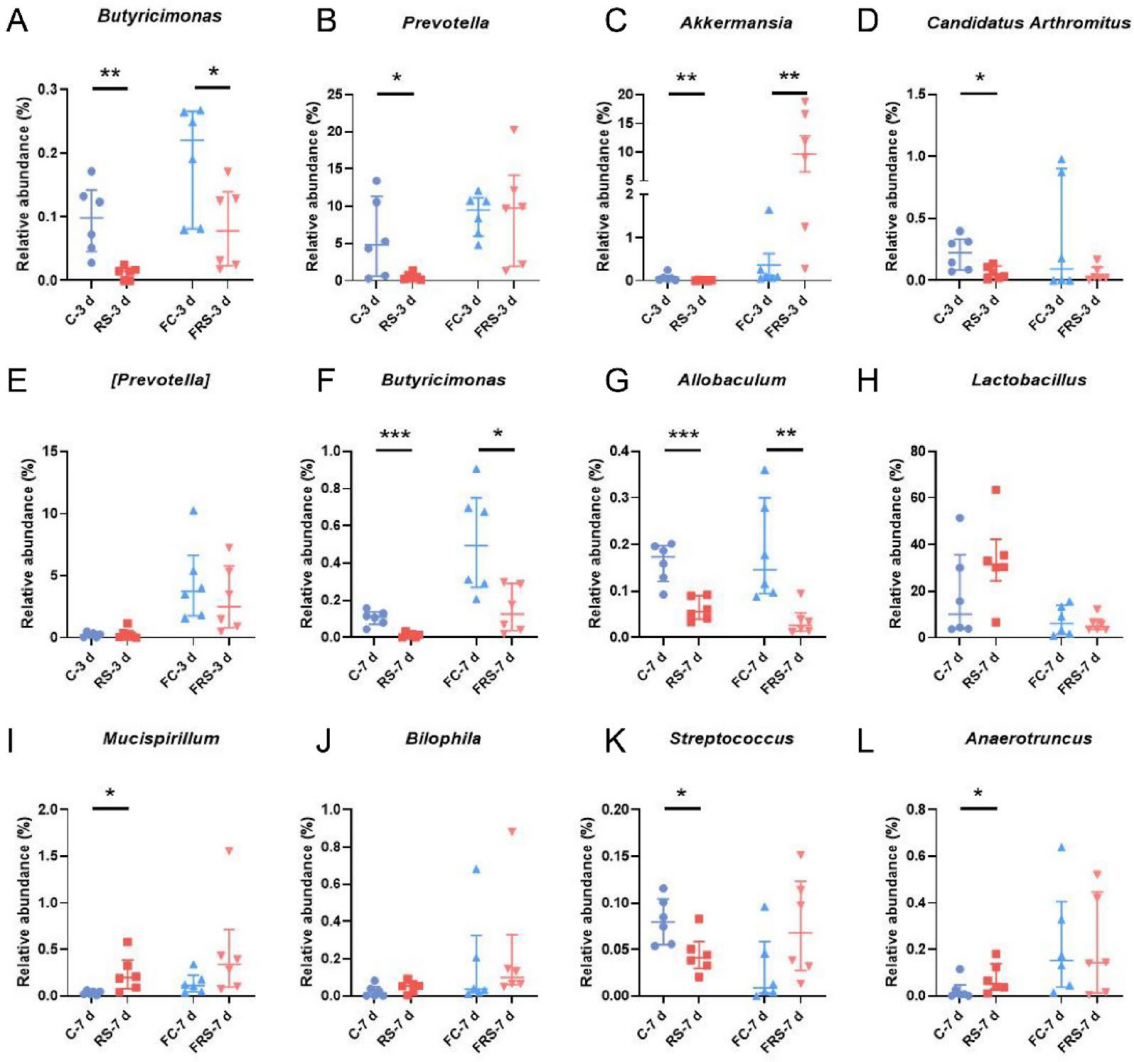
The gut microbiota involved in ferroptosis of hippocampal neurons in stressed mice at different time points. **A**–**E** The results from Fig. 3F and Fig. 3M show the relative abundances of *g_Butyricimonas* (**A**), *g_Prevotella* (**B**), *g_Akkermansia* (**C**), *g_Candidatus Arthromitus* (**D**), and *g_[Prevotella]* (**E**) in the guts of the mice in the 3-day stress model and FMT groups. **F**–**L** The results from Fig. 3G and Fig. 3N show the relative abundances of *g_Butyricimonas* (**F**), *g_Allobaculum* (**G**), *g_Lactobacillus* (**H**), *g_Mucispirillum* (**I**), *g_Bilophila* (**J**), *g_Streptococcus* (**K**), and *g_Anaerotrumcus* (**L**) in the guts of the 7-day stress model and FMT mice. n = 6. Data are presented as medians with interquartile ranges. Student’s t test was used for normally distributed data, and the Mann–Whitney U test was used for nonnormally distributed data. *P < 0.05, **P < 0.01, ***P < 0.001

Following microbial community profiling, random forest analysis was employed to prioritize the ferroptosis-related gut microbiota (Fig. 3F-G, M–N). Our focus was on genera that were consistently altered in both stress-exposed and FMT recipient mice. This analysis revealed shared microbial perturbations through comparative analyses between stress-exposed groups and their FMT recipients: *g_Butyricimonas*, *g_Prevotella*, *g_Akkermansia*, *g_Candidatus Arthromitus*, and *g_[Prevotella]* in 3 d models (Fig. 4A–E) and *g_Butyricimonas*, *g_Allobaculum*, *g_Lactobacillus*, *g_Mucispirillum*, *g_Bilophila*, *g_Streptococcus*, and *g_Anaerotruncus* in 7 d models (Fig. 4F–L). Notably, all genera except *g_Candidatus Arthromitus* and *g_Bilophila* are established SCFA producers [25–30], defined as organic fatty acids with fewer than six carbon atoms derived from microbial fermentation of undigested dietary fibres. Quantitative analysis confirmed significant alterations in *g_Butyricimonas* (3 d: Fig. 4A; 7 d: Fig. 4F), *g_Akkermansia* (Fig. 4C), and *g_Allobaculum* (Fig. 4G) across both the stress and FMT groups. Moreover, *g_Prevotella* (Fig. 4B), *g_Candidatus Arthromitus* (Fig. 4D), *g_Mucispirillum* (Fig. 4I), *g_Streptococcus* (Fig. 4K), and *g_*A*naerotruncus* (Fig. 4L) presented marked differences in stress models, with less pronounced variations between FMT subgroups. These findings highlight stress-induced dysregulation of SCFA-producing taxa as a potential driver of hippocampal ferroptosis.

### Butyric acid and valeric acid are key SCFAs involved in the ferroptosis of hippocampal neurons in stressed mice

**Fig. 5:**
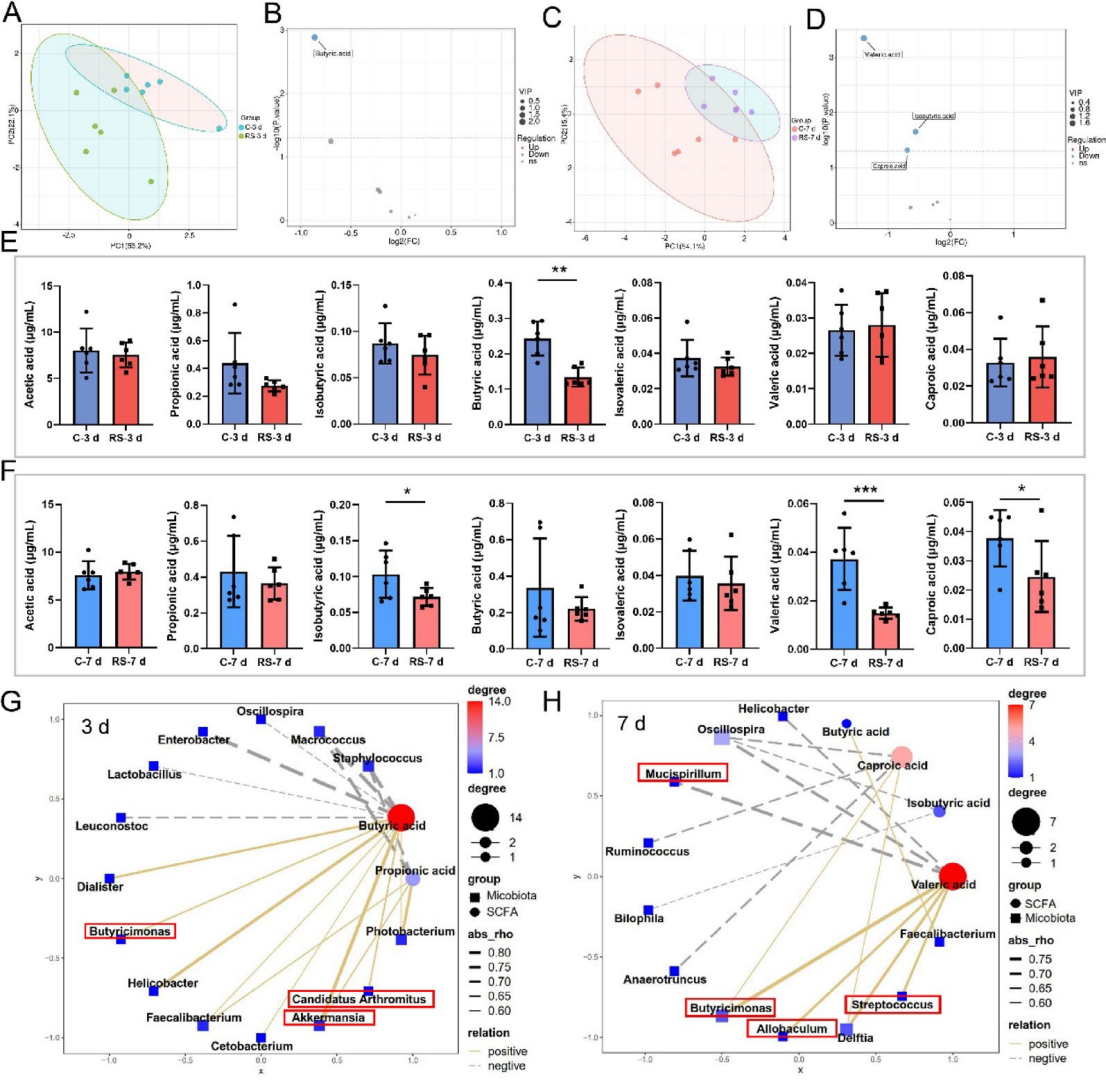
Butyric acid and valeric acid are key SCFAs linked to hippocampal ferroptosis under stress. **A** PLS-DA analysis of SCFAs in the serum of the C-3 d and RS-3 d groups of mice. **B** Volcano plot of differentially abundant metabolites in the serum of the C-3 d and RS-3 d groups of mice, with VIP > 1 and *P* < 0.05 used as the selection criteria. The blue dots represent downregulated differentially abundant metabolites, whereas the gray dots represent metabolites detected but not meeting the filtering parameters. n = 6. **C** PLS-DA analysis of SCFAs in the serum of the C-7 d and RS-7 d groups of mice. **D** Volcano plot of differentially abundant metabolites in the serum of the C-7 d and RS-7 d groups of mice, with VIP > 1 and *P* < 0.05 used as the selection criteria. n = 6. **E** Bar graph showing the concentrations of seven SCFAs in the serum of the C-3 d and RS-3 d groups of mice. **F** Bar graph showing the concentrations of seven SCFAs in the serum of the C-7 d and RS-7 d groups of mice. The data are presented as the means ± SDs. Statistical significance between two groups was determined via Student’s t test: *P < 0.05, **P < 0.01, ***P < 0.001. (**G**–**H**) Correlation network between the top 20 gut microbiota selected by random forest and the seven SCFAs in the 3-day and 7-day stress model groups. The redder the color and the larger the circle are, the stronger the correlation. n = 6

The stress-induced disruption of SCFA-producing gut bacteria prompted us to delve into the specific SCFAs implicated in hippocampal neuron ferroptosis in mice under stress at various time intervals. By employing SCFA metabolomics, we quantified the serum concentrations of seven prevalent SCFAs and revealed significant disparities between the stressed and control mice at both the 3-day and 7-day time points (Fig. 5A, C). In the 3-day stress group, a marked reduction in the serum concentration of butyric acid was observed (Fig. 5B, E). In the 7-day stress group, the levels of isobutyric acid, valeric acid, and caproic acid significantly decreased (Fig. 5D, F). To further elucidate the interplay between the gut microbiota and SCFAs, we conducted a correlation analysis between the top 20 gut microbial taxa identified through random forest analysis (Fig. 3F–G) and the serum concentrations of the seven SCFAs (Fig. 5E–F). In the 3-day stress group, butyric acid emerged as the metabolite most robustly linked to the gut microbiota, demonstrating significant positive correlations with the abundances of *g_Butyricimonas, g_Akkermansia*, and *g_Candidatus Arthromitus* (Fig. 5G). In the 7-day stress group, valeric acid was the pivotal SCFA, with a positive correlation with the abundances of *g_Butyricimonas, g_Allobaculum*, and *g_Streptococcus* but a negative correlation with *g_Mucispirillum* (Fig. 5H). These findings underscore butyric acid and valeric acid as pivotal SCFAs that may be instrumental in driving stress-induced ferroptosis of hippocampal neurons in mice.

### Butyric acid and valeric acid attenuate stress-induced hippocampal neuronal ferroptosis and depressive-like phenotypes

**Fig. 6:**
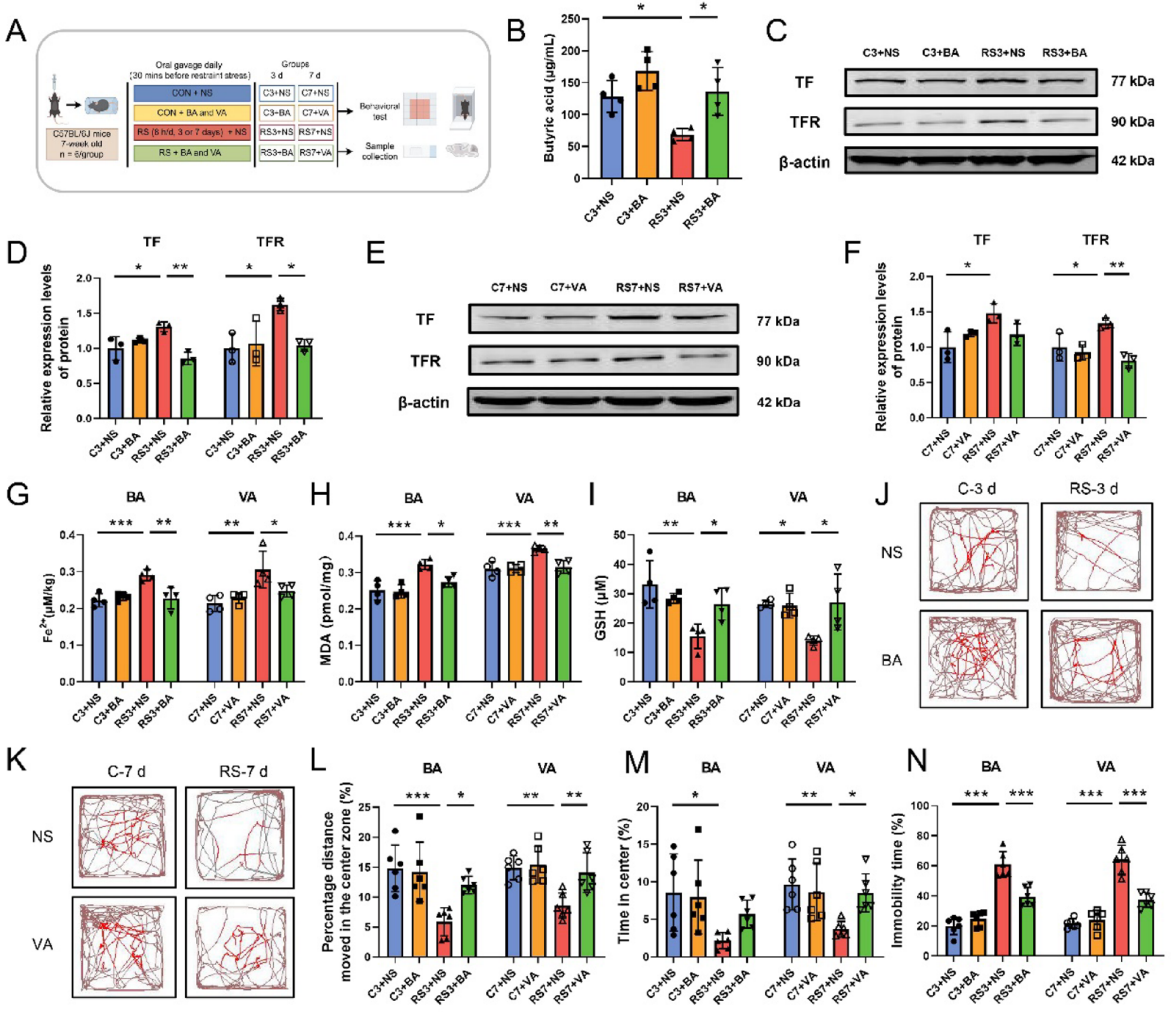
Butyric acid and valeric acid attenuate hippocampal ferroptosis and depressive behaviors in stressed mice. **A** Experimental workflow for butyric acid and valeric acid intervention, created via FigDraw, ID: UTRPUeafee. BA: butyric acid, VA: valeric acid. **B** Butyric acid levels in the hippocampal tissues of each group of mice after butyric acid intervention, n = 4. **C**–**D** Relative protein expression of TF and TFR in the hippocampal tissues of each group of mice after butyric acid intervention; n = 3. **E**–**F** Relative protein expression of TF and the TFR in the hippocampal tissues of each group of mice after valeric acid intervention; n = 3. **G**–**I** Fe.^2^⁺ (**G**), MDA (**H**), and GSH (**I**) levels in the hippocampal tissues of each group of mice after butyric acid or valeric acid intervention, n = 4. **J**–**K** Representative movement trajectories of each group of mice in the open field test. **L**–**M** Percentage of distance travelled in the center zone (**L**) and percentage of time spent in the center zone (**M**) in the open field test. **N** Percentage of immobility time in the tail suspension test for each group of mice, n = 6. The data are expressed as the means ± SDs, with statistical significance among multiple groups determined by one-way ANOVA: *P < 0.05, **P < 0.01, ***P < 0.001

To delineate the temporal-specific roles of butyric acid and valeric acid in stress-induced hippocampal ferroptosis, we conducted targeted SCFA supplementation experiments (Fig. 6A). Quantitative analysis revealed significantly reduced hippocampal butyric acid levels in the 3-day stress models (RS3 + NS vs. C3 + NS), which were effectively normalized by butyric acid supplementation (Fig. 6B). Similarly, valeric acid administration restored hippocampal valeric acid depletion in 7-day stress models (Table 2). Stress exposure markedly upregulated ferroptotic markers across both stress durations, including elevated TF and TFR expression (Fig. 6C–F), Fe^2^⁺ accumulation (Fig. 6G), and MDA levels (Fig. 6H), coupled with GSH depletion (Fig. 6I). Critically, butyric acid or valeric acid intervention reversed these pathological signatures (Fig. 6C–I) while attenuating stress-induced hippocampal neurodegeneration (Fig. S3A–B) and depressive-like behaviors (Fig. 6J–N). These findings demonstrate that gut microbiota-derived butyric acid and valeric acid differentially regulate hippocampal ferroptosis in a time-dependent manner, suggesting their therapeutic potential against stress-related neural damage.

**Table 2:** Content of valeric acid (VA) in the hippocampus

Valeric acid (μg/g)	C7 + NS	C7 + VA	RS7 + NS	RS7 + VA
Mouse 1	0.06768	0.2922	< 0.001	0.1831
Mouse 2	0.07197	0.2891	< 0.001	0.0262
Mouse 3	0.03335	0.7375	< 0.001	0.0359

### Butyric acid and valeric acid alleviate stress-induced hippocampal neuronal ferroptosis and depressive-like behaviors via neuroinflammatory suppression

**Fig. 7:**
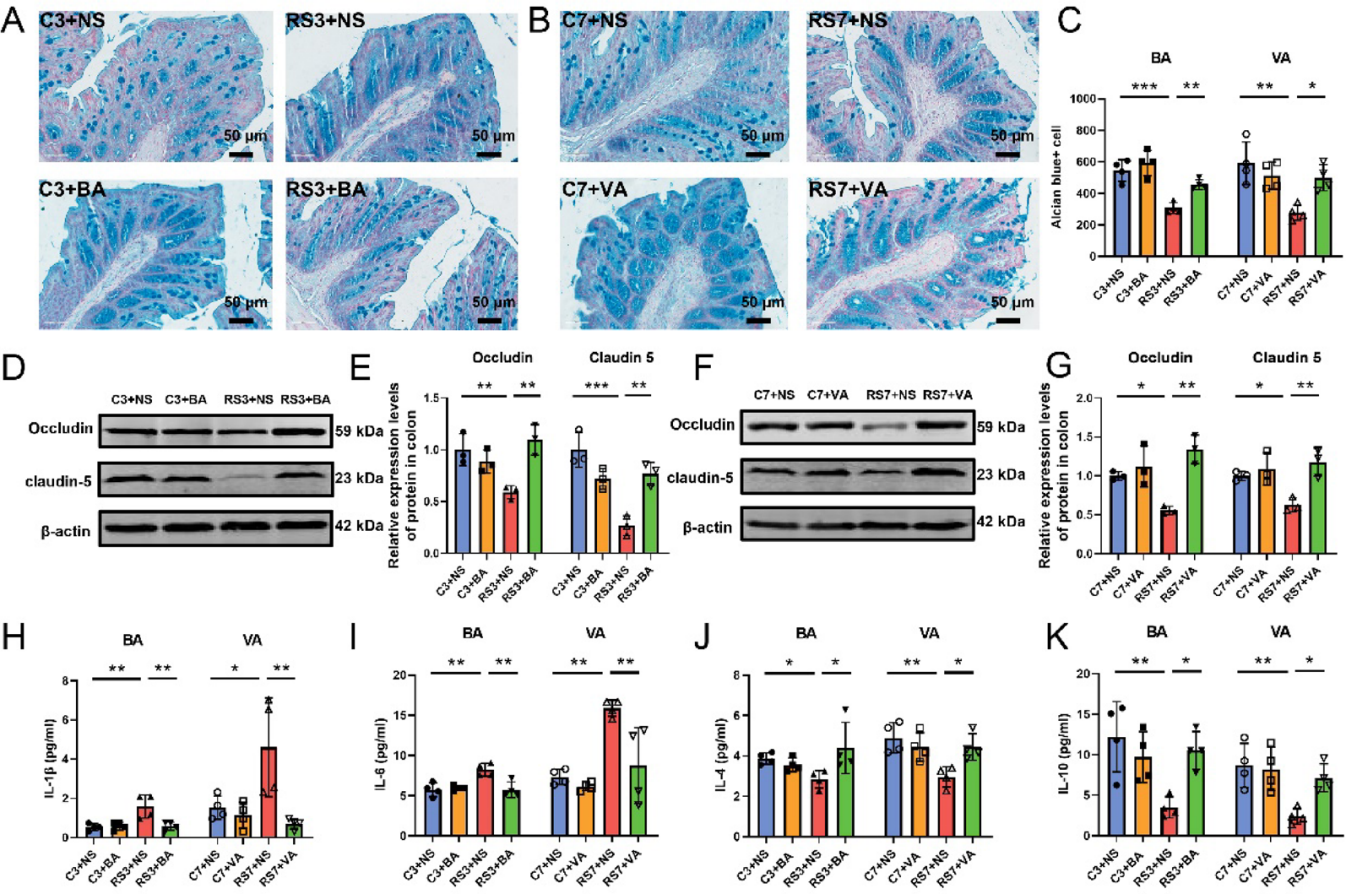
Butyric acid and valeric acid improve gut barrier integrity and systemic inflammation in stressed mice. **A**–**B** Representative images of Alcian blue staining of colon tissues from each group of mice following butyric acid or valeric acid intervention. Scale bar: 50 μm. **C** Positive cell counts from alcian blue-stained colon tissues from each group, n = 4. **D**–**E** Relative protein expression of the tight junction proteins Occludin and Claudin5 in colon tissues following butyric acid intervention, n = 3. **F**–**G** Relative protein expression of the tight junction proteins Occludin and Claudin5 in colon tissues following valeric acid intervention, n = 3. **H**–**K** Serum levels of the inflammatory cytokines IL-1β (**H**), IL-6 (**I**), IL-4 (**J**), and IL-10 (**K**) in each group of mice after butyric acid or valeric acid treatment, n = 4. The data are expressed as the means ± SDs, with statistical significance among multiple groups determined by one-way ANOVA: *P < 0.05, **P < 0.01, ***P < 0.001

**Fig. 8:**
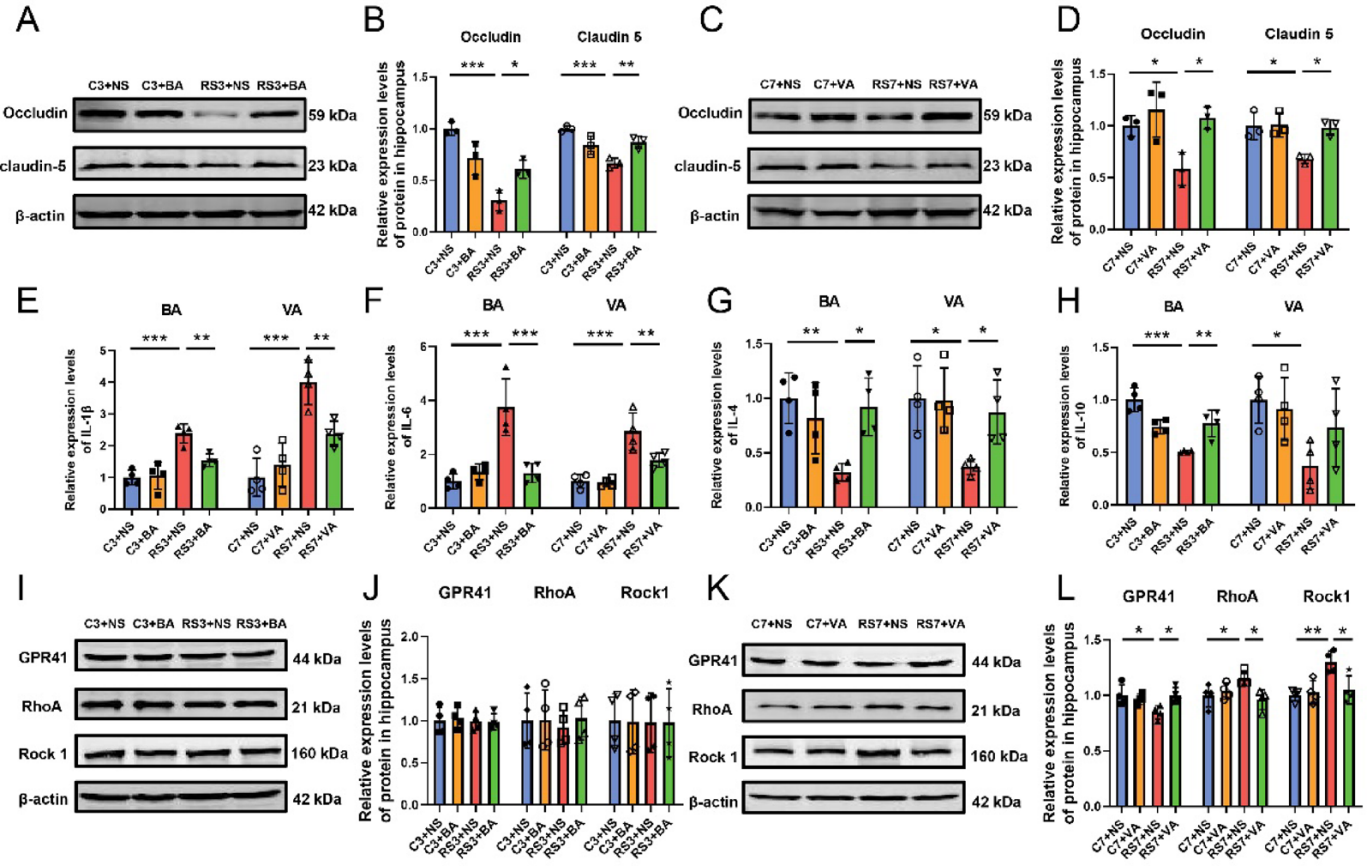
Butyric acid and valeric acid administration ameliorates hippocampal neuroinflammation in stress-exposed mice. **A**–**B** Relative protein expression of the tight junction proteins Occludin and Claudin5 in hippocampal tissues after butyric acid intervention; n = 3. **C**–**D** Relative protein expression of the tight junction proteins Occludin and Claudin5 in hippocampal tissues after valeric acid intervention, n = 3. **E**–**H** mRNA expression levels of the inflammatory cytokines IL-1β (**E**), IL-6 (**F**), IL-4 (**G**), and IL-10 (**H**) in hippocampal tissues after butyric acid or valeric acid intervention, n = 4. **I**–**J** Relative protein expression levels of GPR41, RhoA, and Rock1 in hippocampal tissues across the experimental groups following butyric acid administration, n = 4. **K**–**L** Relative protein expression levels of GPR41, RhoA, and Rock1 in hippocampal tissues across the experimental groups following valeric acid administration, n = 4. The data are presented as the means ± SDs, with statistical significance determined by one-way ANOVA: *P < 0.05, **P < 0.01, ***P < 0.001

Preclinical studies have demonstrated that neuroinflammation triggers oxidative stress to fuel lipid peroxidation while modulating iron metabolism regulators such as TFR, thereby increasing intracellular labile iron pools [31, 32]. To investigate whether butyric acid and valeric acid alleviate stress-induced hippocampal ferroptosis through anti-inflammatory mechanisms, we first evaluated intestinal barrier integrity. Alcian blue staining revealed significant reductions in the number of colonic goblet cells across the stress models (Fig. 7A–C), accompanied by decreased expression of the tight junction proteins Occludin and Claudin-5 (Fig. 7D–G), confirming stress-induced disruption of the gut barrier. Such barrier disruption enables the translocation of harmful microbial metabolites into systemic circulation, triggering proinflammatory cascades [33]. Consistent with this mechanism, stressed mice presented significantly elevated serum levels of the proinflammatory cytokines IL-1β and IL-6 (Fig. 7H–I) and reduced levels of the anti-inflammatory mediators IL-4 and IL-10 (Fig. 7J–K). Concurrently, increased blood–brain barrier permeability (Fig. 8A–D) and hippocampal cytokine dysregulation, characterized by upregulated IL-1β and IL-6 mRNA and downregulated IL-4 and IL-10 mRNA (Fig. 8E–H), were observed. Crucially, butyric acid and valeric acid supplementation restored intestinal and blood–brain barrier integrity (Fig. 7A–G, 8A–D), normalized both systemic and hippocampal cytokine profiles (Fig. 7H–K, 8E–H), and concurrently ameliorated ferroptotic molecular signatures (Fig. 6C–I) and depressive-like behaviors (Fig. 6J–N). These findings collectively demonstrate that butyric acid and valeric acid confer neuroprotection by repairing the intestinal barrier and blood–brain barrier, thereby alleviating neuroinflammation, which in turn mitigates hippocampal neuronal ferroptosis.

In addition to peripheral inflammatory infiltration through the compromised blood–brain barrier [34], SCFAs such as butyric acid and valeric acid traverse the blood–brain barrier via monocarboxylate transporters to directly modulate neuroinflammatory responses by engaging neuronal receptors such as GPR41, which regulates downstream signalling pathways [23, 35, 36]. RhoA, a member of the Rho GTPase family, regulates cytoskeletal dynamics and cellular migration through its downstream effector Rock. Activation of the RhoA/Rock signalling pathway promotes proinflammatory cytokine production, driving neuroinflammatory cascades [37, 38]. Given that GPR41 activation suppresses this pathway [23], to investigate the molecular mechanisms by which butyric acid and valeric acid improve neuroinflammation and alleviate hippocampal neuronal ferroptosis, we measured the protein expression of GPR41, RhoA, and Rock1 in the hippocampus of mice. The results revealed that 7 days of chronic stress significantly downregulated GPR41 expression while activating the RhoA/Rock1 pathway. Valeric acid supplementation significantly improved this effect (Fig. 8K–L). Notably, acute 3-day stress caused no significant alterations in this pathway (Fig. 8I–J), indicating that the therapeutic effects of valeric acid are specific to chronic stress. These findings demonstrate that valeric acid alleviates chronic stress-induced ferroptosis potentially by enhancing GPR41-mediated inhibition of RhoA/Rock1 signalling, thereby reducing neuroinflammation. Interestingly, the anti-ferroptotic effects of butyric acid appear to operate through mechanisms distinct from this pathway.

## Discussion

Stress is a complex physiological, psychological, and social phenomenon that profoundly impacts health. While adaptive in acute contexts, prolonged or excessive stress imposes severe physical and psychological burdens [39]. The detrimental effects of stress on the central nervous system have been a major research focus. In this study, we elucidated the role of the gut microbiota in hippocampal neuronal ferroptosis under different durations of stress and identified key microbial taxa and metabolites, providing preliminary evidence for exploring the molecular mechanisms underlying stress-induced ferroptosis.

Ferroptosis, an iron-dependent form of cell death, is closely associated with neurological disorders [40]. Recent studies have indicated that stress disrupts hippocampal iron metabolism, which is characterized by elevated Fe^2^⁺ levels and dysregulated ferroptosis-related proteins [41, 42]. However, the direct causal relationship between stress-induced hippocampal damage and ferroptosis remains unclear. To address this, we employed ferroptosis inhibitors: Fer-1, which directly inhibits lipid peroxidation and modulates iron metabolism [43], and DFO, an iron chelator that prevents iron-catalyzed lipid peroxidation and subsequent membrane damage [44]. Our results revealed that Fer-1 and DFO significantly reduced hippocampal TF and TFR expression and Fe^2^⁺ accumulation, restored redox balance, and alleviated neuronal damage and depressive-like behaviors across stress durations. Mechanistically, TF and TFR are critical regulators of iron homeostasis, and their dysregulation leads to iron overload [45, 46]. Excessive Fe^2^⁺ reacts with intracellular hydrogen peroxide via Fenton reaction, generating highly reactive free radicals that disrupt redox homeostasis [47]. Concurrently, reduced GSH, a primary free radical scavenger, exacerbates lipid peroxidation, ultimately triggering ferroptosis [48]. These findings support the occurrence of hippocampal neuronal ferroptosis in stressed mice and its correlation with depressive-like behaviors.

Emerging evidence has demonstrated that stress exposure can reshape gut microbial communities. Acute stress triggers diminished microbial diversity and transient dysbiosis, whereas chronic stress induces sustained ecological disruption characterized by decreased beneficial bacteria and increased harmful bacteria [49]. Notably, the gut microbiota regulates iron metabolism and modulates cerebral lipid metabolism in conditions such as ischemic stroke [10, 50]. On the basis of these findings, we hypothesized that the gut microbiota is involved in stress-induced hippocampal ferroptosis. To test this hypothesis, we performed FMT, which involves transferring microbial communities between donors and recipients to establish causality [51]. For example, Ye et al. demonstrated that transplanting healthy microbiota ameliorated cognitive deficits in diabetic mice [52]. In our study, FMT from stress-exposed donors to antibiotic-treated recipients successfully induced ferroptotic phenotypes, suggesting the pivotal role of the gut microbiota in stress-induced hippocampal ferroptosis.

Further investigations revealed that the gut microbial composition was significantly altered in both time-course stress models and FMT recipient mice, with particularly pronounced dysregulation of SCFA-producing bacterial taxa. SCFAs, microbial metabolites from dietary fibre fermentation, exert anti-inflammatory and gut-protective effects [53]. These SCFAs (e.g., butyric acid and valeric acid) enter systemic circulation to modulate multiple organs, including the brain. Prior studies have shown that SCFAs promote hippocampal neurogenesis and protect the blood–brain barrier to alleviate depressive behaviors [54]. Using GC–MS, we observed decreased serum butyric acid in the 3-day stress model and reduced valeric acid in the 7-day model. Butyric acid supplementation inhibits ferroptosis in arthritis models [55], whereas butyric acid-enriched FMT alleviates hepatic ferroptosis [56]. Although the role of valeric acid in ferroptosis has been less well studied, it protects dopaminergic neurons by suppressing oxidative stress and neuroinflammation in Parkinson’s disease [57]. Consistent with these findings, butyric acid and valeric acid supplementation in our study significantly attenuated hippocampal ferroptosis and depressive-like behaviors in stress models, indicating their potential regulatory roles. It should be noted that as an exploratory study, these findings require further validation through larger-scale experiments and population-based studies.

**Fig. 9:**
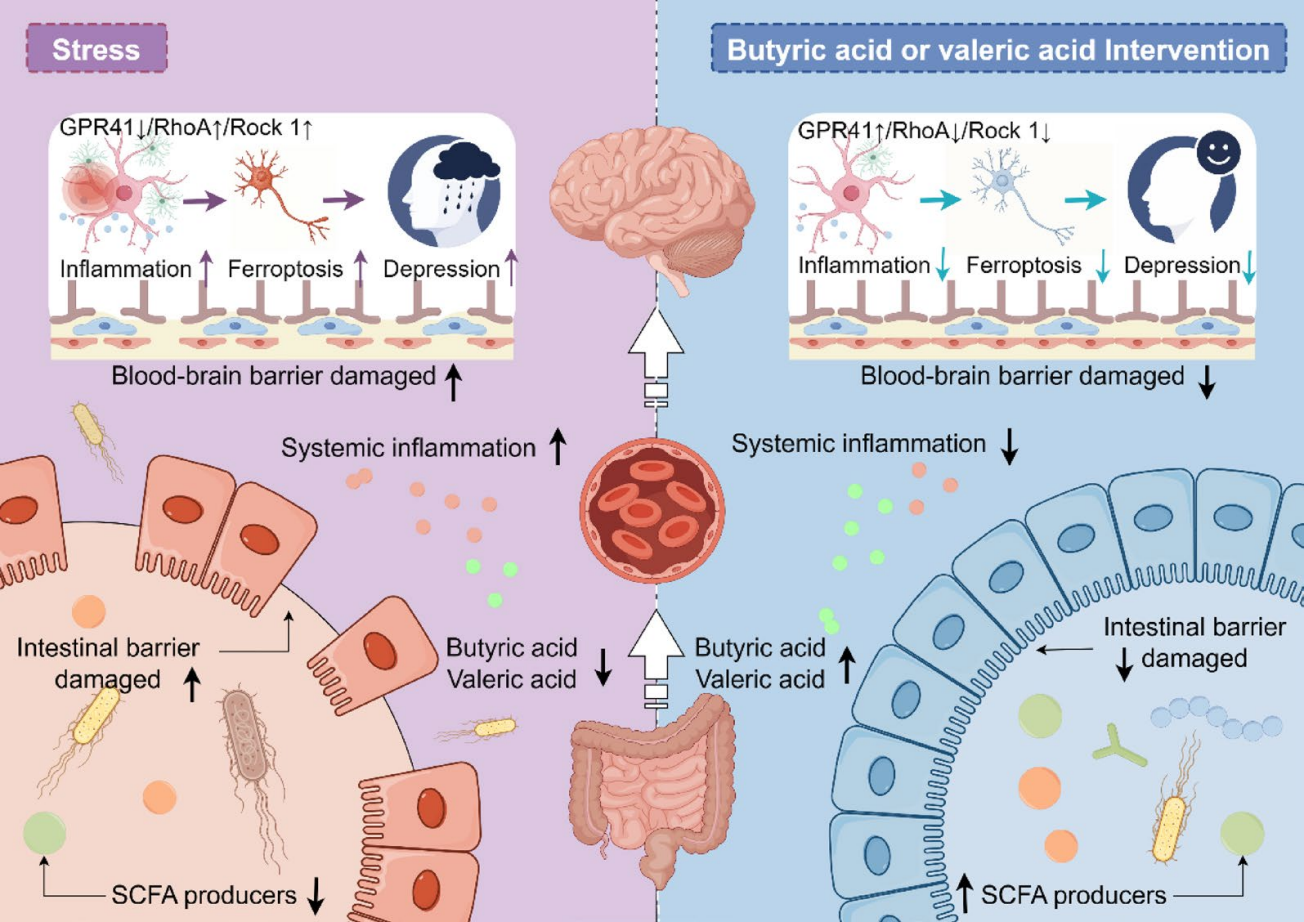
The schematic diagram depicting our findings. This diagram was created via Figdraw, ID: WIYOR4dadd

From a mechanistic perspective, bidirectional regulation between the gut and nervous system is mediated through the gut-brain axis [58]. Gut dysbiosis diminishes intestinal tight junction proteins, particularly Occludin and Claudin-5, thereby increasing gut permeability, a condition clinically termed leaky gut. This pathological state enables toxins and pathogens to enter systemic circulation, initiating widespread inflammation [59]. Proinflammatory cytokines, including IL-1β and IL-6, compromise blood–brain barrier integrity [60], permitting peripheral inflammatory mediators to infiltrate the central nervous system and induce neuroinflammatory responses [61]. Such neuroinflammation may drives lipid peroxidation while upregulating iron-regulatory proteins such as TFR, culminating in cytotoxic iron overload [31, 32]. Sheng et al. further demonstrated that IL-6 induces lipid peroxidation and iron dyshomeostasis in degenerative chondrocytes [62]. In this study, we identified the key gut microbiota metabolites butyric acid and valeric acid as anti-inflammatory agents. Our findings indicated that butyric acid and valeric acid supplementation significantly alleviated intestinal barrier and blood–brain barrier damage during stress, reduced serum and hippocampal inflammation, and attenuated hippocampal neuronal ferroptosis. Notably, our results suggest that valeric acid exerts anti-inflammatory effects in stressed hippocampi potentially through the GPR41/RhoA/Rock1 signalling pathway. GPR41, an SCFA receptor, is downregulated in chronic stress models, leading to the activation of the RhoA/Rock1 pathway, which is known to promote inflammation [38, 63, 64]. Valeric acid administration was associated with restored GPR41 expression and suppressed RhoA/Rock1 signalling, thereby reducing neuroinflammation and ferroptosis. However, this remains a correlation-based inference. Additional pathway inhibition/activation tests (e.g., pharmacological GPR41 blockade) will be performed to fully validate this proposed mechanism in future work. Interestingly, butyric acid-mediated protection likely operates through alternative mechanisms, as no significant alterations in the GPR41/RhoA/Rock1 pathway were observed in the butyric acid-treated groups. This divergence underscores the complexity of SCFA signalling and suggests distinct neuroprotective mechanisms among individual SCFAs. Collectively, our findings preliminarily reveal the regulatory mechanisms of the gut-brain axis in modulating stress-associated ferroptosis and corresponding neuroprotective pathways, as summarized in Fig. 9.

## Conclusion

This study demonstrated that ferroptosis inhibitors effectively confirmed hippocampal neuronal ferroptosis in stress models of different durations and identified temporally distinct microbial metabolites, butyric acid in acute (3-day) and valeric acid in chronic (7-day) stress models. Furthermore, we established that butyric acid and valeric acid alleviate ferroptosis and depressive-like behaviors through the gut-brain axis-mediated suppression of inflammatory responses. Crucially, the GPR41/RhoA/Rock1-mediated neuro-inflammatory modulation was involved in valeric acid’s suppression of chronic stress-induced hippocampal ferroptosis, whereas the acute stress protection provided by butyric acid operates through various pathways, a priority for mechanistic exploration. These findings advance therapeutic strategies targeting microbiota-derived metabolites or ferroptosis pathways for the treatment of neuropsychiatric disorders.

## Supplementary Information

Below is the link to the electronic supplementary material.

Supplementary Material 1

Supplementary Material 2

## Data Availability

The datasets used and analyzed in the current study are available from corresponding authors on reasonable request.

## Abbreviations

Fer-1: Ferrostatin-1
DFO: Deferoxamine
FMT: Fecal microbiota transplantation
SCFA: Short-chain fatty acid
DMSO: Dimethyl sulfoxide
BA: Butyric acid
VA: Valeric acid
ASV: Amplicon sequence variant
GC–MS: Gas chromatography–mass spectrometry
HE: Hematoxylin and eosin
TF: Transferrin
TFR: Transferrin receptor
RT-qPCR: Real-time quantitative polymerase chain reaction
cDNA: Complementary DNA
ELISA: Enzyme-linked immunosorbent assay
GSH: Glutathione
GSSG: Oxidized glutathione
MDA: Malondialdehyde
