# Supplementary material for: Butyric acid and valeric acid attenuate stress-induced ferroptosis and depressive-like behaviors by suppressing hippocampal neuroinflammation
Source: J Transl Med. 2025 Sep 2;23:974. doi: 10.1186/s12967-025-06950-0 (PMC12403447; doi:10.1186/s12967-025-06950-0)
Supplement: Supplementary file 1 — Supplementary Material 1 [file 12967_2025_6950_MOESM1_ESM.pdf]

In this study, the target protein and internal control were sourced from the same membrane. Due to different primary antibody origins, secondary antibodies were either anti-mouse or anti-rabbit, leading to separate channel displays. While shown in distinct images, we ensure that the target protein and the internal control both come from the same membrane.

Fig. 1D TF-3 d

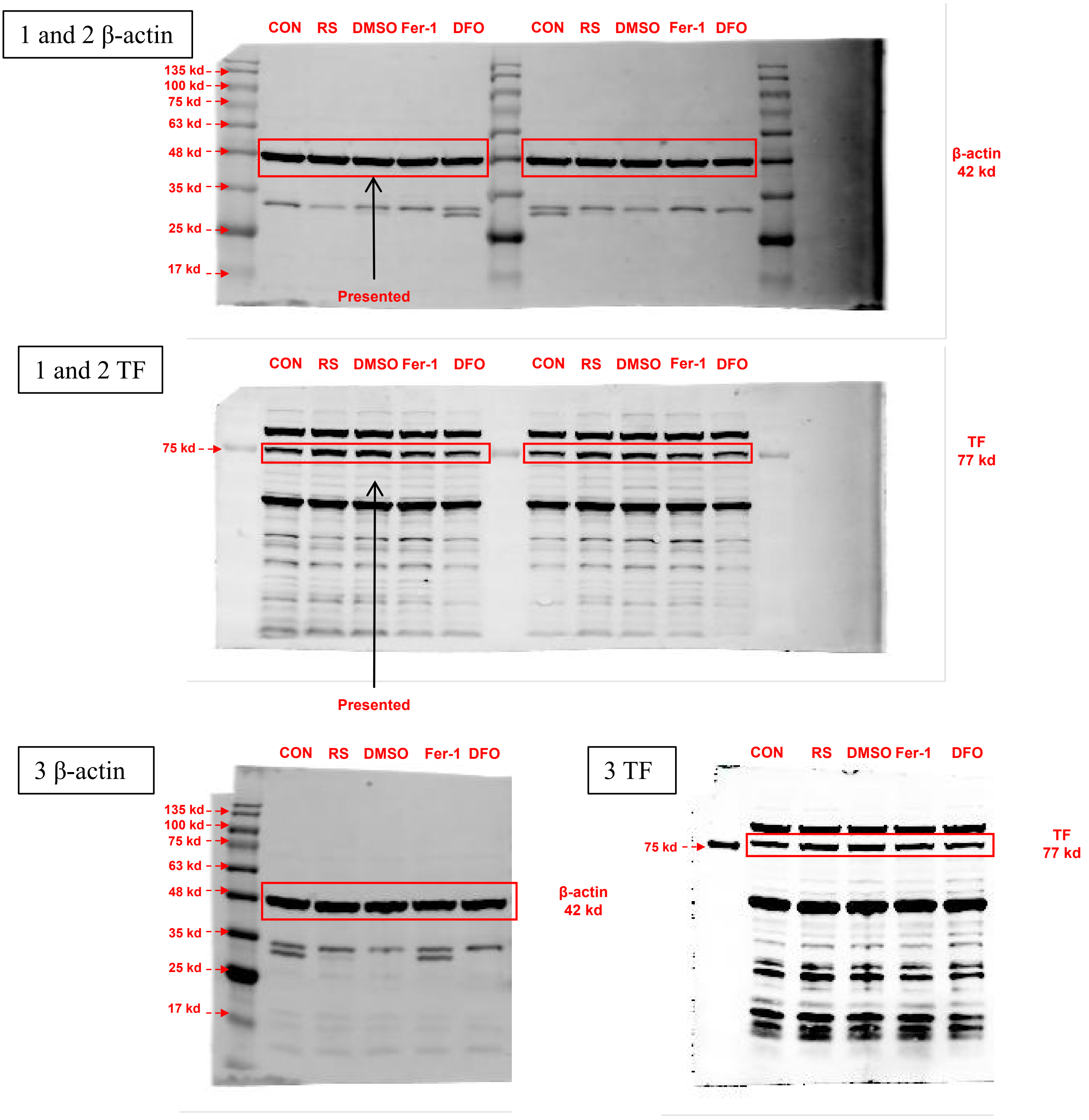

Fig. 1D TFR-3 d

1 β-actin and TFR

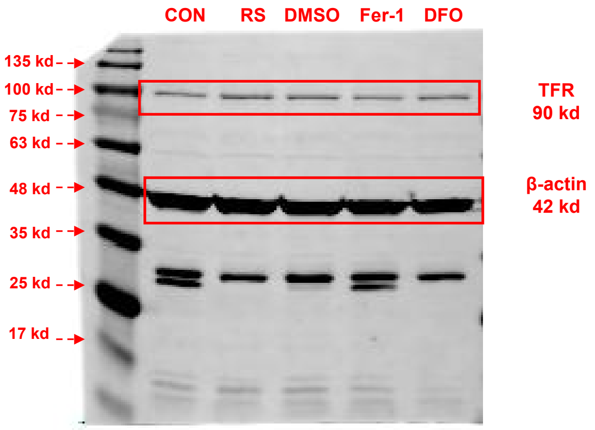

2 and 3 β-actin and TFR

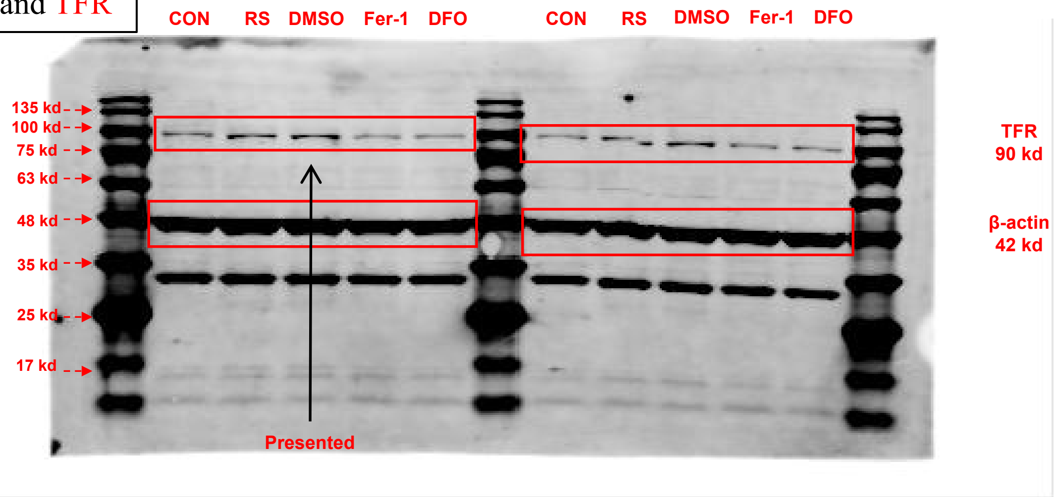

2 and 3 β-actin and TFR

This is the same membrane as above, but with a lighter version to make β-actin clearer.

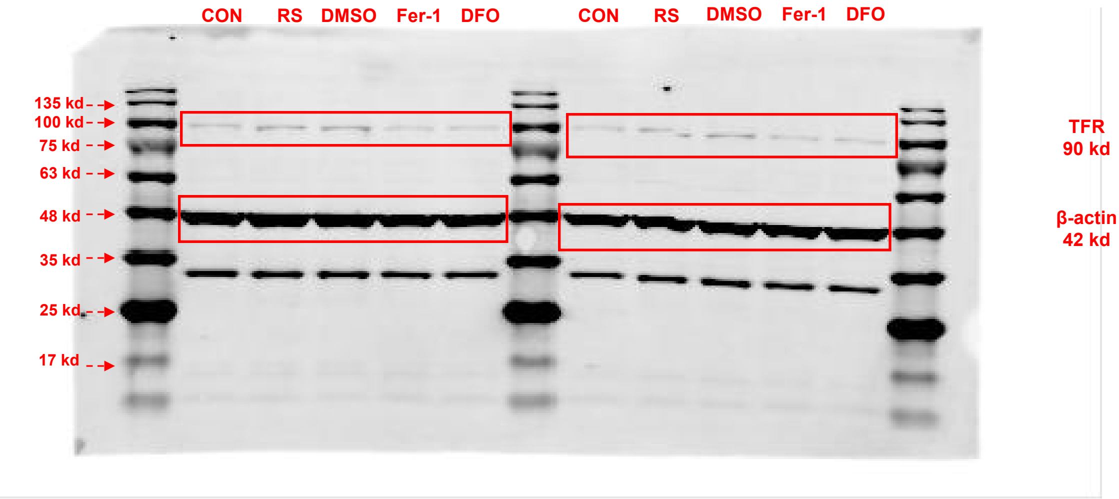

Fig. 1D TF-7 d

1 β-actin

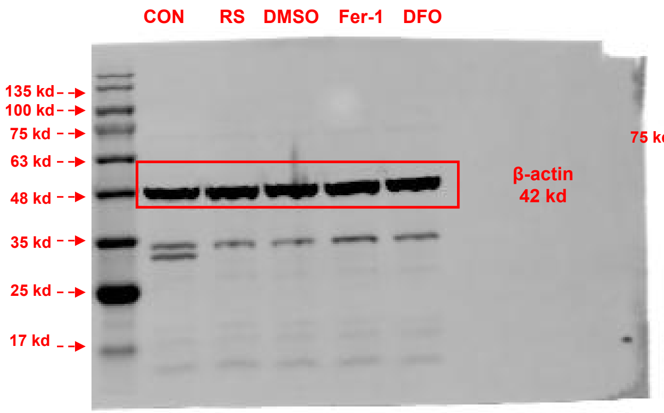

1 TF

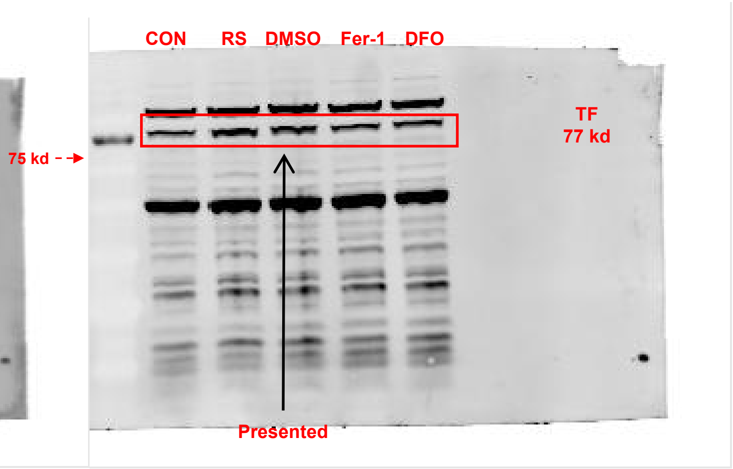

2 and 3 β-actin

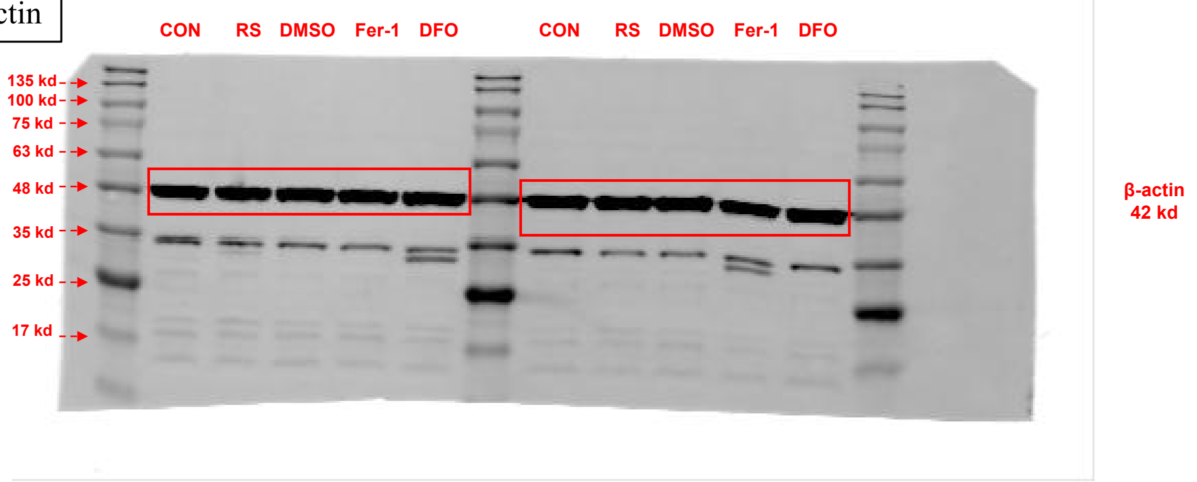

2 and 3 TF

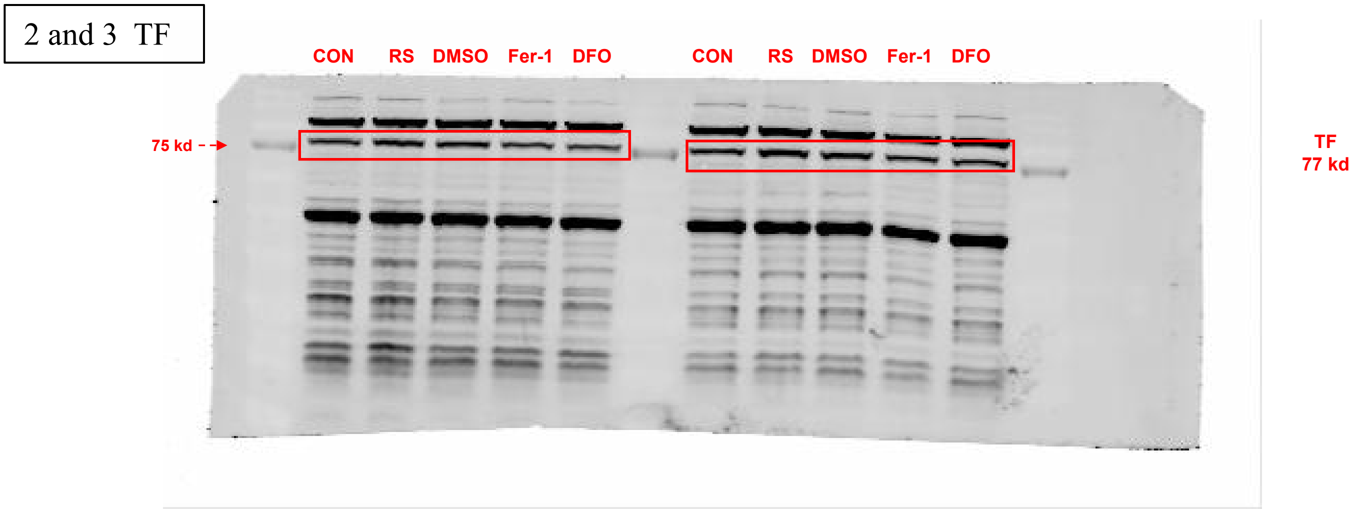

Fig. 1D TFR-7 d

1 and 2 β-actin and TFR

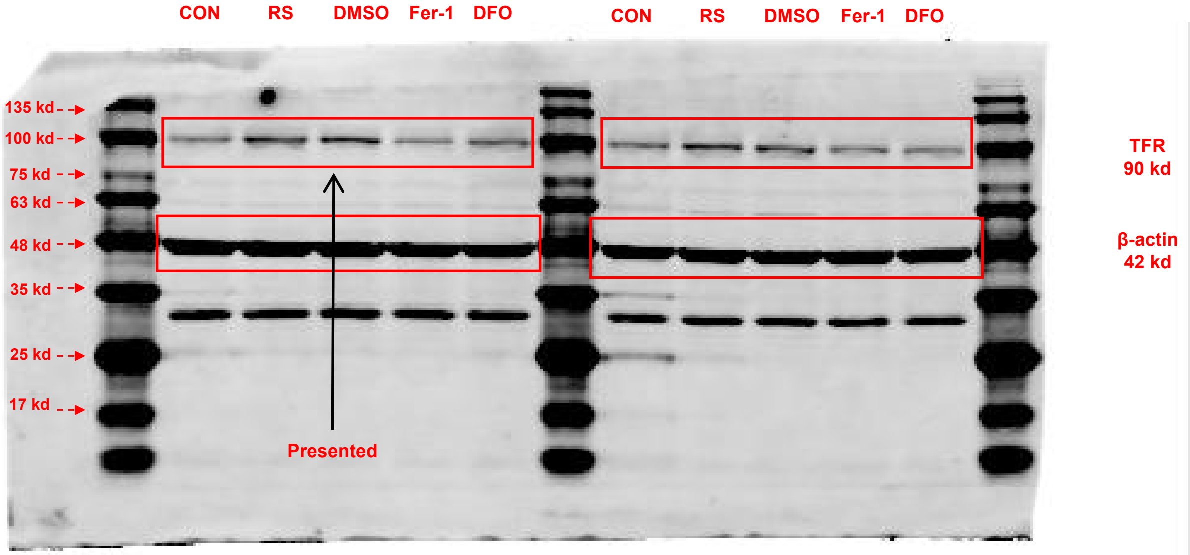

3 β-actin and TFR

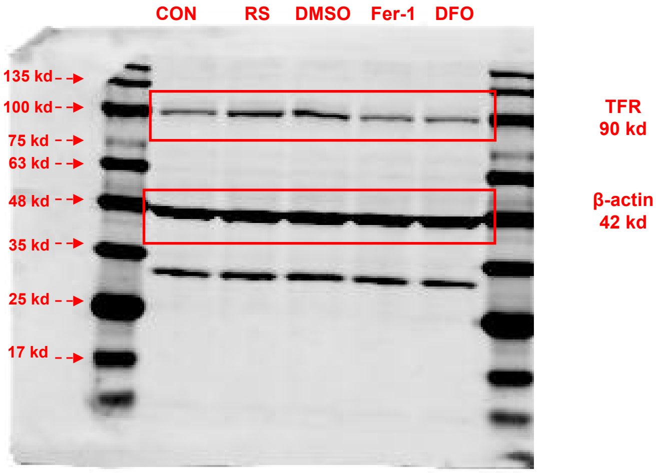

Fig. 2F TF FC-3 d and FRS-3 d

1, 2 and 3 β-actin

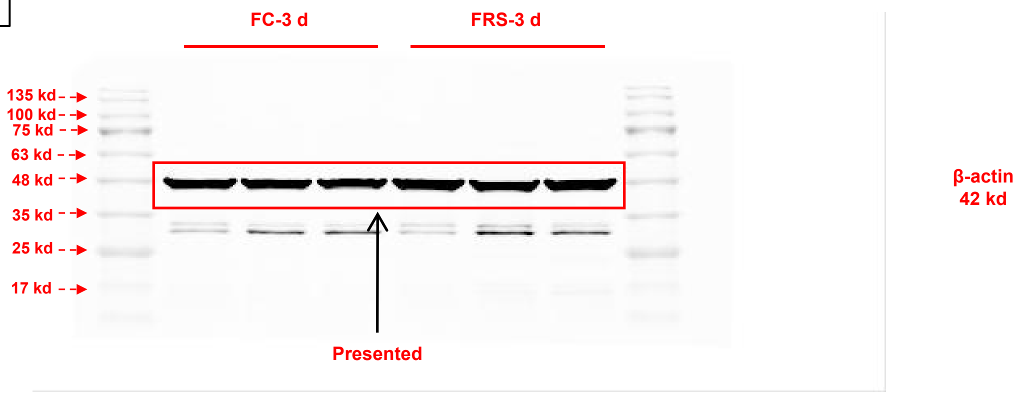

1, 2 and 3 TF

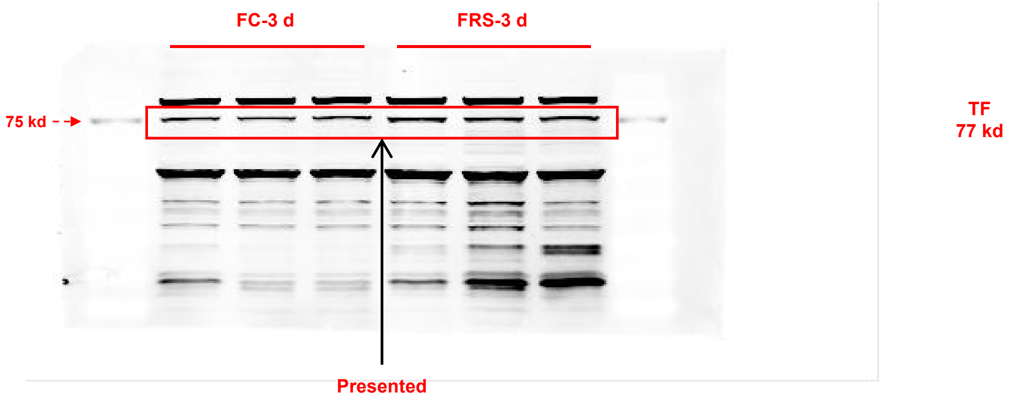

Fig. 2F TFR FC-3 d and FRS-3 d

1, 2, 3 β-actin and TFR

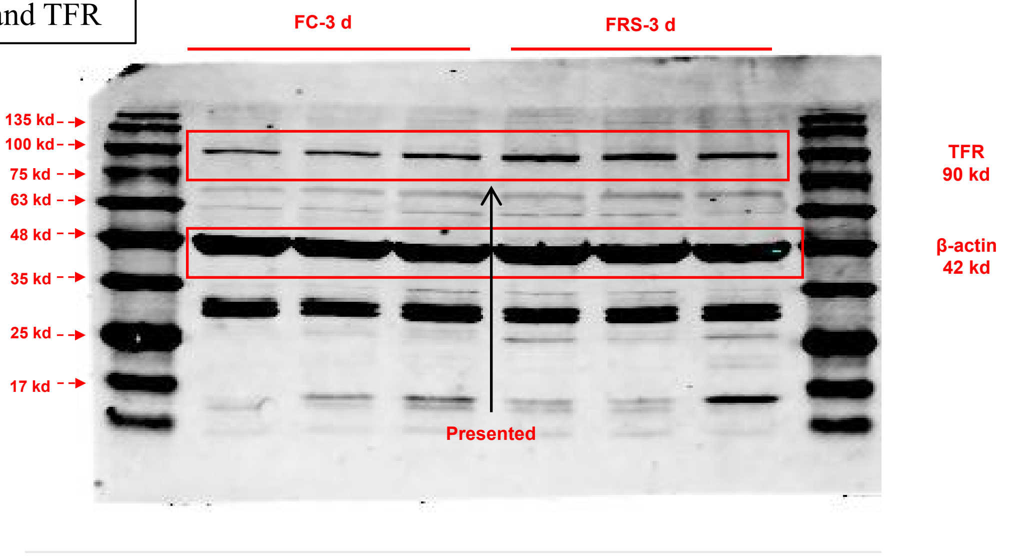

Fig. 2F TF FC-7 d and FRS-7 d

1, 2 and 3 β-actin

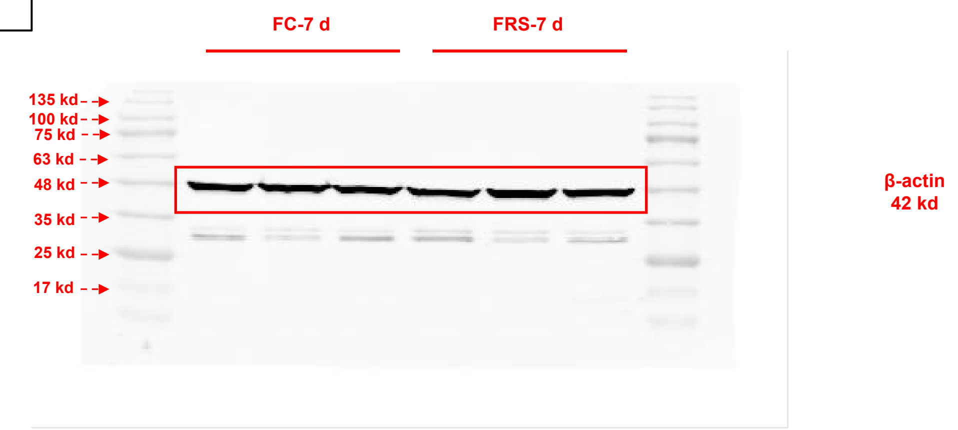

1, 2 and 3 TF

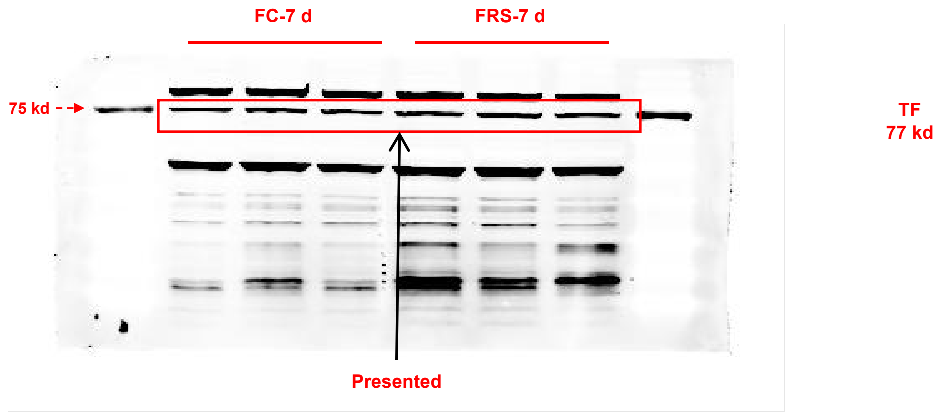

Fig. 2F TFR FC-7 d and FRS-7 d

1, 2, 3 β-actin and TFR

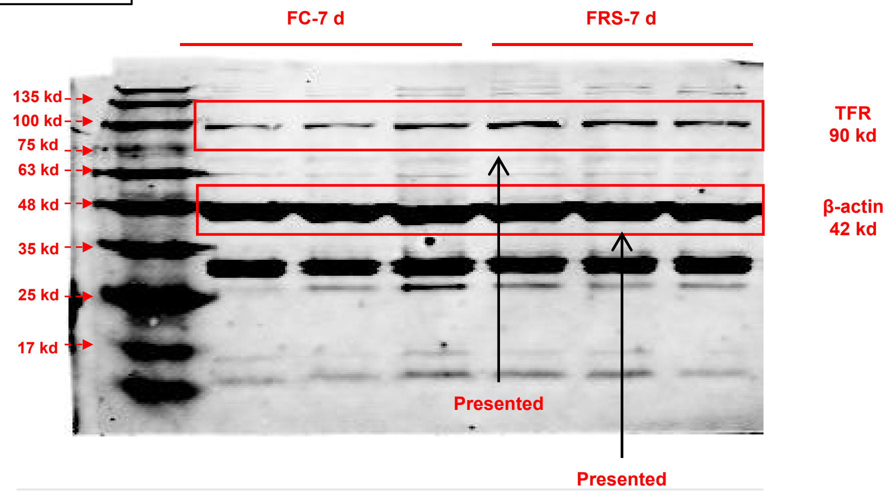

Fig. 6C TF

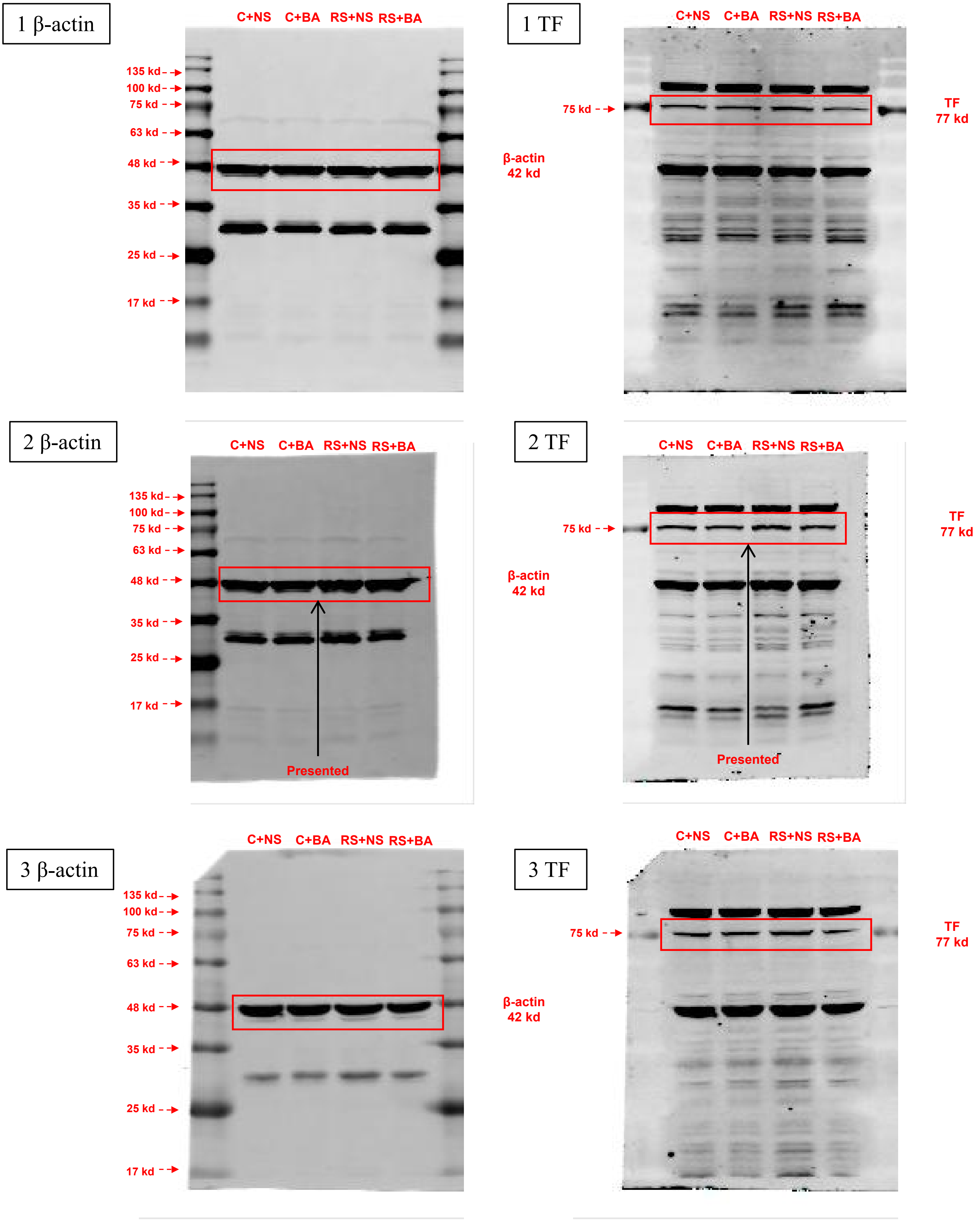
1 β-actin
C+NS C+BA RS+NS RS+BA
135 kd
100 kd
75 kd
63 kd
48 kd
35 kd
25 kd
17 kd
β-actin 42 kd
1 TF
C+NS C+BA RS+NS RS+BA
75 kd
TF 77 kd
2 β-actin
C+NS C+BA RS+NS RS+BA
135 kd
100 kd
75 kd
63 kd
48 kd
35 kd
25 kd
17 kd
Presented
β-actin 42 kd
2 TF
C+NS C+BA RS+NS RS+BA
75 kd
TF 77 kd
Presented
3 β-actin
C+NS C+BA RS+NS RS+BA
135 kd
100 kd
75 kd
63 kd
48 kd
35 kd
25 kd
17 kd
β-actin 42 kd
3 TF
C+NS C+BA RS+NS RS+BA
75 kd
TF 77 kd

Fig. 6C   TFR

1 β-actin and TFR

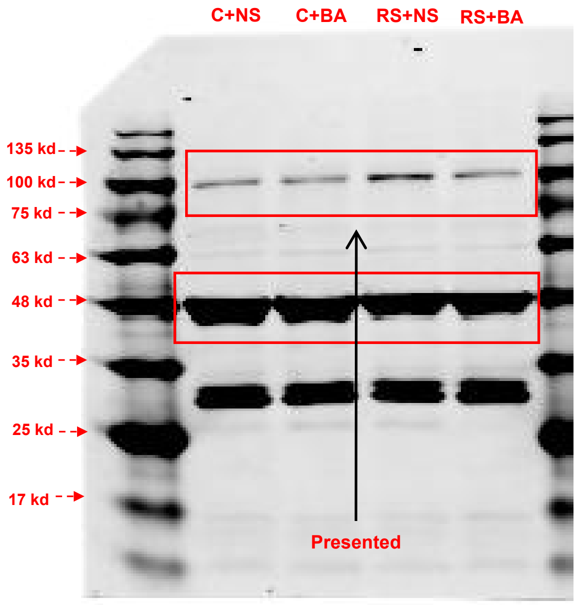

2 β-actin and TFR

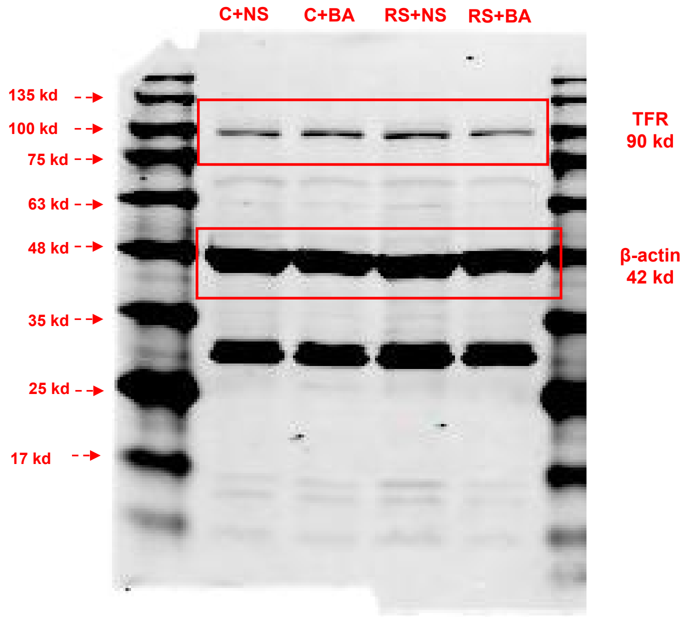

3 β-actin and TFR

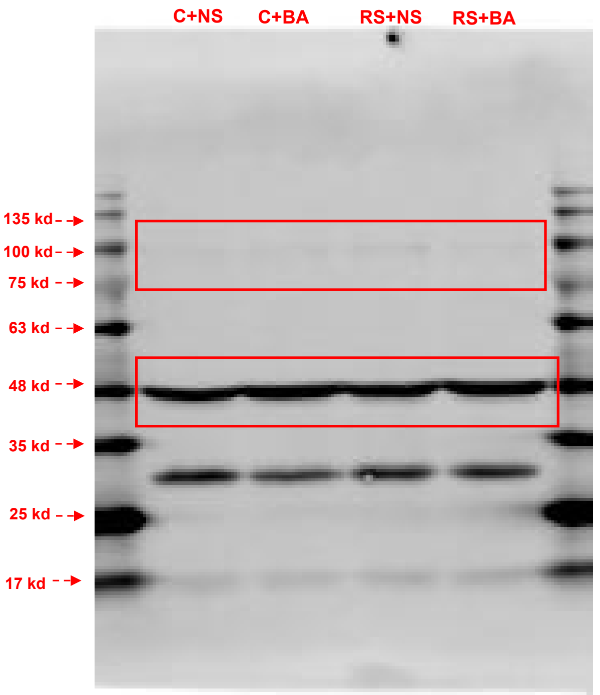

3 β-actin and TFR

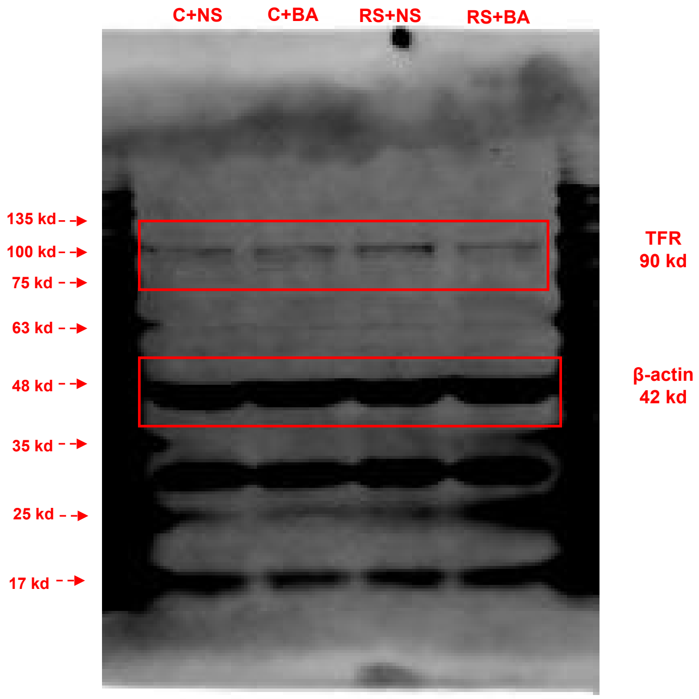

This is the same membrane as on the left, but darkened to highlight the target protein TFR.

Fig. 6E TF

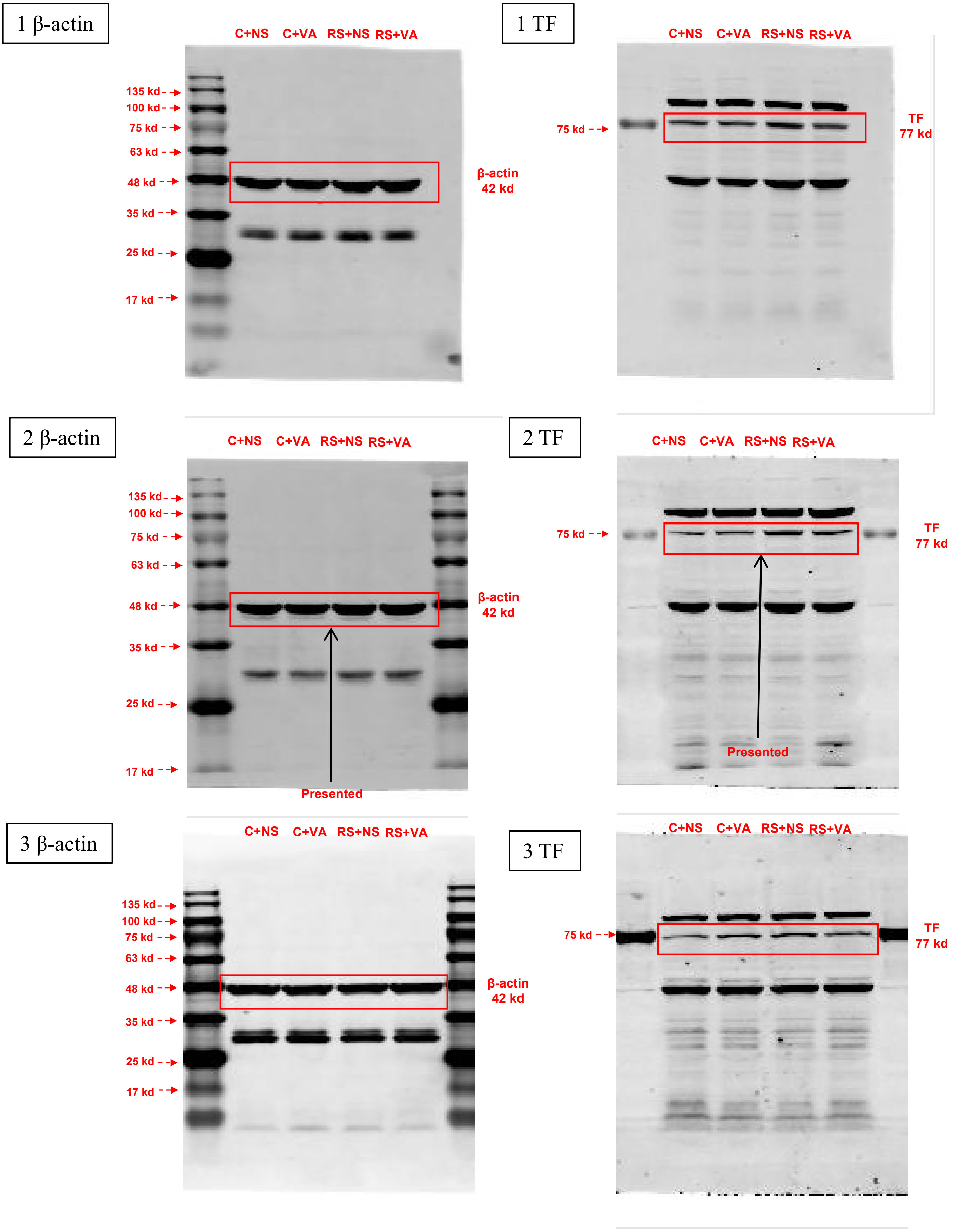

1 β-actin
C+NS C+VA RS+NS RS+VA
135 kd
100 kd
75 kd
63 kd
48 kd
35 kd
25 kd
17 kd
β-actin
42 kd
1 TF
C+NS C+VA RS+NS RS+VA
75 kd
TF
77 kd
2 β-actin
C+NS C+VA RS+NS RS+VA
135 kd
100 kd
75 kd
63 kd
48 kd
35 kd
25 kd
17 kd
β-actin
42 kd
Presented
2 TF
C+NS C+VA RS+NS RS+VA
75 kd
TF
77 kd
Presented
3 β-actin
C+NS C+VA RS+NS RS+VA
135 kd
100 kd
75 kd
63 kd
48 kd
35 kd
25 kd
17 kd
β-actin
42 kd
3 TF
C+NS C+VA RS+NS RS+VA
75 kd
TF
77 kd

Fig. 6E TFR

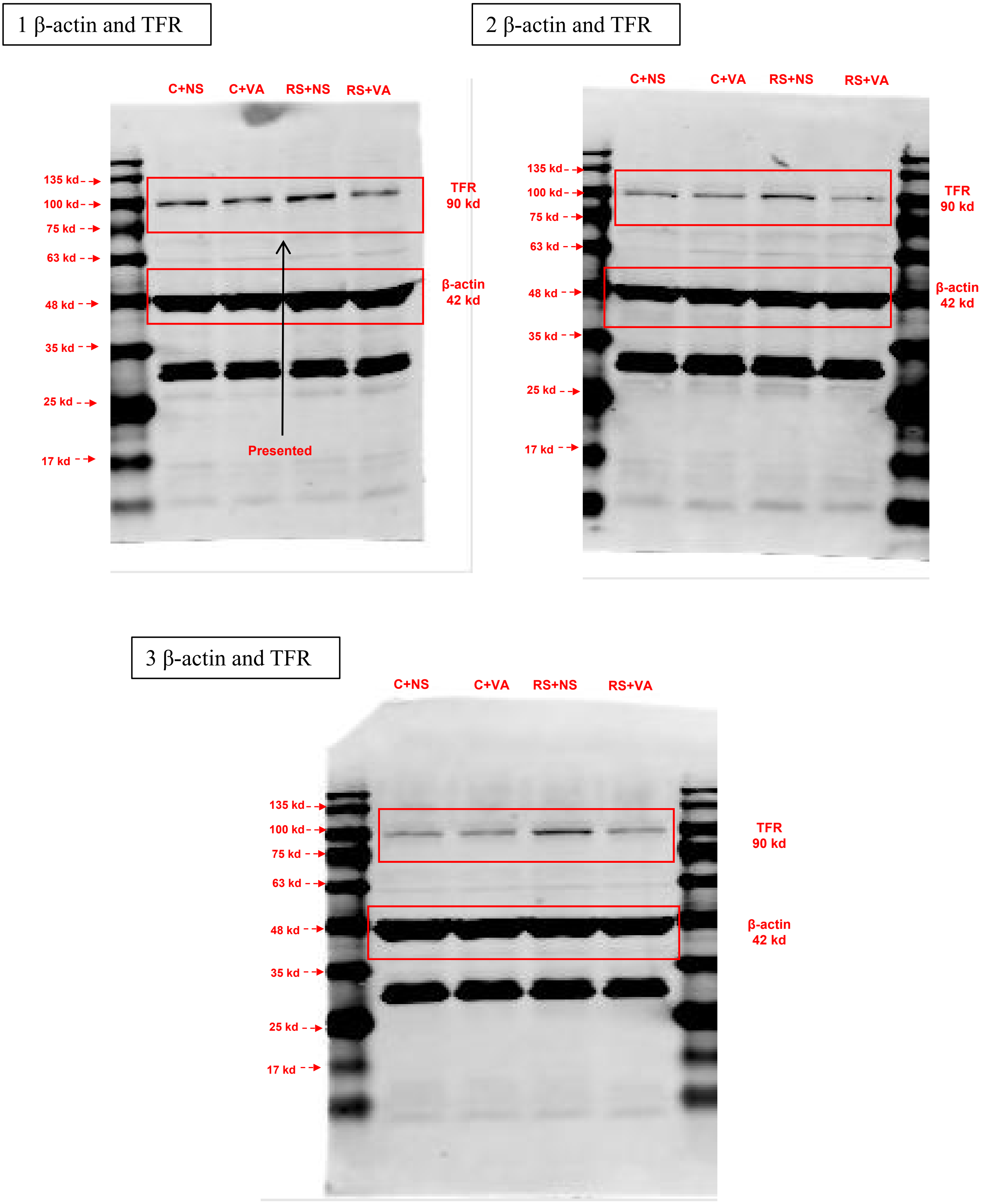
1 β-actin and TFR
C+NS
C+VA
RS+NS
RS+VA
135 kd
100 kd
75 kd
63 kd
48 kd
35 kd
25 kd
17 kd
TFR
90 kd
β-actin
42 kd
Presented
2 β-actin and TFR
C+NS
C+VA
RS+NS
RS+VA
135 kd
100 kd
75 kd
63 kd
48 kd
35 kd
25 kd
17 kd
TFR
90 kd
β-actin
42 kd
3 β-actin and TFR
C+NS
C+VA
RS+NS
RS+VA
135 kd
100 kd
75 kd
63 kd
48 kd
35 kd
25 kd
17 kd
TFR
90 kd
β-actin
42 kd

Fig. 7D Occludin

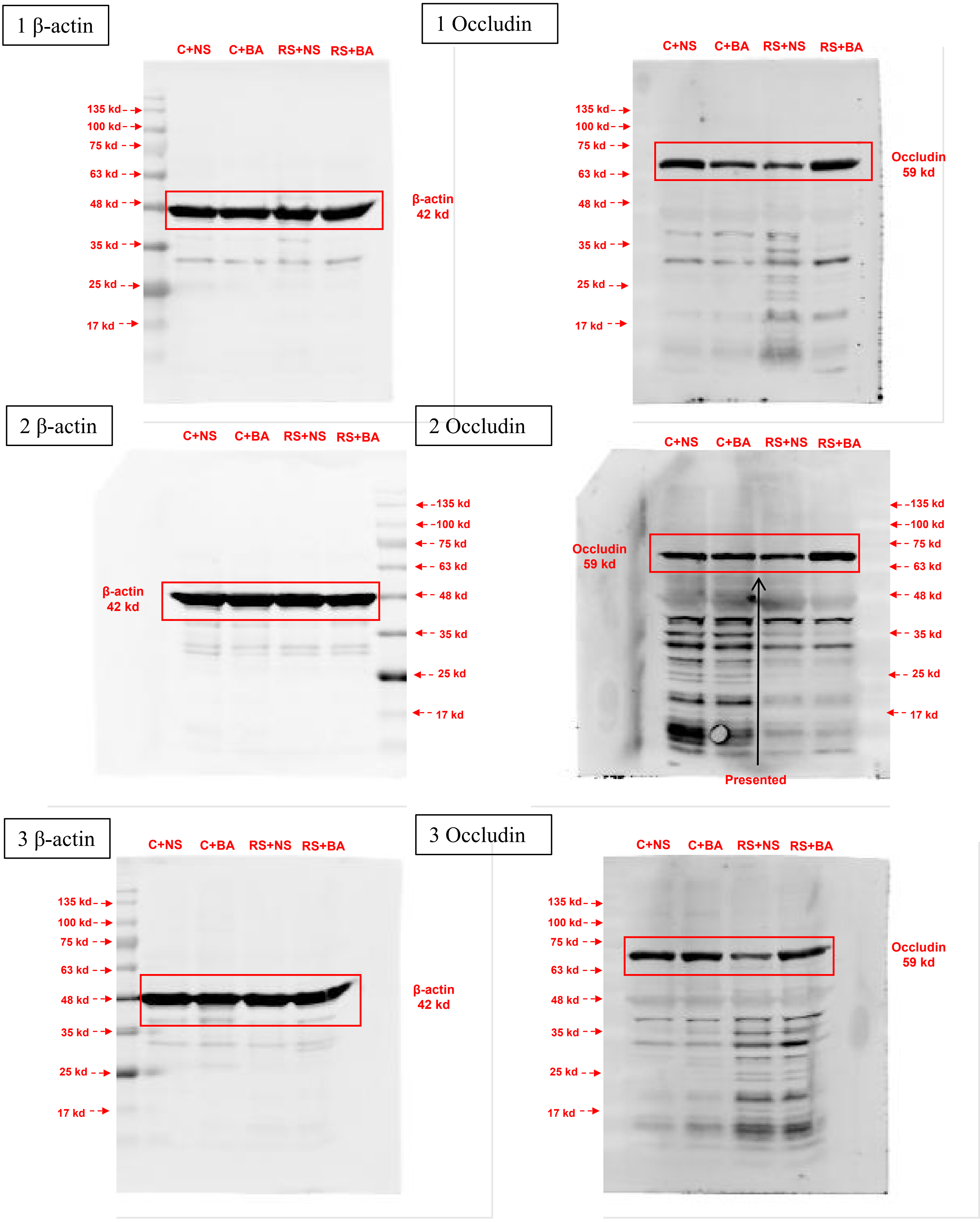

Fig. 7D Claudin 5

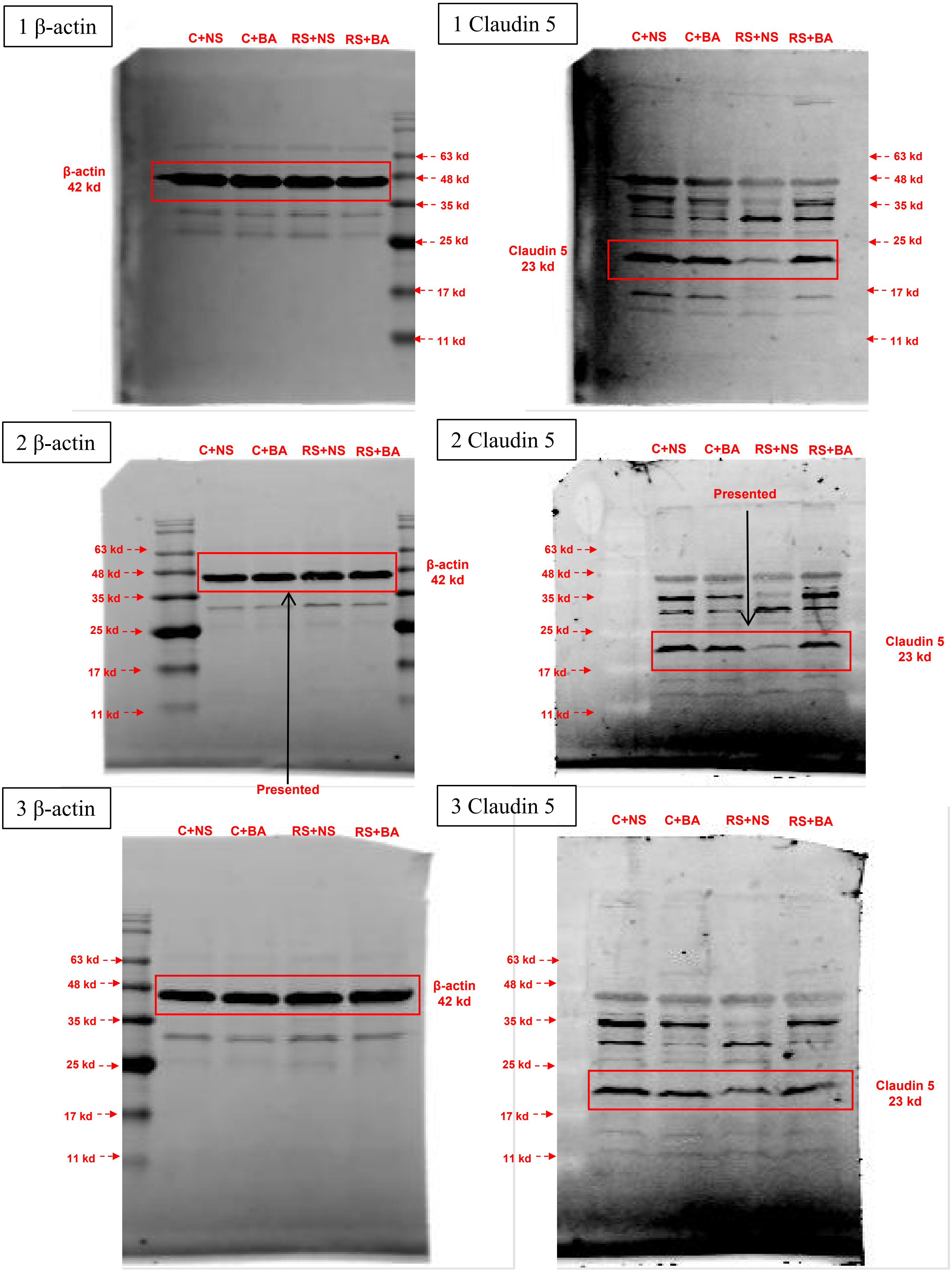

Fig. 7F Occludin

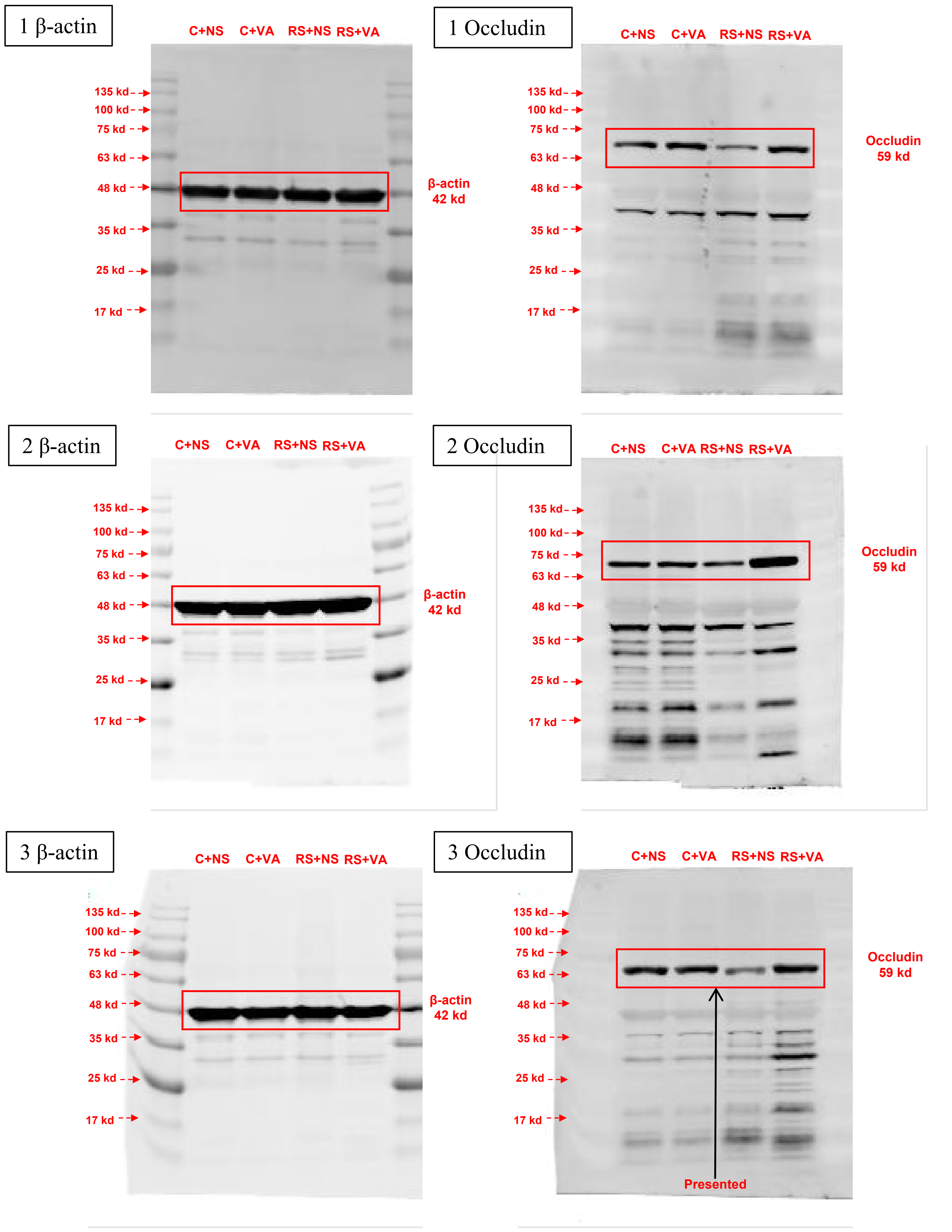

Fig. 7F Claudin 5

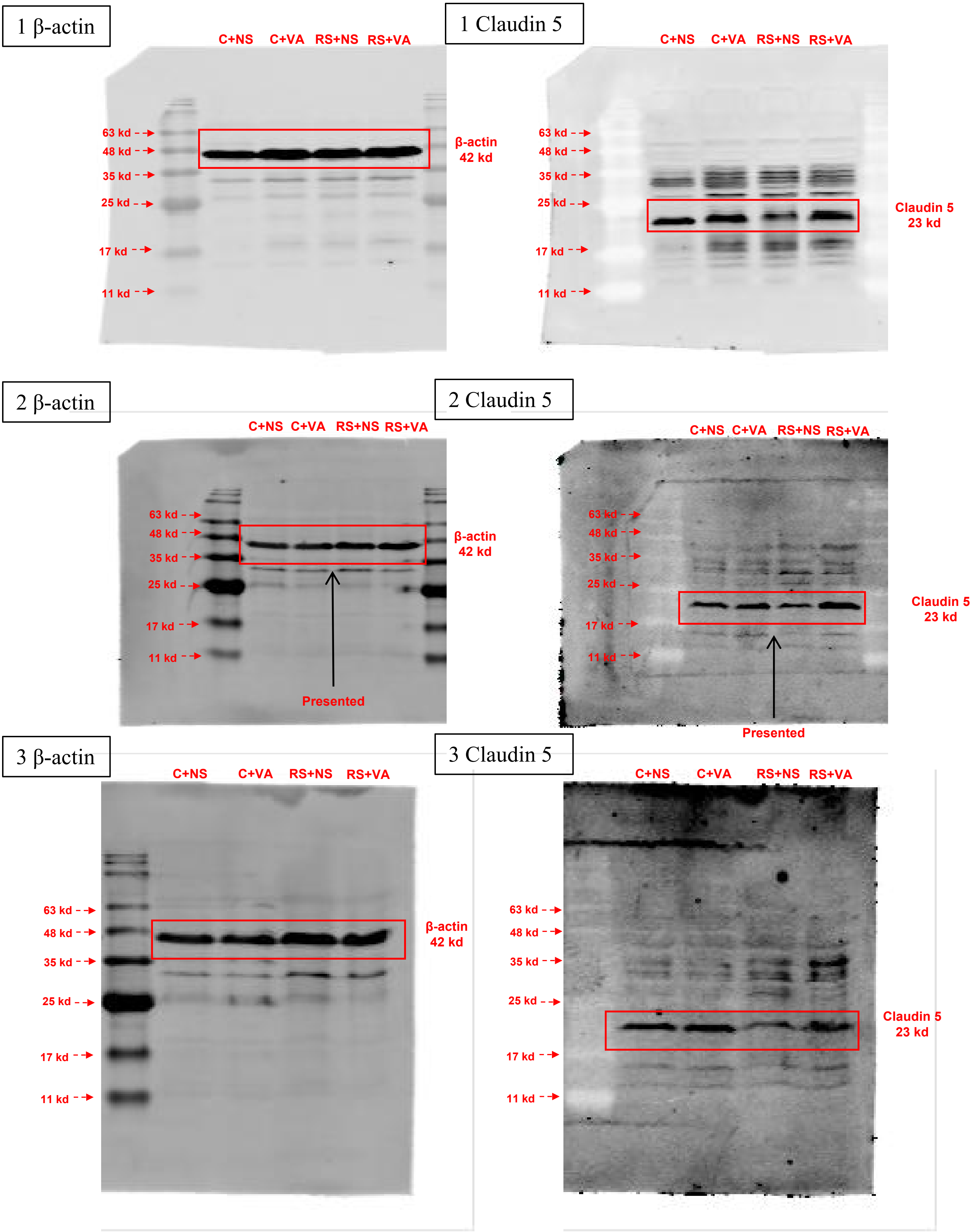

Fig. 8A Occludin

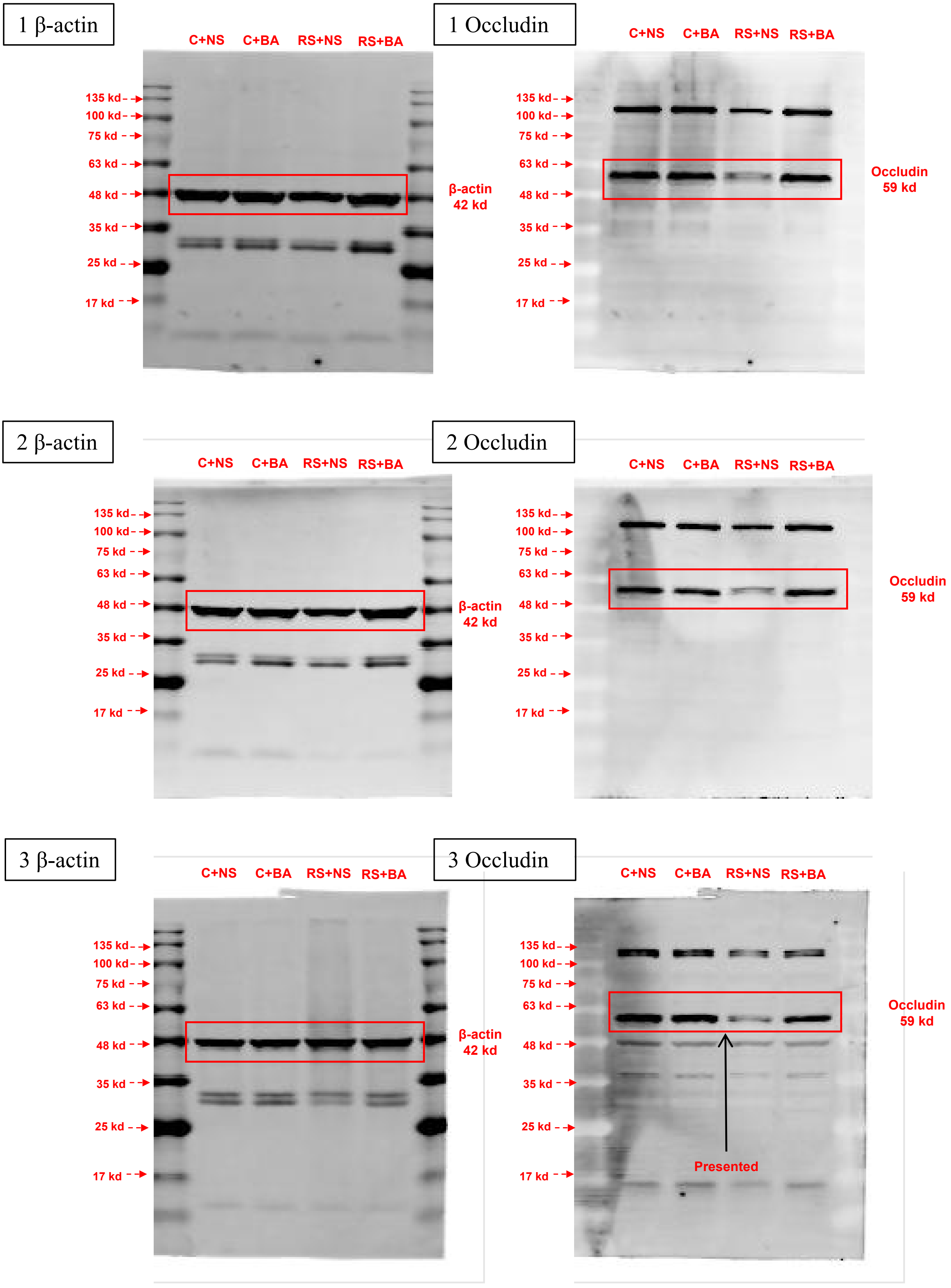

Fig. 8A Claudin 5

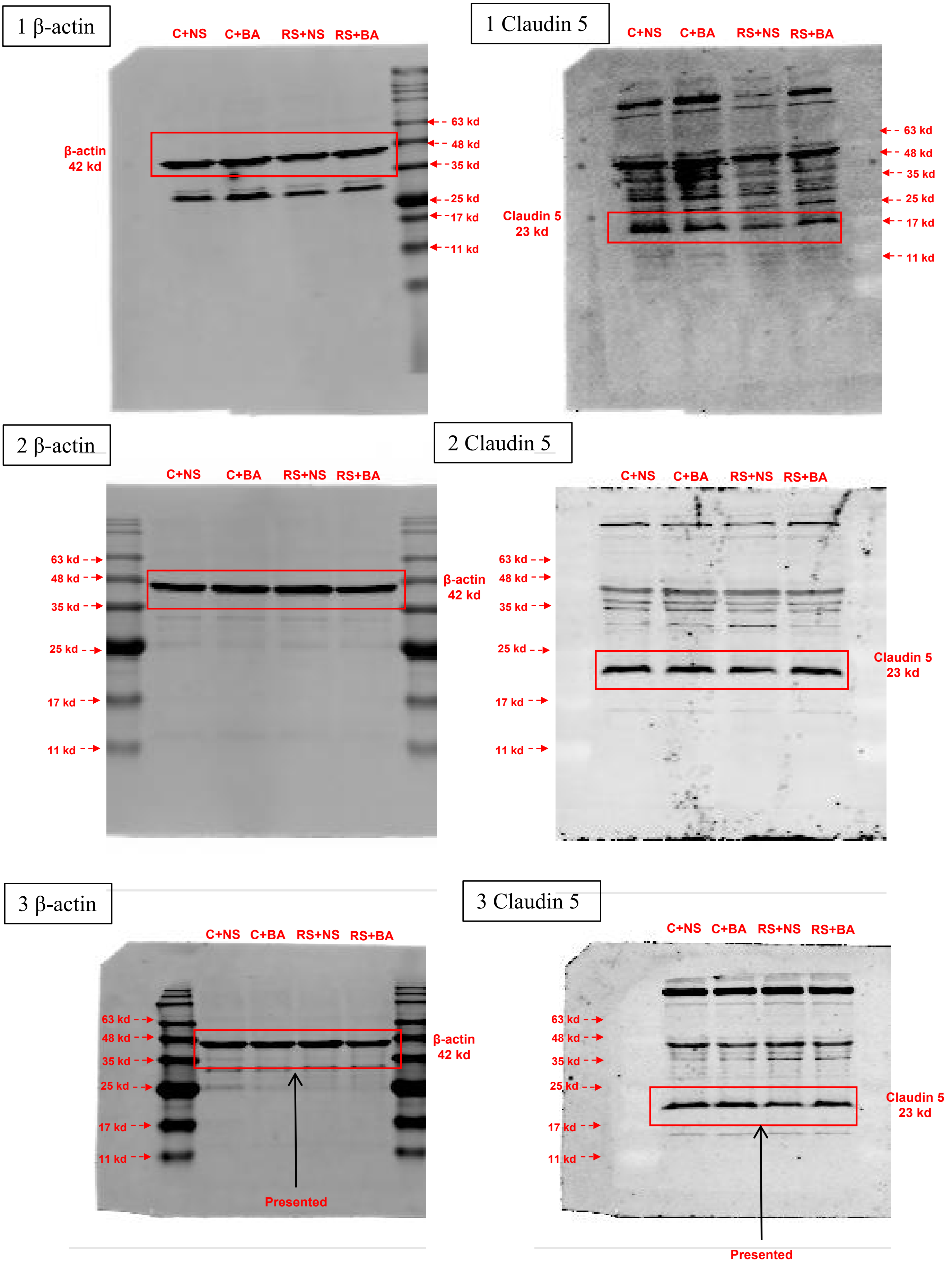

Fig. 8C Occludin

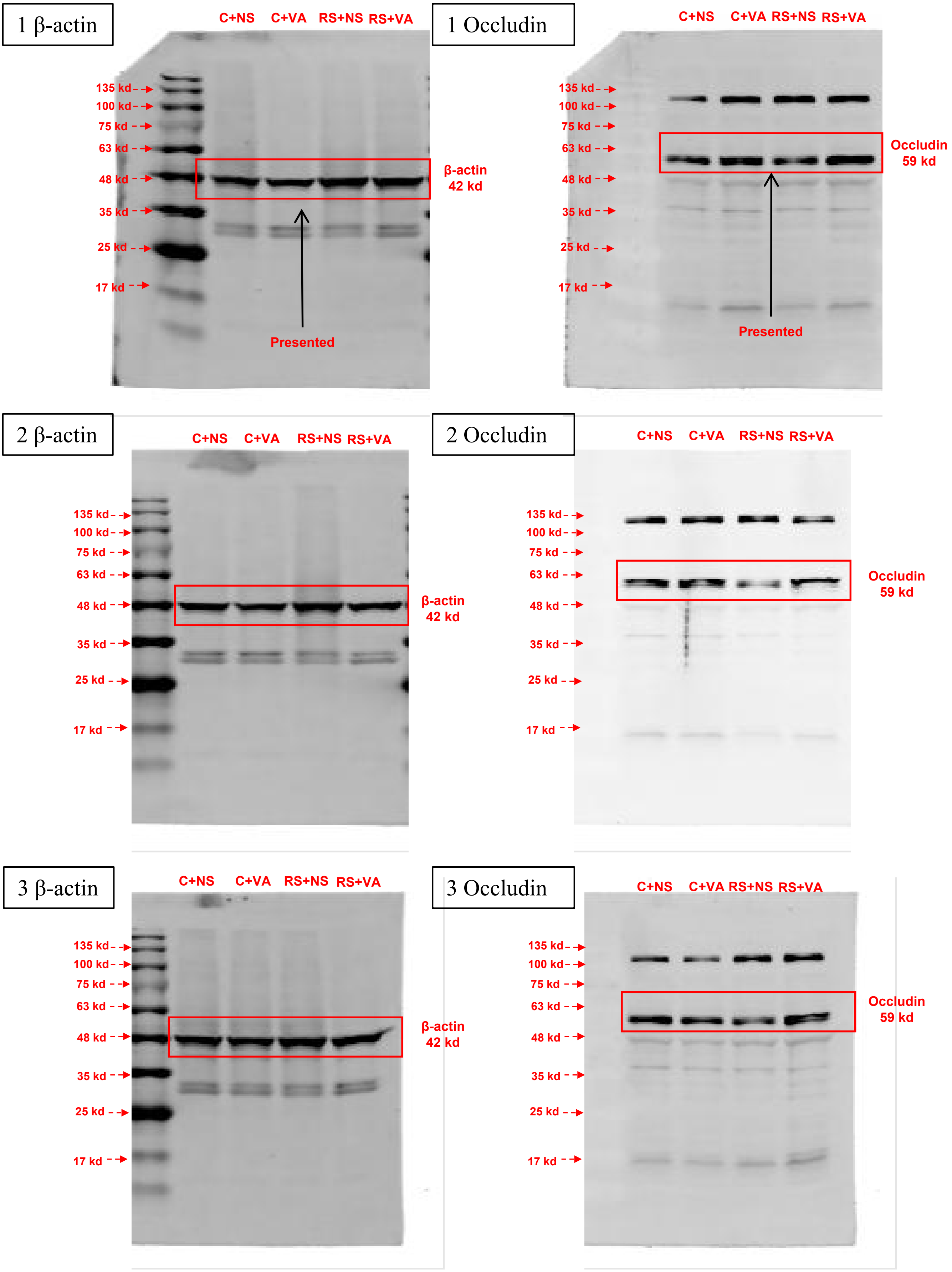

Fig. 8C Claudin 5

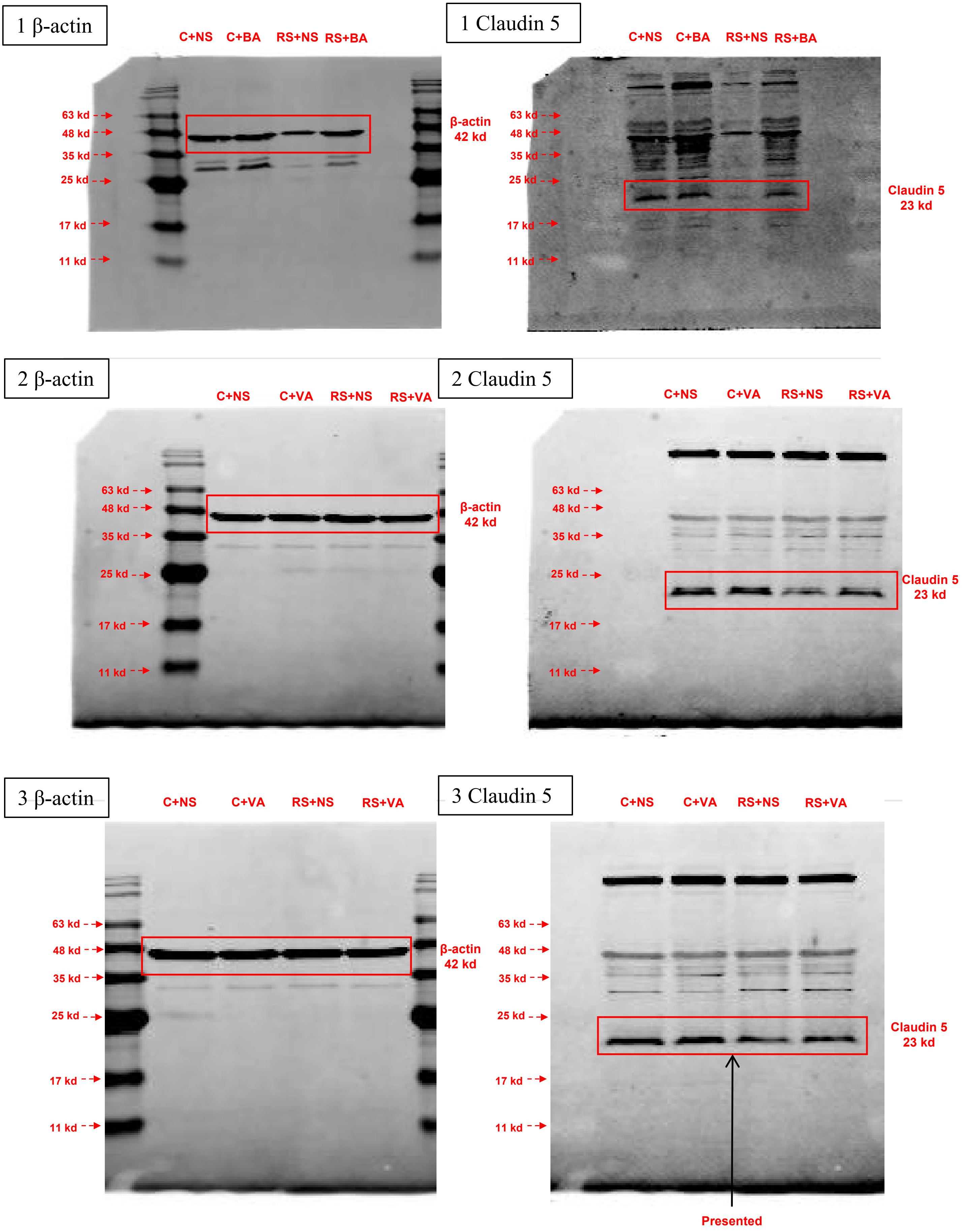

Fig. 8I GPR41

1 and 2 β-actin

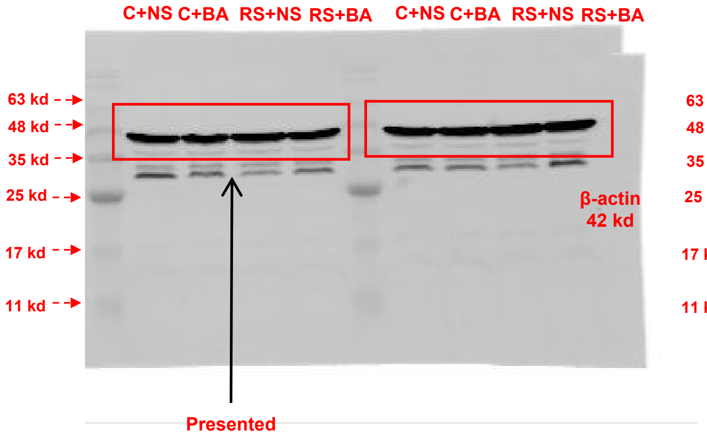

1 and 2 GPR41

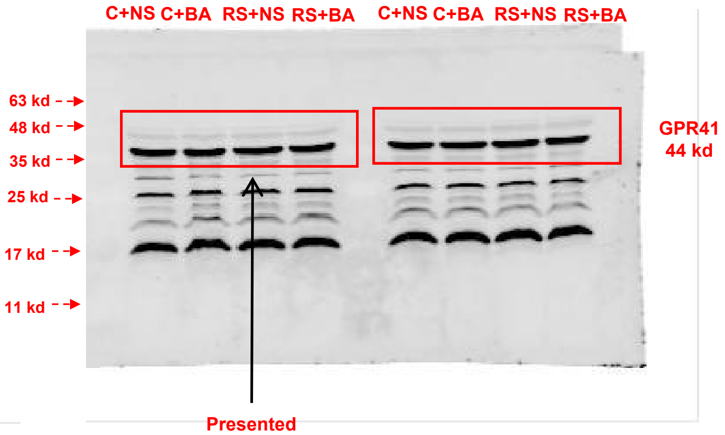

3 and 4 β-actin

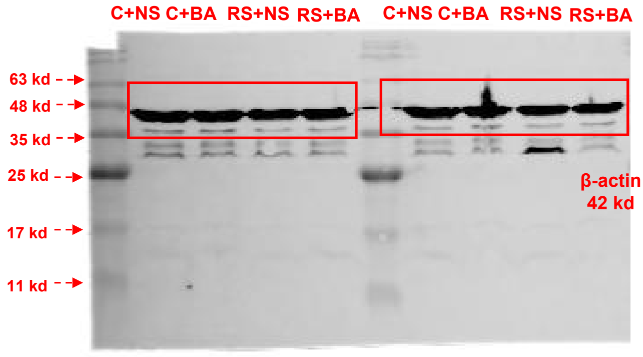

3 and 4 GPR41

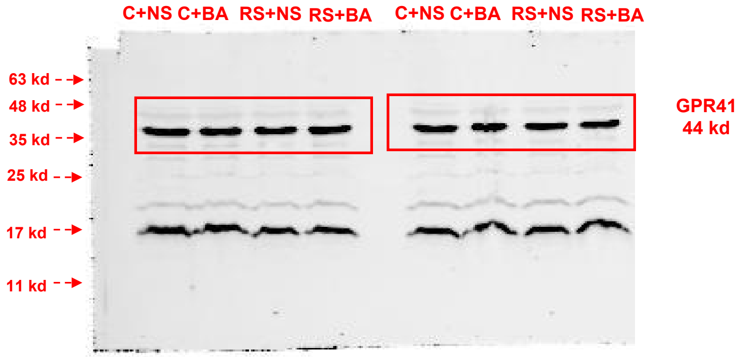

Fig. 8I RhoA

1 β-actin

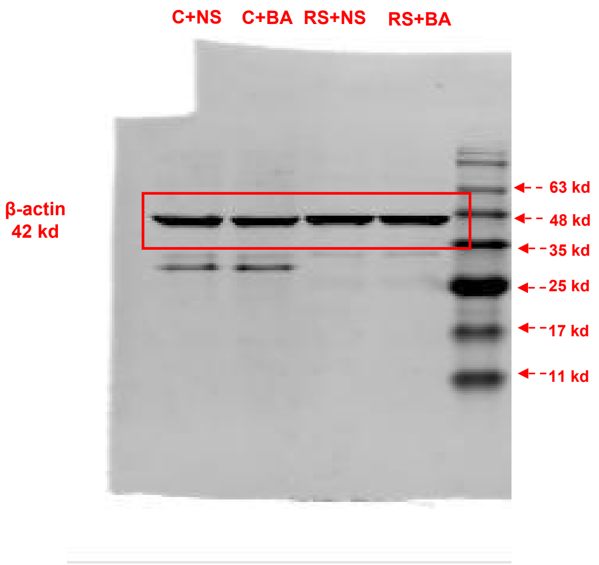

1 RhoA

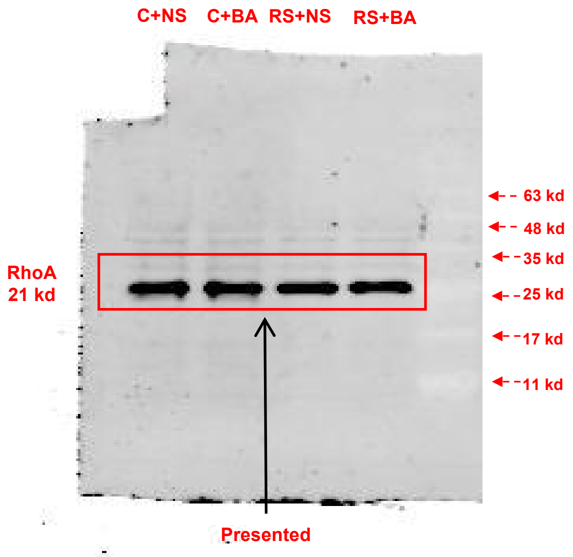

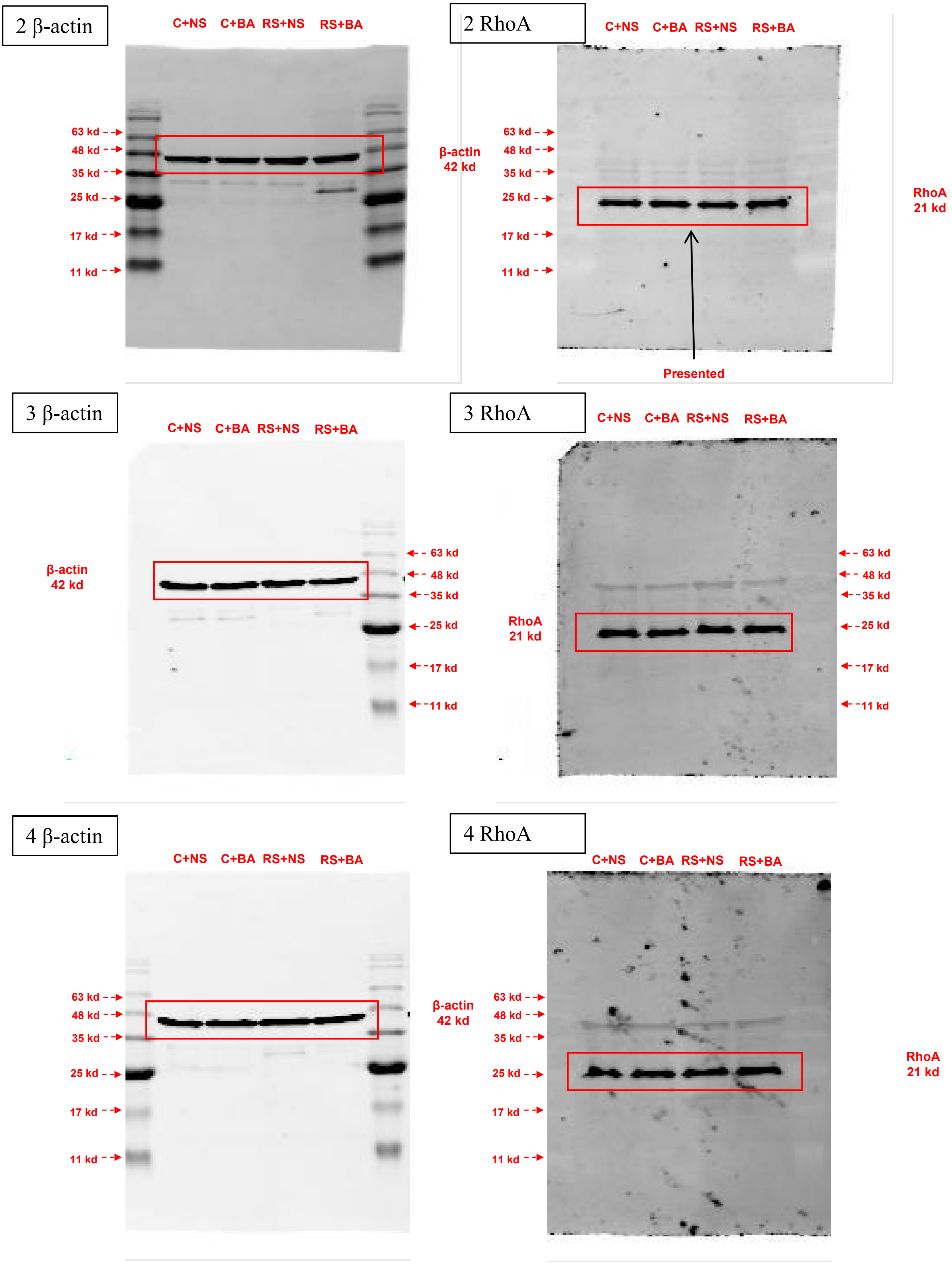
2 β-actin
C+NS C+BA RS+NS RS+BA
63 kd
48 kd
35 kd
25 kd
17 kd
11 kd
β-actin
42 kd
2 RhoA
C+NS C+BA RS+NS RS+BA
63 kd
48 kd
35 kd
25 kd
17 kd
11 kd
RhoA
21 kd
Presented
3 β-actin
C+NS C+BA RS+NS RS+BA
β-actin
42 kd
63 kd
48 kd
35 kd
25 kd
17 kd
11 kd
3 RhoA
C+NS C+BA RS+NS RS+BA
RhoA
21 kd
63 kd
48 kd
35 kd
25 kd
17 kd
11 kd
4 β-actin
C+NS C+BA RS+NS RS+BA
63 kd
48 kd
35 kd
25 kd
17 kd
11 kd
β-actin
42 kd
4 RhoA
C+NS C+BA RS+NS RS+BA
63 kd
48 kd
35 kd
25 kd
17 kd
11 kd
RhoA
21 kd

Fig. 8I Rock1

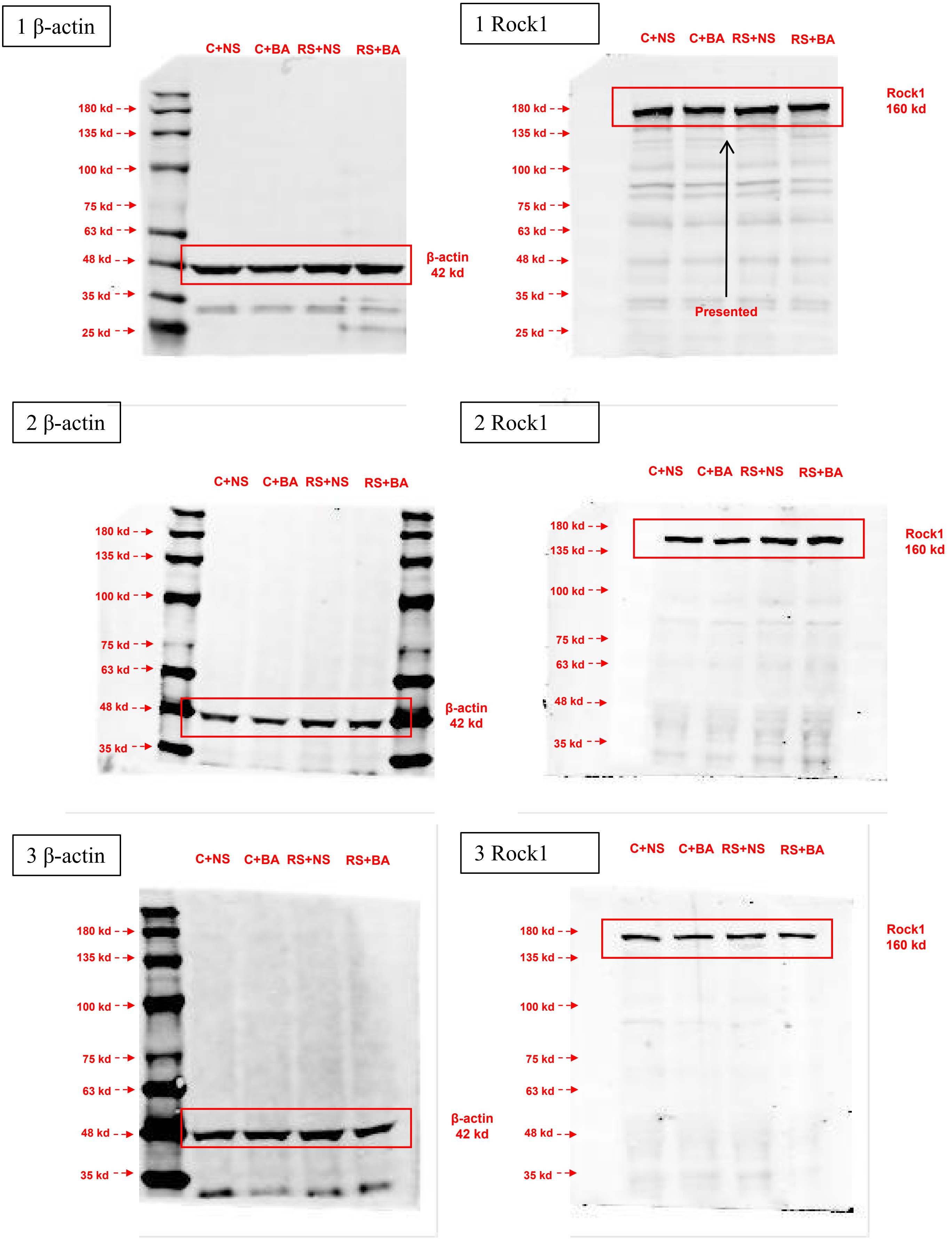

1 β-actin
C+NS C+BA RS+NS RS+BA
180 kd
135 kd
100 kd
75 kd
63 kd
48 kd
35 kd
25 kd
β-actin 42 kd
1 Rock1
C+NS C+BA RS+NS RS+BA
180 kd
135 kd
100 kd
75 kd
63 kd
48 kd
35 kd
25 kd
Rock1 160 kd
Presented
2 β-actin
C+NS C+BA RS+NS RS+BA
180 kd
135 kd
100 kd
75 kd
63 kd
48 kd
35 kd
β-actin 42 kd
2 Rock1
C+NS C+BA RS+NS RS+BA
180 kd
135 kd
100 kd
75 kd
63 kd
48 kd
35 kd
Rock1 160 kd
3 β-actin
C+NS C+BA RS+NS RS+BA
180 kd
135 kd
100 kd
75 kd
63 kd
48 kd
35 kd
β-actin 42 kd
3 Rock1
C+NS C+BA RS+NS RS+BA
180 kd
135 kd
100 kd
75 kd
63 kd
48 kd
35 kd
Rock1 160 kd

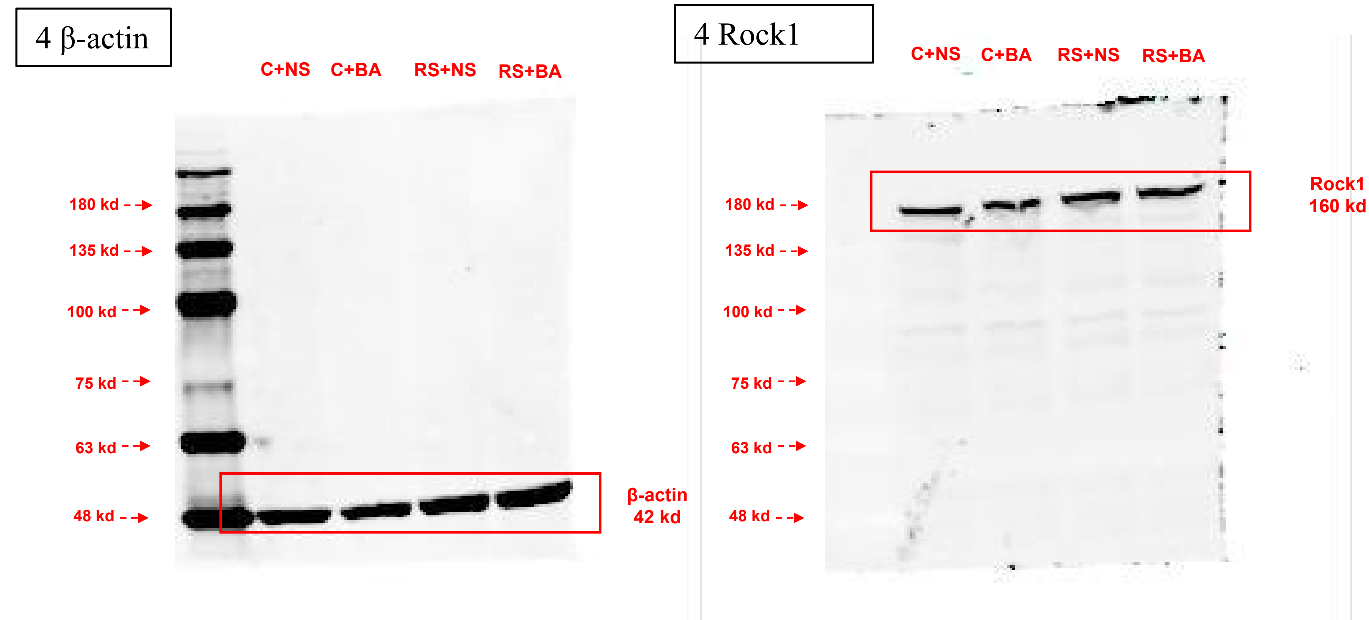
4 β-actin
C+NS C+BA RS+NS RS+BA
180 kd
135 kd
100 kd
75 kd
63 kd
48 kd
β-actin
42 kd
4 Rock1
C+NS C+BA RS+NS RS+BA
180 kd
135 kd
100 kd
75 kd
63 kd
48 kd
Rock1
160 kd

Fig. 8K GPR41

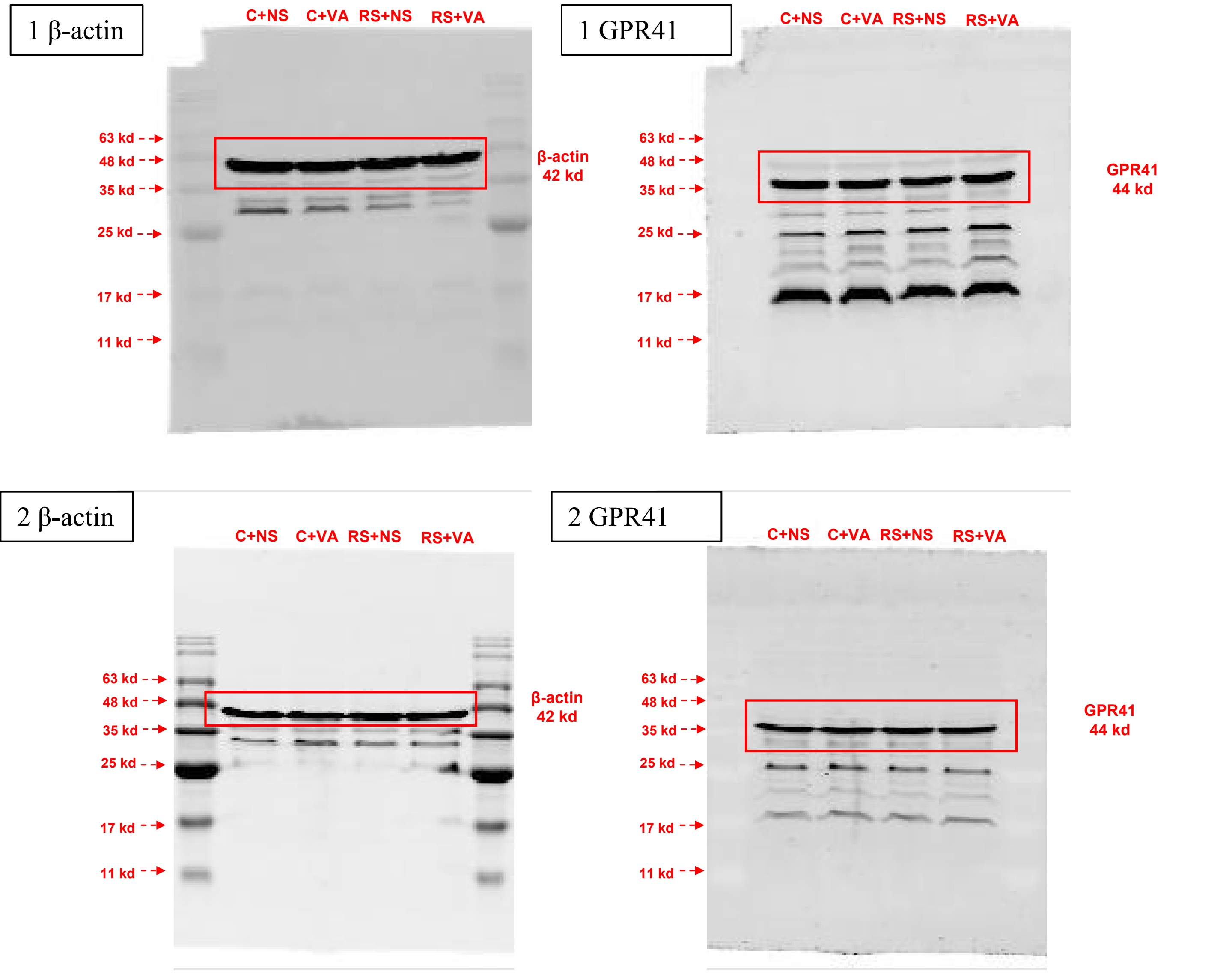
1 β-actin
C+NS C+VA RS+NS RS+VA
63 kd
48 kd
35 kd
25 kd
17 kd
11 kd
β-actin
42 kd
1 GPR41
C+NS C+VA RS+NS RS+VA
63 kd
48 kd
35 kd
25 kd
17 kd
11 kd
GPR41
44 kd
2 β-actin
C+NS C+VA RS+NS RS+VA
63 kd
48 kd
35 kd
25 kd
17 kd
11 kd
β-actin
42 kd
2 GPR41
C+NS C+VA RS+NS RS+VA
63 kd
48 kd
35 kd
25 kd
17 kd
11 kd
GPR41
44 kd

3 β-actin

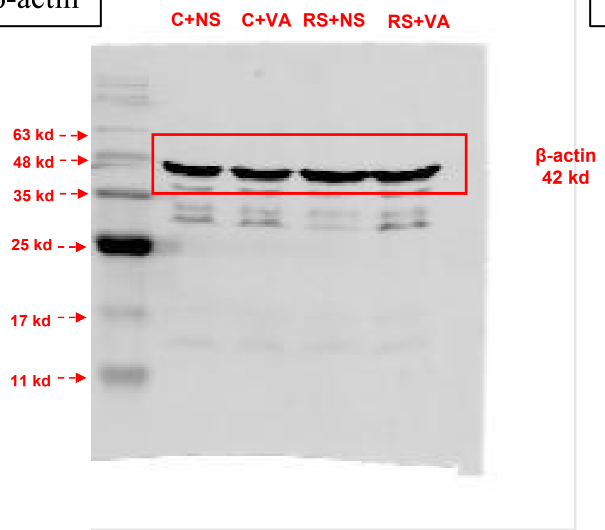

3 GPR41

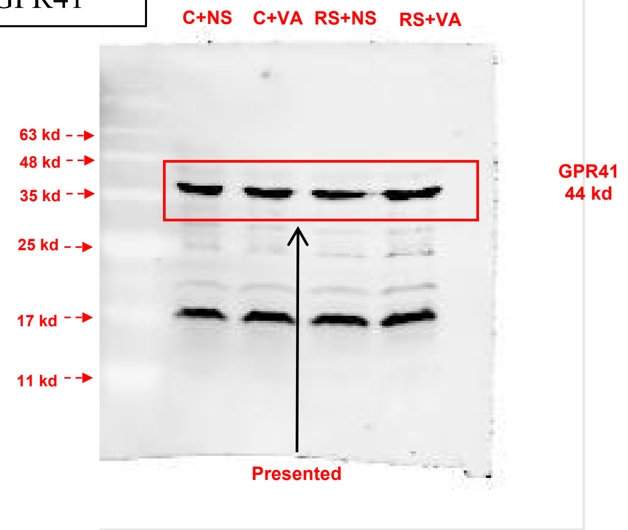

4 β-actin

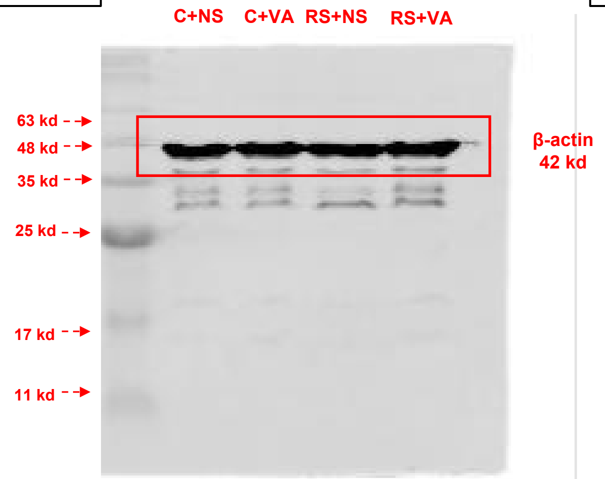

4 GPR41

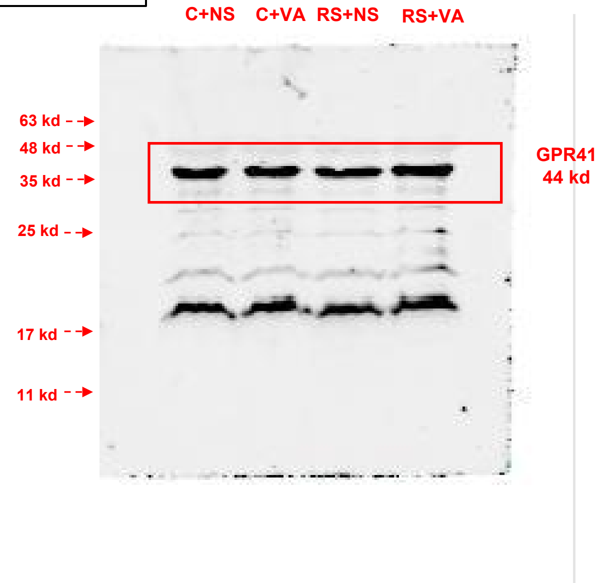

Fig. 8K RhoA

1 β-actin

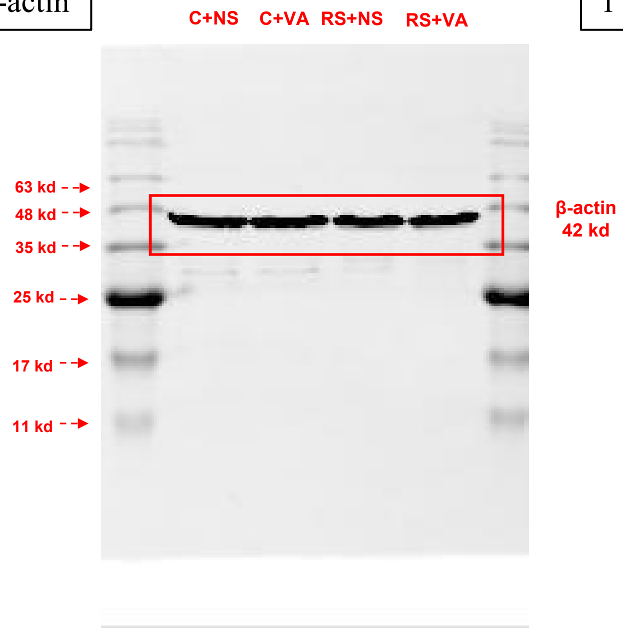

1 RhoA

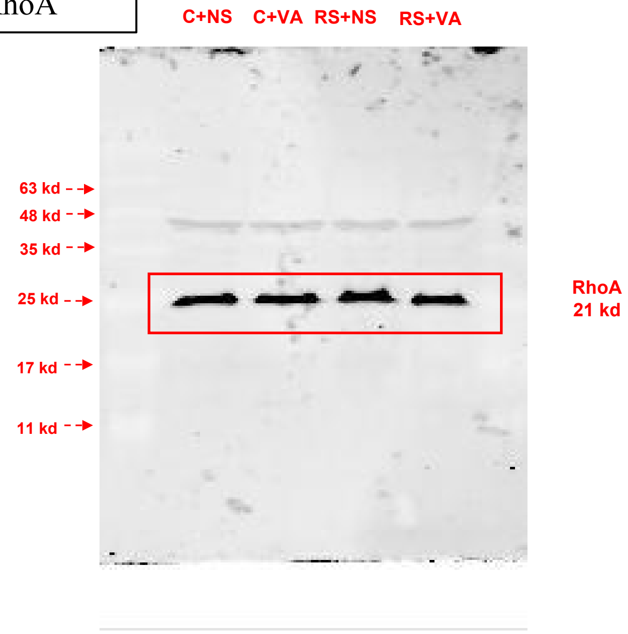

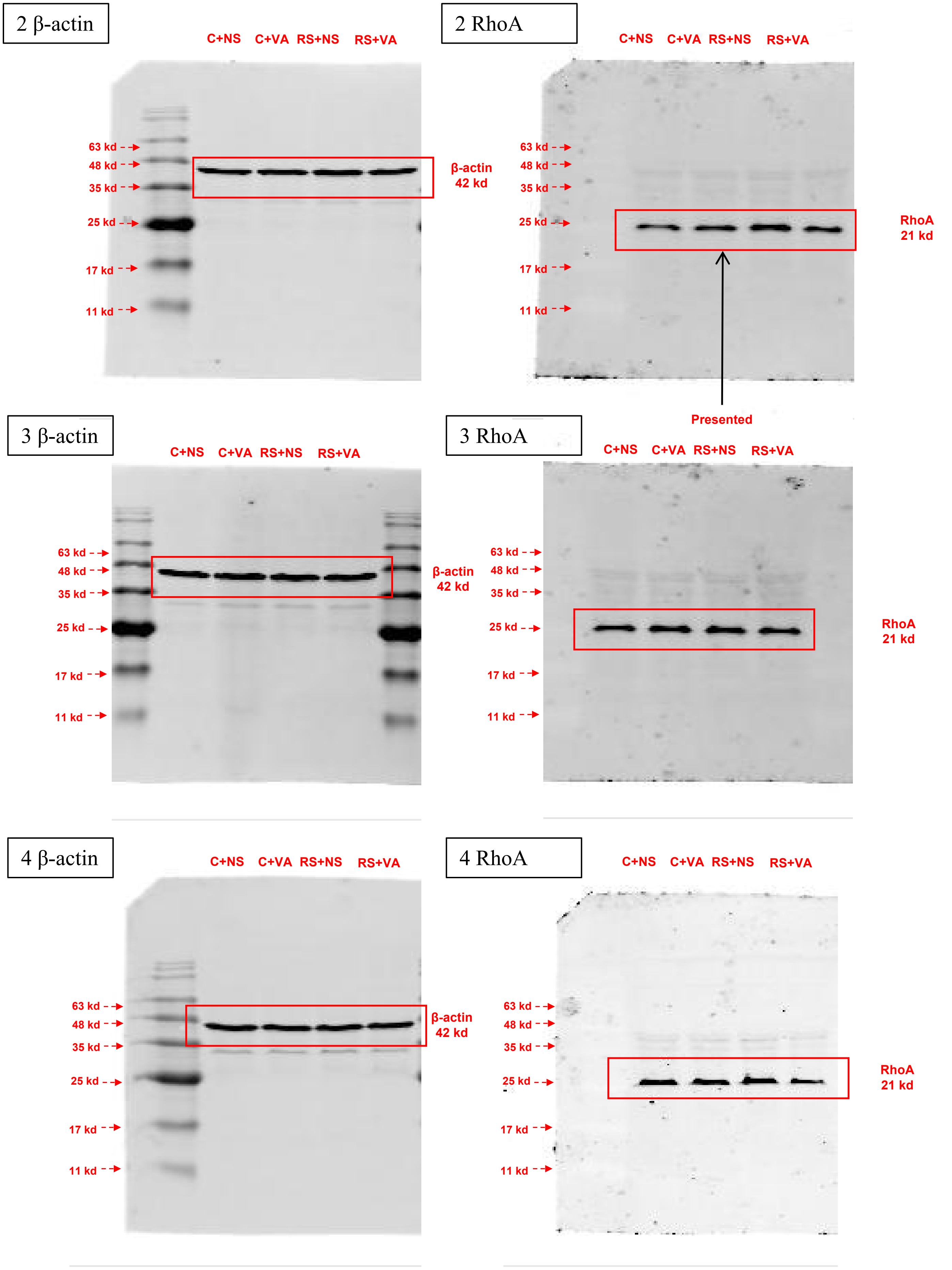
2 β-actin
C+NS C+VA RS+NS RS+VA
63 kd
48 kd
35 kd
25 kd
17 kd
11 kd
β-actin 42 kd
2 RhoA
C+NS C+VA RS+NS RS+VA
63 kd
48 kd
35 kd
25 kd
17 kd
11 kd
RhoA 21 kd
Presented
3 β-actin
C+NS C+VA RS+NS RS+VA
63 kd
48 kd
35 kd
25 kd
17 kd
11 kd
β-actin 42 kd
3 RhoA
C+NS C+VA RS+NS RS+VA
63 kd
48 kd
35 kd
25 kd
17 kd
11 kd
RhoA 21 kd
4 β-actin
C+NS C+VA RS+NS RS+VA
63 kd
48 kd
35 kd
25 kd
17 kd
11 kd
β-actin 42 kd
4 RhoA
C+NS C+VA RS+NS RS+VA
63 kd
48 kd
35 kd
25 kd
17 kd
11 kd
RhoA 21 kd

Fig. 8K Rock1

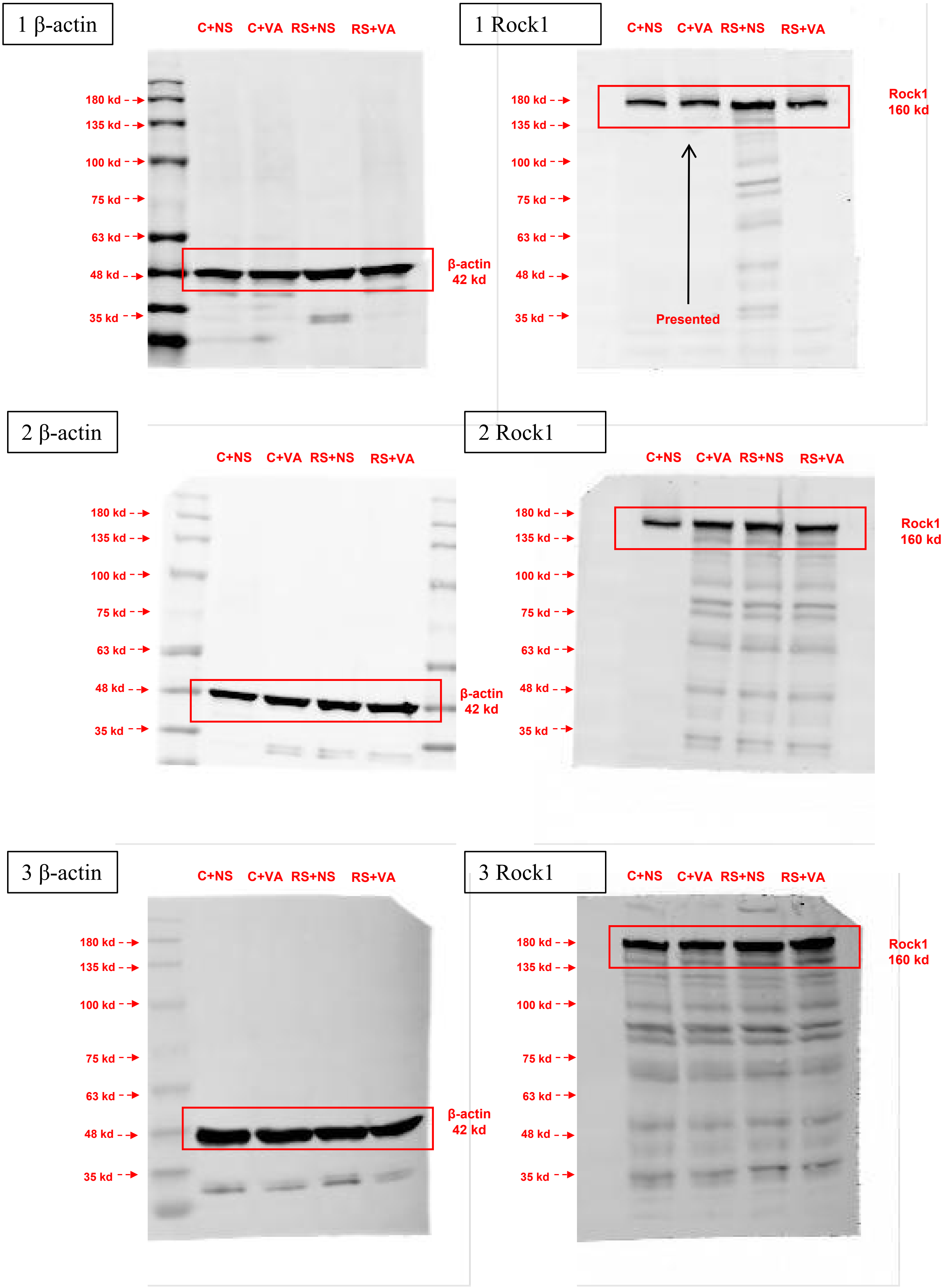
1 β-actin
C+NS
C+VA
RS+NS
RS+VA
180 kd
135 kd
100 kd
75 kd
63 kd
48 kd
35 kd
β-actin
42 kd
1 Rock1
Rock1
160 kd
Presented
2 β-actin
2 Rock1
3 β-actin
3 Rock1

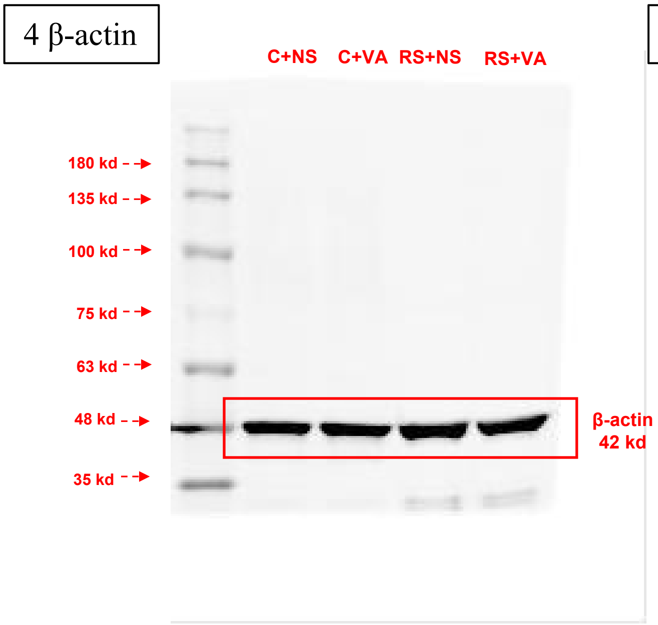
4 β-actin
C+NS
C+VA
RS+NS
RS+VA
180 kd
135 kd
100 kd
75 kd
63 kd
48 kd
35 kd
β-actin
42 kd

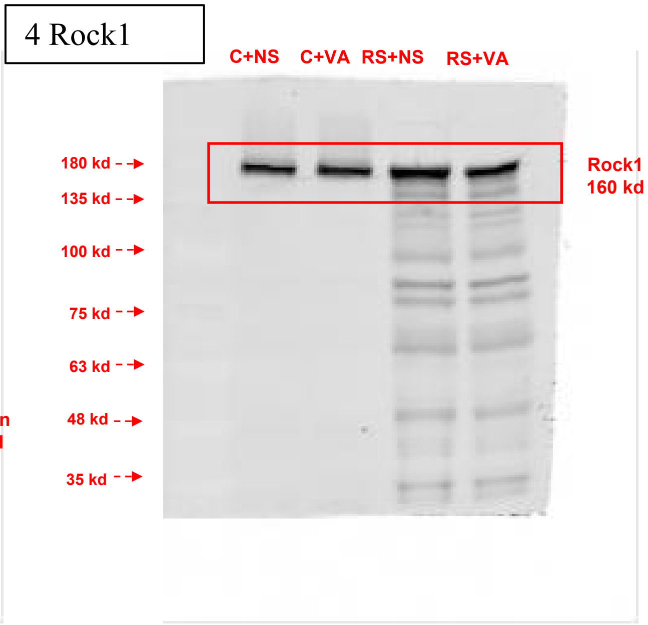
4 Rock1
C+NS
C+VA
RS+NS
RS+VA
180 kd
135 kd
100 kd
75 kd
63 kd
48 kd
35 kd
Rock1
160 kd
