# Supplementary material for: Butyric acid and valeric acid attenuate stress-induced ferroptosis and depressive-like behaviors by suppressing hippocampal neuroinflammation
Source: J Transl Med. 2025 Sep 2;23:974. doi: 10.1186/s12967-025-06950-0 (PMC12403447; doi:10.1186/s12967-025-06950-0)
Supplement: Supplementary file 2 — Supplementary Material 2 [file 12967_2025_6950_MOESM2_ESM.docx]

**Supplemental Information**

**Butyric acid and valeric acid attenuate stress-induced ferroptosis and depressive-like behaviors by suppressing hippocampal neuroinflammation**

Xiaoying Ma ^1†^, Weibo Shi ^1†^, Zhen Wang ^1^, Shujin Li ^1^, Rufei Ma ^1^, Weihao Zhu ^1^, Lin Wu ^1^, Xiaowei Feng ^1^, Bin Cong ^1*^, Yingmin Li ^1*^

^1^ *Collaborative Innovation Center of Forensic Medical Molecular Identification, Hebei Key Laboratory of Forensic Medicine, Department of Forensic Medicine, Hebei Medical University, Shijiazhuang 050017, China.*

^*^ Corresponding authors: Collaborative Innovation Center of Forensic Medical Molecular Identification, Hebei Key Laboratory of Forensic Medicine, Department of Forensic Medicine, Hebei Medical University, Shijiazhuang 050017, China.

Yingmin Li: E-mail: 16000557@hebmu.edu.cn; Tel: + 86 311 8626 1004.

Bin Cong: E-mail: cong6406@hebmu.edu.cn; Tel: + 86 311 8626 6406.

^†^ These authors contributed equally to this work.This supplementary document including:

**Fig. S1.** Ferroptosis inhibitors improve hippocampal neuronal damage in stressed mice.

**Fig. S2.** Gut microbiota from stressed mice induces hippocampal neuronal damage in germ-free mice.

**Fig. S3.** Gut microbiota metabolites butyric acid and valeric acid alleviate hippocampal neuronal damage in stressed mice.


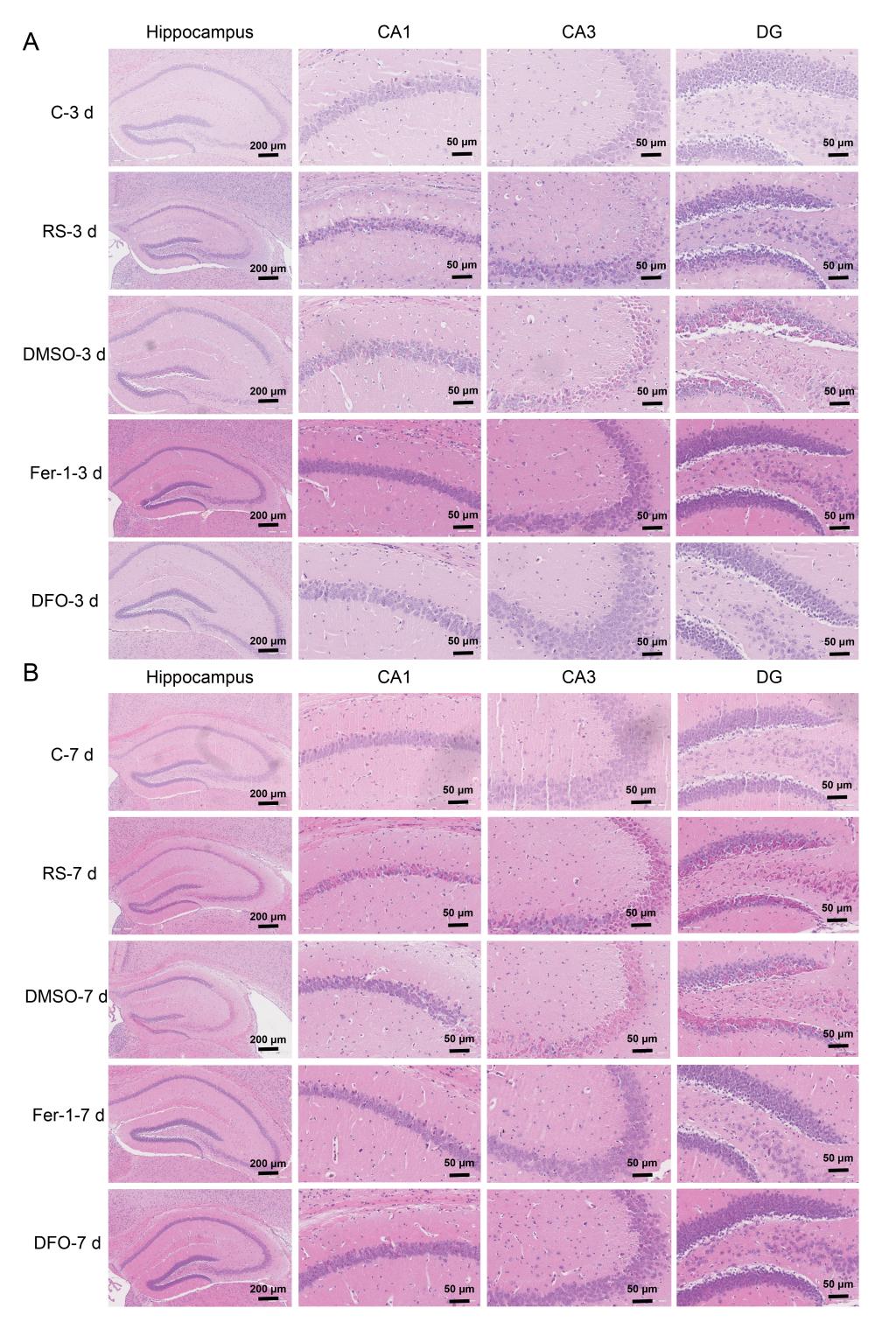


**Fig. S1. Ferroptosis inhibitors improve hippocampal neuronal damage in stressed mice.** (A) Representative HE staining images of hippocampal tissue from each group of mice after treatment with ferroptosis inhibitors Fer-1 and DFO (3 days). (B) Representative HE staining images of hippocampal tissue from each group of mice after treatment with ferroptosis inhibitors Fer-1 and DFO (7 days). Scale bars: 200 μm and 50 μm.


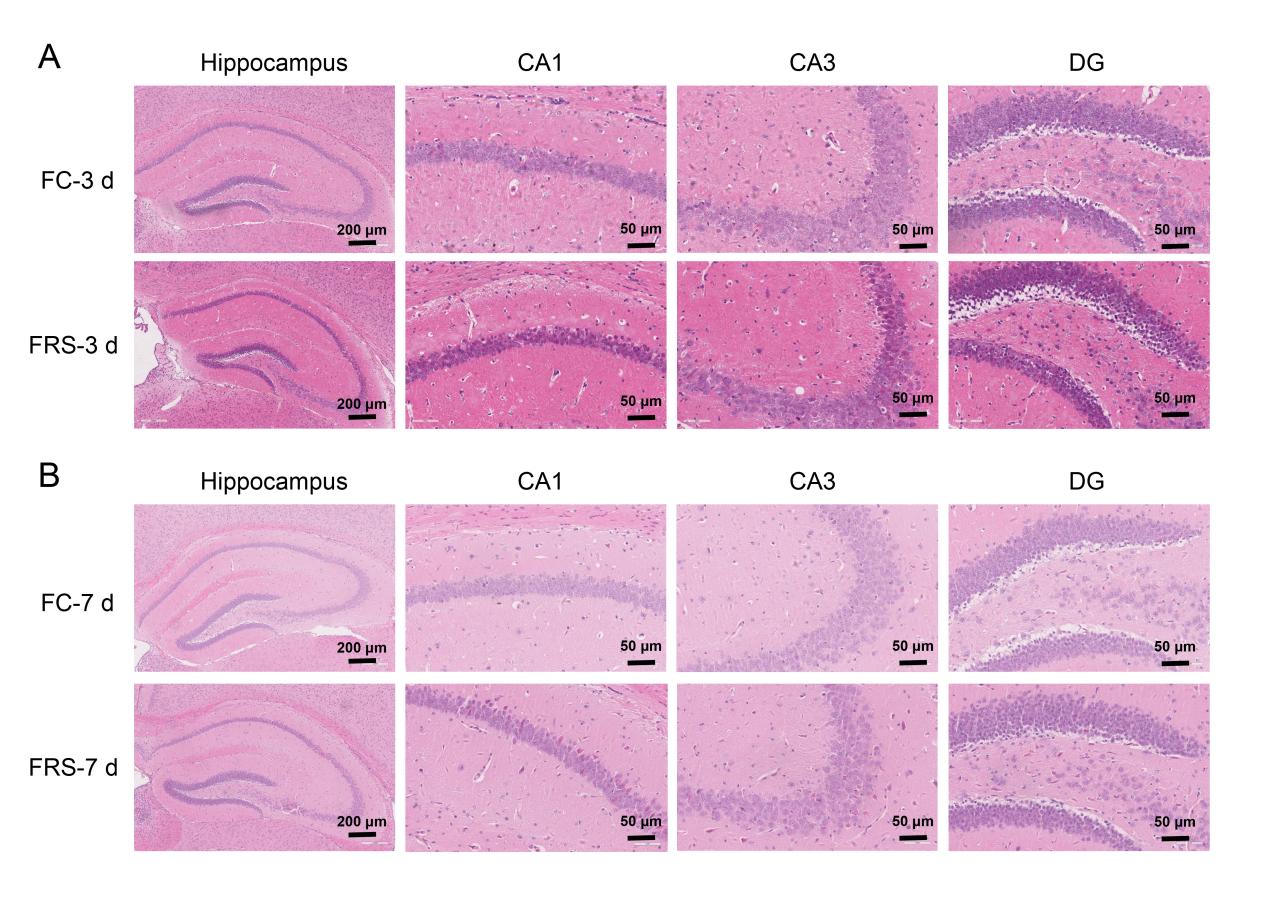


**Fig. S2. Gut microbiota from stressed mice induces hippocampal neuronal damage in germ-free mice.** (A) Representative HE staining images of hippocampal tissue from FC-3 d and FRS-3 d groups of mice. (B) Representative HE staining images of hippocampal tissue from FC-7 d and FRS-7 d groups of mice. Scale bars: 200 μm and 50 μm.


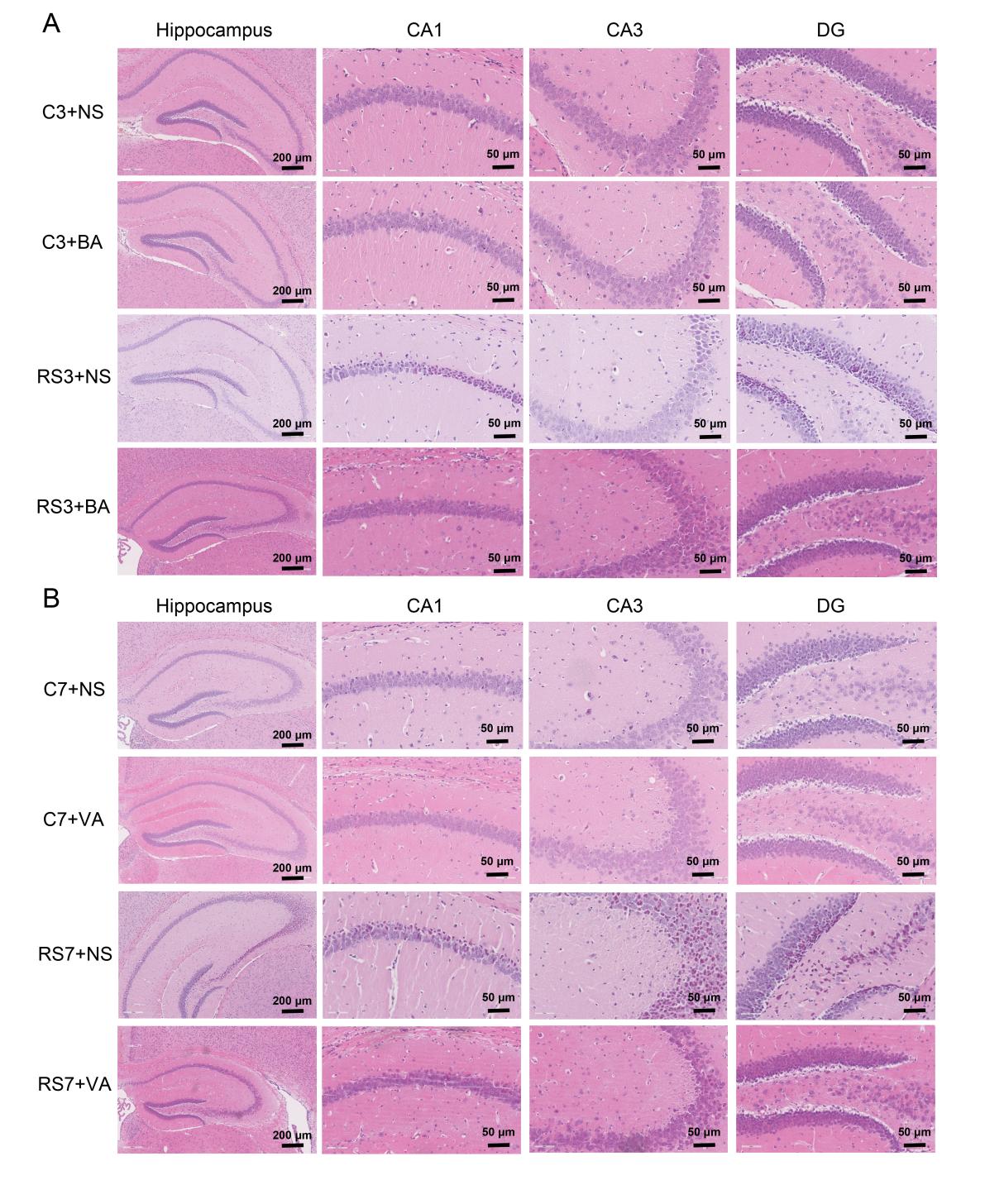


**Fig. S3. Gut microbiota metabolites butyric acid and valeric acid alleviate hippocampal neuronal damage in stressed mice.** (A) Representative HE staining images of hippocampal tissue from each group of mice after butyric acid intervention. (B) Representative HE staining images of hippocampal tissue from each group of mice after valeric acid intervention. Scale bars: 200 μm and 50 μm.
